# DNA barcodes reveal 63 overlooked species of Canadian beetles (Insecta, Coleoptera)

**DOI:** 10.3897/zookeys.894.37862

**Published:** 2019-12-04

**Authors:** Mikko Pentinsaari, Robert Anderson, Lech Borowiec, Patrice Bouchard, Adam Brunke, Hume Douglas, Andrew B.T. Smith, Paul D. N. Hebert

**Affiliations:** 1 Centre for Biodiversity Genomics, 50 Stone Road East University of Guelph, Guelph, ON, N1G 2W1, Canada Centre for Biodiversity Genomics, University of Guelph Guelph Canada; 2 Canadian Museum of Nature, P.O. Box 3443, Station D, Ottawa, ON, K1P 6P4, Canada Canadian Museum of Nature Ottawa Canada; 3 Department of Biodiversity and Evolutionary Taxonomy, University of Wrocław, Przybyszewskiego 65, 51-148 Wrocław, Poland University of Wroclaw Wroclaw Poland; 4 Canadian National Collection of Insects, Arachnids and Nematodes, Agriculture and Agri-Food Canada, 960 Carling Avenue, Ottawa, ON, K1A 0C6, Canada Agriculture and Agri-Food Canada Ottawa Canada

**Keywords:** DNA barcoding, new species records, adventive species

## Abstract

This study demonstrates the power of DNA barcoding to detect overlooked and newly arrived taxa. Sixty-three species of Coleoptera representing 25 families are studied based on DNA barcode data and morphological analysis of the barcoded specimens. Three of the species involve synonymies or previous taxonomic confusion in North America, while the first Canadian records are published for 60 species. Forty-two species are adventive in North America, and 40 of these adventive species originate from the Palaearctic region. Three genera are recorded from the Nearctic region for the first time: *Coelostoma* Brullé, 1835 (Hydrophilidae), *Scydmoraphes* Reitter, 1891 (Staphylinidae), and *Lythraria* Bedel, 1897 (Chrysomelidae). Two new synonymies are established: *Mycetoporustriangulatus* Campbell, 1991 (Staphylinidae) is a junior synonym of *Mycetoporusreichei* Pandellé, 1869, **syn. nov.** while *Blediusphiladelphicus* Fall, 1919 (Staphylinidae) is a junior synonym of *Blediusgallicus* (Gravenhorst, 1806), **syn. nov.** The previously suggested move of *Cteniceratigrina* (Fall, 1901) to the genus *Pseudanostirus* Dolin, 1964 (Elateridae) is formalized, resulting in *Pseudanostirustigrinus* (Fall, 1901), **comb. nov.**

## Introduction

Since being proposed as a standardized approach for identifying unknown specimens to species-level (Hebert et al. 2003) DNA barcoding has become a global research effort. By May 2019, the Barcode of Life Data Systems (BOLD, http://boldsystems.org/; Ratnasingham and Hebert 2007)) contained more than 7 million DNA barcode records. The utility of DNA barcodes for biosecurity and for the detection of invasive species was recognized soon after their initial proposition (Armstrong and Ball 2005). In New Zealand, DNA barcoding has been adopted as a routine tool for screening for new potential pest species in certain insect taxa (Armstrong 2010). Invasive species are one of the most important threats to biodiversity worldwide (Bellard et al. 2016; Maxwell et al. 2016). They are also responsible for major economic losses to agriculture and forestry; invasive species are estimated to cause between $100−200 billion in losses annually in the United States of America alone (Pimentel 2011). Timely detection and accurate identification of new adventive species is important for efficient monitoring and management of potential pests. However, if an adventive species belongs to a taxonomically difficult or neglected group of morphologically similar species, it can remain undetected for decades (deWaard et al. 2010; Jendek et al. 2015).

Among the 8,302 species of Coleoptera known from Canada, 639 are adventive (Brunke et al. 2019). A series of publications by Klimaszewski et al. (2010, 2012, 2013, 2015, 2017) provides both diagnostic features and overviews of the ecology and known North American distribution of these species. While Europe has been and still is a major source of these species because of the intensive trade and ship traffic across the Atlantic, the proportion of Asian species has recently increased as a consequence of increased trade with this region (Klimaszewski et al. 2012). The establishment of Palaearctic species in North America is likely facilitated by the climatic similarity of the regions (Klimaszewski et al. 2013).

The beetle fauna of North and Central Europe has recently been DNA barcoded extensively (Pentinsaari et al. 2014; Hendrich et al. 2015; Rougerie et al. 2015). These barcode reference libraries provide an efficient tool for the detection of adventive European beetle species in other parts of the world, particularly for those taxonomic groups where the native fauna is poorly known. Detection of species shared between continents is greatly facilitated by the Barcode Index Number (BIN) system (Ratnasingham and Hebert 2013). The BIN system was created primarily as an interim taxonomic framework for the COI barcode records on BOLD which lack species level identifications. However, comparison between BINs and Linnaean species names has proved highly useful in uncovering misidentifications, synonymies, and overlooked species diversity (e.g., Landry et al. 2013). In European Coleoptera, BINs showed a perfect 1:1 correspondence to known species in ca. 90% of the studied species (Pentinsaari et al. 2014; Hendrich et al. 2015).

The Canadian arthropod fauna has been extensively sampled for DNA barcoding over the past decade, both in the field and in natural history collections (see e.g., Gwiazdowski et al. 2015, Hebert et al. 2016, Bouchard et al. 2017, Steinke et al. 2017). A joint analysis of all available European and Canadian beetle data allows rapid screening for species shared between continents on a wide taxonomic scale. Such screening in equivalent barcode libraries of Lepidoptera has revealed multiple new species records and synonymies (Landry et al. 2013), as well as previously overlooked species lineages (Mutanen et al. 2012).

This paper reports the first Canadian records for 60 species of beetles, which were initially detected based on DNA barcoded specimens, and resolves previous taxonomic confusion in three more species. Twenty-one species represent native North American taxa recently arrived or previously overlooked in Canada. Forty-two species are adventive, and at least four are potential pests. Two species described from North America were found to be synonyms of Palaearctic species and hence are now properly recognized as adventive to the Nearctic region. We provide morphological diagnoses and illustrations for all adventive species, and for those 12 native North American species for which they are not readily available elsewhere.

## Materials and methods

### Material

This publication is based on the analysis of more than 130,000 DNA barcode records from Europe and Canada. The combined dataset of European and Canadian Coleoptera was screened for intercontinentally shared species. As part of the cleaning and validation process of a barcode reference library for Canadian Coleoptera, representative specimens of Canadian Barcode Index Number (BIN) clusters lacking species-level identifications were retrieved for morphological analysis. After identification and validation of new species records and synonyms, 1168 DNA barcode records (sequence length ≥400 bp) representing 63 species were selected for publication. Most (1147) of these records derive from freshly collected specimens obtained through projects coordinated by the Centre for Biodiversity Genomics, University of Guelph (**CBG**) such as the Canadian National Parks Malaise Program (http://biodiversitygenomics.net/projects/cnp/), the School Malaise Trap Program (Steinke et al. 2017, https://malaiseprogram.com/), and BIObus collecting trips across Canada (https://biobus.ca/). As these specimens are stored pinned or in ethanol in the CBG voucher archive, they were available for morphological study and species assignment once barcode sequences were available. The three specimens of *Attagenussmirnovi* Zhantiev, 1973 were submitted for DNA barcoding through the LifeScanner citizen science initiative (http://www.lifescanner.net/) and are stored in the CBG voucher specimen archive. One of the DNA barcoded specimens of *Contacyphonkongsbergensis* (Munster, 1924) is stored in the Wallis-Roughley Museum of Entomology (**JBWM**). As part of our effort to construct a DNA barcode reference library for Canadian beetles, we analyzed 15,811 specimens of beetles held in the Canadian National Collection of Insects, Arachnids, and Nematodes (**CNC**). Although sequence recovery from the CNC samples was lower than with freshly collected material, their analysis provided a wide set of well-identified reference specimens (Bouchard et al. 2017). Seventeen of the successfully sequenced CNC specimens were found to represent new species for Canada, and are included in the dataset published here.

In addition to the barcoded material, we examined 303 specimens without DNA barcode data to obtain a more detailed understanding of the Canadian distribution of some of the newly detected species. Of these additional specimens, 257 are deposited in CNC, five in the University of Guelph Insect Collection (**DEBU**), four in the Canadian Museum of Nature (**CMNC**), and two in the Field Museum of Natural History, Chicago. Thirty-two additional records of *Notarisscirpi* (Fabricius, 1793) are from specimens deposited in the private insect collections of Claude Chantal (**CCCH**), Stéphane Dumont (**CSDU**), Pierre de Tonnancour (**CPTO**), and Robert Vigneault (**CRVI**). Three additional records of *Carpelimuselongatulus* (Erichson, 1839) are from specimens in the private insect collection of Reginald Webster (**RWC**).

### Tissue sampling and DNA barcode sequencing

The tissue sampling protocol varied according to the origin of the material and size of the specimen. A single leg was detached from each CNC specimen and it was placed in a well in a 96-well microplate pre-filled with 10 µl of 96% ethanol. Each CNC specimen was also photographed, and the resultant image was uploaded to BOLD along with the label data. The specimens archived at the CBG were processed in two ways. Small specimens (body length < 6 mm) were placed into a well in a 96 well microplate for DNA extraction. Following DNA extraction, the microplates were refilled with ethanol and the specimens were stored in the microplates in the CBG voucher specimen archive. Larger specimens were either pinned or preserved in ethanol, and a single leg was used for DNA extraction. Photography of each specimen is not a standard element in the workflow because a million specimens are processed yearly at CBG. Instead, representative specimens of new Barcode Index Numbers (BINs, Ratnasingham and Hebert 2013) are retrieved from the archive for photography once specimens have been assigned to a BIN.

DNA extraction, PCR amplification, and Sanger sequencing of the COI barcode region were performed for all specimens at the Centre for Biodiversity Genomics, using standard protocols optimized for large-scale generation of COI barcode data. For detailed descriptions of the protocols, see Ivanova et al. (2006) and deWaard et al. (2008, 2018). For most specimens, a cocktail of the Folmer primers (Folmer et al. 1994) and LepF1 & LepR1 (Hebert et al. 2004) was used in the first PCR amplification attempt. When resources allowed it, specimens for which the initial PCR amplification failed were reanalyzed using primer sets that targeted 307 bp and 407 bp amplicons (Hajibabaei et al. 2006). Bidirectional sequencing was the rule prior to 2013, but subsequent sequences were often obtained through unidirectional analysis. Details on the PCR and sequencing protocol for each specimen are provided in the public BOLD dataset (see Availability of data below).

### Identification and validation of the new Canadian species records

All COI barcode sequences on BOLD which fulfill quality criteria (< 1% ambiguous bases; no reading frame shifts, chimeras or obvious contaminations) are automatically assigned into BINs. The founding member sequence of a new BIN cluster must be at least 500 bp long, but shorter sequences (min. 300 bp) can be assigned into existing clusters. A detailed description of the clustering algorithm and the associated informatics workflow is provided by Ratnasingham and Hebert (2013). In short, all sequences are initially clustered based on a fixed threshold of 2.2% divergence (uncorrected p-distance). These initial clusters are then refined by Markov clustering to generate the final BINs. The clustering algorithm is run regularly on BOLD, and new BINs are generated and existing BINs may be split or merged as new sequence data are added.

The new adventive species were initially detected because Canadian specimens shared a BIN assignment with their European counterparts. When available, at least five Canadian voucher specimens were then morphologically examined to confirm the identification. Most of the extensions in the known range of native North American species were detected and validated in the same way, i.e., Canadian specimens were found to share BINs with identified specimens from the United States. A few taxa were encountered during the validation of a DNA barcode reference library for Canadian Coleoptera when representative specimens from BINs lacking a species-level identification were retrieved for morphological analysis. Only those species for which voucher specimens were available and could be reliably validated are included in this paper.

### Species accounts

The brief sections on diagnostic information in this paper detail only the most relevant morphological characters for distinguishing each newly detected species from its closest relatives in North America. Due to the variety of beetle taxa covered, these sections cannot employ a completely uniform format. To provide some consistency, the terminology employed and the order in which the characters are presented follows Lawrence et al. (2011). For native North American species with good diagnoses readily available in existing literature, we simply provide references to those diagnoses. In addition to the diagnostic information, we briefly summarize the current Canadian records as well as the distribution of the species outside Canada. Finally, we provide brief ecological data, and consider collecting methods for each species. The order of the families and genera in the species treatments follows Bousquet et al. (2013). Species are ordered alphabetically within genera. We follow Wheeler and Hoebeke (2009) in our use of terms related to non-native species. All distance measures between DNA barcode sequences are reported as uncorrected p-distance (i.e., the proportion of differing nucleotides in pairwise comparisons of sequences).

### Availability of data

Detailed collection information for each specimen, including both DNA barcoded material and other specimen records, as well as GenBank accession numbers for the barcode sequences, are provided in the Suppl. material 1: Table S1. All sequences, details on PCR and sequencing primers, photographs (if available) and full collection data for the DNA barcoded specimens are available through a public dataset on BOLD (DS-NEWCOL18, https://doi.org/10.5883/DS-NEWCOL18). The public BIN pages for each species can be accessed through the BOLD dataset, or by entering the BIN URIs provided in each species account (e.g., BOLD:AAP7843 ) in the search field of the public BIN portal: http://boldsystems.org/index.php/Public_BarcodeIndexNumber_Home

## Results

The higher classification of the new species, and the research projects and collections from which the specimens originate, are summarized in Table 1. Detailed accounts of each species are provided below. The original distribution, habitat preferences, and possible pest status of the adventive species are summarized in Table 2.

**Table 1.** Summary of the higher classification of the studied species and the projects and institutions from which the specimens originate. Abbrevations: SMP: School Malaise Program; CNP & OPPMP: Canadian National Parks & Ontario Provincial Parks Malaise programs; CNC: Canadian National Collection of Insects, Arachnids, and Nematodes.

Family	Subfamily	Species	SMP	CNP& OPPMP	BioBus	Other CBG projects	CNC	Other public & private collections
Gyrinidae	Enhydrinae	*Dineutusemarginatus*			×			
Carabidae	Harpalinae	*Anisodactyluscaenus*					×	
Hydrophilidae	Sphaeridiinae	*Coelostomaorbiculare*				×		
Leiodidae	Leiodinae	*Leiodespolita*				×		
Staphylinidae	Pselaphinae	*Bibloplectusminutissimus*				×		
	Tachyporinae	*Mycetoporusreichei*	×	×				
		*Tachyporusatriceps*	×	×	×	×		
	Aleocharinae	*Amischadecipiens*	×			×		
		*Athetavaga*				×		
	Aleocharinae	*Myllaenainfuscata*				×		
	Oxytelinae	*Blediusgallicus*		×		×		
		*Carpelimuselongatulus*			×	×		×
	Scydmaeninae	*Stenichnuscollaris*			×	×		
		*Stenichnusscutellaris*	×	×	×	×		
		*Scydmoraphesminutus*		×		×		
		*Scydmaenusrufus*				×		
	Paederinae	*Lathrobiumgeminum*			×			
		*Lathrobiumlineatocolle*		×		×		
		*Medonapicalis*				×		
		*Medonripicola*		×				
		*Pseudomedonobscurellus*		×				
Scarabaeidae	Melolonthinae	*Phyllophagaimplicita*			×			
Clambidae	Calyptomerinae	*Calyptomerusdubius*	×			×		
	Clambinae	*Clambussimsoni*	×					
Scirtidae	Scirtinae	*Contacyphonkongsbergensis*			×	×		×
		*Contacyphonobscurellus*		×	×			
		*Contacyphonfuscescens*		×	×	×		
Throscidae	Throscinae	*Aulonothroscusdistans*	×	×		×		
		*Trixaguscarinifrons*		×		×		
		*Trixagusmeybohmi*	×			×		
Elateridae	Dendrometrinae	*Pseudanostirustigrinus*		×				
Cantharidae	Cantharinae	*Dichelotarsuslapponicus*		×				
	Malthininae	*Malthodespumilus*		×	×			
Dermestidae	Attageninae	*Attagenussmirnovi*				×		
Ptinidae	Dorcatominae	*Petaliumincisum*				×		
Erotylidae	Cryptophilinae	*Cryptophilusobliteratus*	×			×		
		*Cryptophiluspropinquus*				×		
Cryptophagidae	Cryptophaginae	*Henoticusmycetoecus*		×				
Phalacridae	Phalacrinae	*Acylomusergoti*	×	×	×	×		
		*Olibrusliquidus*	×					
Nitidulidae	Epuraeinae	*Epuraeaunicolor*			×	×		
Coccinellidae	Chilocorinae	*Chilocorusrenipustulatus*	×					
	Coccinellinae	*Nephusbisignatus*					×	
		*Scymnusrubromaculatus*	×			×		
Corylophidae	Corylophinae	*Orthoperuscorticalis*				×		
Mycetophagidae	Mycetophaginae	*Litargusconnexus*	×					
Ciidae	Ciinae	*Cisboleti*				×		×
		*Cisglabratus*			×			
Mordellidae	Mordellinae	*Mordellistenamilitaris*		×				
Zopheridae	Colydiinae	*Lasconotussubcostulatus*		×			×	
Tenebrionidae	Alleculinae	*Isomiraangusta*			×			
Chrysomelidae	Galerucinae	*Chaetocnemahortensis*	×	×		×	×	
		*Longitarsuslewisii*	×					
		*Lythrariasalicariae*				×		
		*Scelolyperusliriophilus*		×				
Curculionidae	Brachycerinae	*Notarisscirpi*					×	×
	Baridinae	*Ampeloglyptersesostris*				×	×	
		*Centrinopushelvinus*	×					
	Ceutorhynchinae	*Ceutorhynchusinaffectatus*				×		
		*Ceutorhynchusmutabilis*		×			×	
	Cryptorhynchinae	*Peracallespectoralis*			×			
	Entiminae	*Exomiastrichopterus*				×		
	Scolytinae	*Ambrosiodmusrubricollis*		×	×			

**Table 2.** Summary of the original distribution, habitats, and possible pest status of the adventive species.

Family	Species	Original distribution	Habitat	Possible pest status
Cantharidae	*Malthodespumilus*	Palaearctic	Dry meadows, warm forest edges, larvae probably in dead wood as predators	–
Chrysomelidae	*Chaetocnemahortensis*	Palaearctic	On various species of Poaceae	Recorded as a minor pest of wheat and barley in Europe
	*Longitarsuslewisii*	Palaearctic	On *Plantago* spp., especially *P.major*	–
	*Lythrariasalicariae*	Palaearctic	In wetlands on *Lysimachia* spp.	–
Ciidae	*Cisboleti*	Palaearctic	On polypore fungi, mainly *Trametes* spp.	–
	*Cisglabratus*	Palaearctic	On polypore fungi, main host: *Fomitopsispinicola*	–
Clambidae	*Calyptomerusdubius*	Palaearctic	In decaying plant material	–
	*Clambussimsoni*	Australian	In decaying plant material	–
Coccinellidae	*Chilocorusrenipustulatus*	Palaearctic	Deciduous forests, feeds on scale insects	–
	*Scymnusrubromaculatus*	Palaearctic	Dry, warm habitats, mainly found on Brassicaceae, feeds on aphids	–
Corylophidae	*Orthoperuscorticalis*	Palaearctic	Deciduous forests, in fungus-infested dead wood	–
Curculionidae	*Notarisscirpi*	Palaearctic	On *Scirpus* and *Carex* in various wet habitats	–
	*Exomiastrichopterus*	Palaearctic	Eurytopic, polyphagous, often found in orchards and gardens	Potential pest of berry crops. Recorded as a pest of strawberry, raspberry and black chokeberry in Europe.
	*Ceutorhynchusinaffectatus*	Palaearctic	On *Hesperismatronalis* (also *H.tristis* in Europe)	–
	*Ambrosiodmusrubricollis*	Palaearctic	Ambrosia feeder, polyphagous	Potential pest of various deciduous and coniferous trees.
Dermestidae	*Attagenussmirnovi*	Afrotropical	Larval development mainly (exclusively?) indoors in temperate areas	Pest of various organic materials of animal origin
Erotylidae	*Cryptophilusobliteratus*	Palaearctic	In decaying plant material	–
	*Cryptophiluspropinquus*	Palaearctic	In decaying plant material	–
Hydrophilidae	*Coelostomaorbiculare*	Palaearctic	Aquatic, mainly in eutrophic ponds	–
Leiodidae	*Leiodespolita*	Palaearctic	Eurytopic, in forests, heaths, gardens, etc., larval development probably in subterranean fungi	–
Mycetophagidae	*Litargusconnexus*	Palaearctic	In deciduous and mixed forests in fungus-infested dead wood	–
Nitidulidae	*Epuraeaunicolor*	Palaearctic	Decaying and fermenting fruit, tree sap, fungal fruiting bodies, etc.	–
Phalacridae	*Olibrusliquidus*	Palaearctic	Prefers dry, warm habitats, larval development in flowers of Asteraceae, exact host plants preferences not known	–
Staphylinidae	*Amischadecipiens*	Palaearctic	Eurytopic, in various, usually moist habitats	–
	*Athetavaga*	Palaearctic	Eurytopic, in decaying organic material, often in bird nests	–
	*Myllaenainfuscata*	Palaearctic	At margins of standing and running water	–
	*Blediusgallicus*	Palaearctic	Moist soil at river banks, edges of fields, etc.	–
	*Carpelimuselongatulus*	Palaearctic	Various moist habitats	–
	*Lathrobiumgeminum*	Palaearctic	Moist, open habitats	–
	*Lathrobiumlineatocolle*	Palaearctic	Riparian habitats and wet meadows	–
	*Medonapicalis*	Palaearctic	Exact breeding habitat unknown, collected from various habitats	–
Staphylinidae	*Medonripicola*	Palaearctic	Unknown, possibly deep litter or mammal burrows	
	*Pseudomedonobscurellus*	Palaearctic	Wetlands, in decaying organic matter	
	*Bibloplectusminutissimus*	Palaearctic	Collected in flood debris and along river banks in Europe, exact microhabitat unknown	
	*Scydmaenusrufus*	Palaearctic	Eurytopic, found in forest edges, parks, gardens, floodplains and fields	
	*Scydmoraphesminutus*	Palaearctic	Associated with ants	
	*Stenichnuscollaris*	Palaearctic	Various moist habitats	
	*Stenichnusscutellaris*	Palaearctic	Forests, forest edges, gardens	
	*Mycetoporusreichei*	Palaearctic	Eurytopic, in various terrestrial habitats	
	*Tachyporusatriceps*	Palaearctic	Eurytopic, mainly in disturbed habitats in Canada	
Throscidae	*Trixaguscarinifrons*	Palaearctic	Prefers dry, warm habitats, larval development probably in soil on fungi (possibly mycorrhizal fungi)	
	*Trixagusmeybohmi*	Palaearctic	Larval development probably in soil on fungi (possibly mycorrhizal fungi)	

### Gyrinidae

#### Dineutus emarginatus

Taxon classificationAnimaliaColeopteraGyrinidae

(Say, 1825)

EA0E096E-2847-57AA-8DE3-994E1C9E036B

##### Distribution.

Native to the Nearctic region. Widespread in the eastern United States (Gustafson and Miller 2015).

##### Canadian records.

Ontario: Charleston Lake Provincial Park, 22-Jun-2015 (2 exx, CBG); Charleston Lake Provincial Park, 25-Jun-2015 (2 exx, CBG).

##### Diagnostic information.

See Gustafson and Miller (2015).

##### Bionomic notes.

Recorded from a variety of lotic and lentic freshwater habitats (Gustafson and Miller 2015). The Canadian adult specimens were hand-collected at a boat launch site on the shore of Charleston Lake, and two larvae matched to the adults by barcode sequences were collected with a dip net at a different site in the same lake.

### Carabidae

#### Harpalinae

##### Harpalini

###### Anisodactylus caenus

Taxon classificationAnimaliaColeopteraCarabidae

(Say, 1823)

793F70CB-8CC5-54D8-914C-60857283D830

####### Distribution.

Native to the Nearctic region. Widespread in the United States, recorded from all states bordering southern Ontario (Bousquet 2012).

####### Canadian records.

Ontario: Point Pelee National Park, 08-Jun-2000 (1 ex, CNC).

####### Diagnostic information.

See Lindroth (1968) or Bousquet (2010).

####### Bionomic notes.

This species occurs in deciduous forests on moist soil (Larochelle and Lariviere 2003). The Canadian specimen was caught with a UV light trap on a forest trail in Point Pelee National Park.

####### Comments.

As only a single specimen was captured, it is uncertain whether this species is truly established in Canada.

### Hydrophilidae

#### Sphaeridiinae

##### Coelostomatini

###### Coelostoma orbiculare

Taxon classificationAnimaliaColeopteraHydrophilidae

(Fabricius, 1775)

9534B977-DF95-57DE-947F-F0DC08A5188C

Figure 1

####### Distribution.

Native to the Palaearctic region. Widespread and common in Europe, distributed across Eurasia to the Russian Far East and Japan (Hansen 1987, 2004). Adventive in the Nearctic region (Ontario, Canada).

####### Canadian records.

Ontario: Cambridge, 01-Jun-2015 (1 ex, CBG); Hartington, 18-Apr-2017 (1 ex, CBG).

####### Diagnostic information

**Figure 1.**
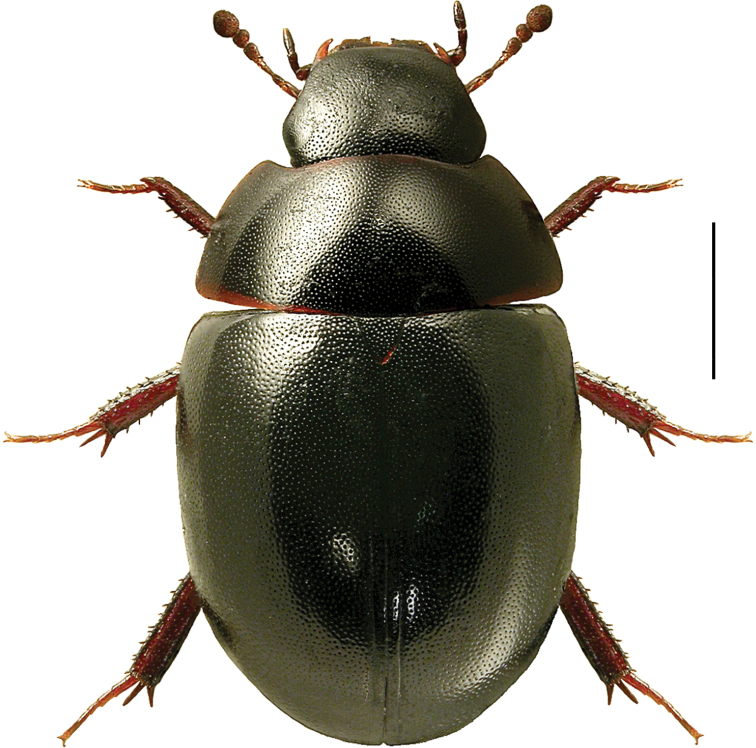
*Coelostomaorbiculare* (Fabricius), habitus, L. Borowiec. Scale bar: 1.0 mm.

(based on Hansen 1987). Body length 4.0–4.8 mm. Habitus short and wide, convex, as in Fig. 1. Black, with the pronotal margins sometimes narrowly red-brown. Antennae with nine antennomeres and a loosely built club with three antennomeres. Base of antennae concealed in dorsal view by the expanded lateral margin of the head. Eyes emarginate. Elytra with sharply impressed sutural striae reaching from apex at least to middle. Tarsomere 1 of meso- and metatarsi longer than tarsomere 2. Abdominal ventrite 1 without medial carina.

####### Bionomic notes.

This species is found in stagnant fresh water. It prefers eutrophic ponds with dense vegetation, and mainly occurs in shallow water at the edges (Hansen 1987). One of the two Canadian specimens was collected as a larva in a leaf litter sample from a wetland, the other (an adult) was sifted from leaf litter close to a lake shore.

####### Comments.

This is the first record of the genus *Coelostoma* Brullé, 1835 in the Nearctic region. *Coelostomaorbiculare* leads to couplet 28 in Van Tassell’s (2001) key to North American genera of Hydrophilidae together with the genera *Dactylosternum* Wollaston, 1854 and *Phaenonotum* Sharp, 1882. It can be distinguished from *Dactylosternum* by the absence of a longitudinal carina on the first abdominal ventrite (present in *Dactylosternum*), and from *Phaenonotum* by the presence of distinct sutural striae on the elytra (absent in *Phaenonotum*).

### Leiodidae

#### Leiodinae

##### Leiodini

###### Leiodes polita

Taxon classificationAnimaliaColeopteraLeiodidae

(Marsham, 1802)

8743139C-16F5-5CD2-993C-80D84E54D4B4

Figure 2

####### Distribution.

Native to the Palaearctic region. Widespread in Europe, also recorded from North Africa and Caucasus (Daffner 1983; Perreau 2004). Adventive in the Nearctic region (Ontario, Canada).

####### Canadian records.

Ontario: Puslinch Township, 15-Aug-2010 to 22-Aug-2010 (1 ex, CBG); Guelph, 18-Aug-2010 (1 ex, CBG).

####### Diagnostic information

**Figure 2.**
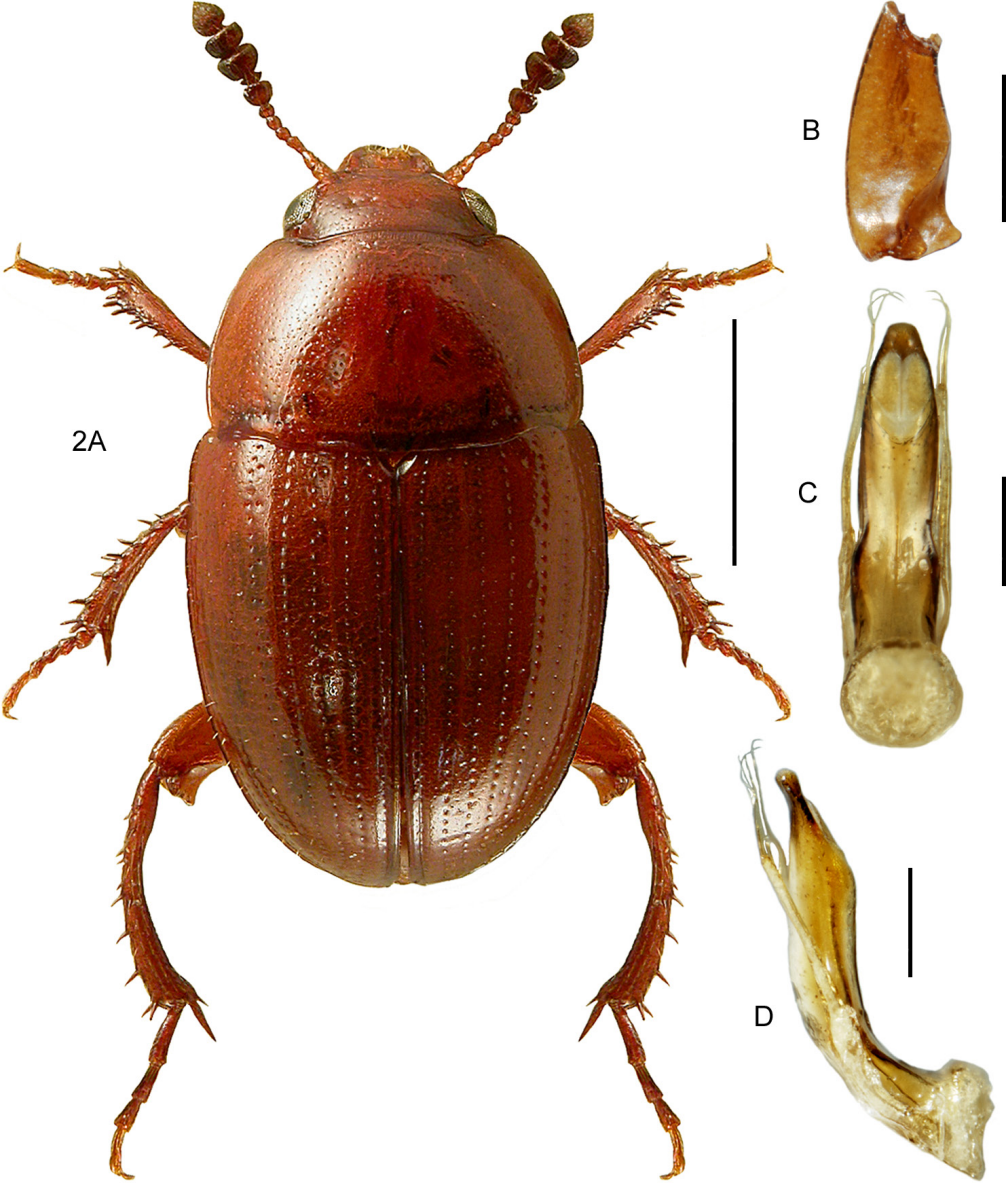
*Leiodespolita* (Marsham) **A** habitus L. Borowiec **B** male left metafemur, dorsal view **C** aedeagus, ventral view **D** aedeagus, lateral view. Scale bars: 1.0 mm (**A**), 0.5 mm (**B**), 0.2 mm (**C, D**).

(based on Daffner 1983). Body length 2.2–4 mm. Habitus as in Fig. 2A. Red-brown or yellow-brown, head, pronotum and the sutural and lateral margins of elytra sometimes darkened. Antennae long, with a strongly transverse, darkened club, last antennomere narrower than antennomere 10. Head normally with four punctures in transverse series. Basal margin of pronotum sinuate laterally. Mesoventrite with a long, low and evenly curved medial carina, not reaching the transverse carina and without excavation anteriorly. Metaventrite approximately as long as abdominal ventrites 1 and 2 combined. Elytra with regular, strongly and densely punctate striae, interstitial punctures sparse. Elytra not transversely strigose or strongly microsculptured. Elytral stria 9 separated from side margin at basal third, forming a subhumeral row of punctures. Protibiae only moderately widened towards apex. Metafemora in both sexes with an apical projection at both inner and outer margins, projections stronger in males (Fig. 2B). Male metatibiae bent inward starting from the basal third. Male genitalia as in Fig. 2C, D.

####### Bionomic notes.

This eurytopic species is found in forests, forest edges, heaths, gardens etc. in Europe (Koch 1989b). The Canadian specimens were collected with Malaise traps in suburban residential areas in southern Ontario.

####### Comments.

*Leiodespolita* leads to *L.quebecensis* Baranowski, 1993 in the key to North American species of *Leiodes* (Baranowski 1993). It can be distinguished by the sinuate basal margin of the pronotum (straight in *L.quebecensis* and related species), differently formed projections of the metafemora, and the male genitalia.

### Staphylinidae

#### Pselaphinae

##### Trichonychini

###### Bibloplectus minutissimus

Taxon classificationAnimaliaColeopteraStaphylinidae

(Aubé, 1833)

EBBF2E1D-0FC3-5808-A93C-C4B6E29BE5CB

Figure 3

####### Distribution.

Native to the West Palaearctic region, widespread in Europe (Schülke and Smetana 2015). Adventive in the Nearctic region (Ontario, Canada).

####### Canadian records.

Ontario: Peterborough, 24-May-2015 to 30-May-2015 (1 ex, CBG); Markham, 24-Jun-2017 to 25-Jun-2017 (1 ex, CBG).

####### Diagnostic information.

**Figures 3, 4.**
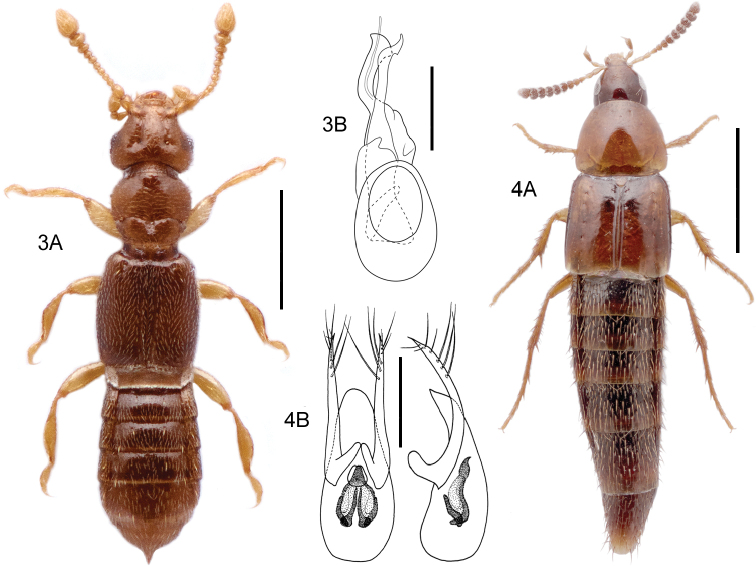
**3***Bibloplectusminutissimus* (Aubé) **3A** habitus **3B** aedeagus, ventral view, re-drawn from Löbl (1960)**4***Mycetoporusreichei* Pandellé **4A** habitus **4B** aedeagus, ventral and lateral view, M. Kocian. Scale bars: 0.25 mm (**3A**), 0.1 mm (**3B**), 1.0 mm (**4A**), 0.2 mm (**4B**).

Body length 0.9–1.1 mm. Habitus as in Fig. 3A. Female apical tergite with distinctive projection (Fig. 3A). Aedeagus as in Fig. 3B (Löbl 1960).

####### Bionomic notes.

Pearce (1957) writes that this species can be collected from the base of grasses and under stones along sandy river banks and in flood debris in Britain. Unlike other European (Pearce 1957) or North American (Owens and Carlton 2017) species of the genus, it does not occur in deep leaf litter or damp moss. Both Pearce (1957) and Besuchet (1955) state that the species is infrequently collected but this may be due to the extremely small size and unknown microhabitat requirements. One of the Canadian specimens was collected with a Malaise trap on farmland, the other was extracted from soil and leaf litter from a mixed habitat of farmland and forest.

####### Comments.

Only female specimens were available from the Nearctic, but they share identical barcode haplotypes with a specimen of *Bibloplectusminutissimus* sampled from Germany. They were also morphologically consistent with the diagnostic characters listed above. In the Palaearctic fauna, females of this species can be recognized by a combination of small size, pale body, temples clearly longer than eyes, and apical tergite produced into a long spine (Besuchet 1955). In the Nearctic region, males are needed for an accurate morphological identification (see Chandler 1990; Owens and Carlton 2017) as many undescribed species are still expected.

#### Tachyporinae

##### Mycetoporini

###### Mycetoporus reichei

Taxon classificationAnimaliaColeopteraStaphylinidae

Pandellé, 1869

80C814C9-AAF3-5E6F-B41F-6763BFB19EFD

Figure 4

= Mycetoporustriangulatus Campbell, 1991, syn. nov.

####### Distribution.

Native to the West Palaearctic region and broadly distributed (Schülke and Smetana 2015). Adventive in the Nearctic region (Massachusetts, New Hampshire, and Wisconsin, United States, and Ontario, Quebec, and New Brunswick, Canada) (Campbell 1991, as *M.triangulatus*).

####### Canadian records

(DNA barcoded specimens). Ontario: Orangeville, 22-Sep-2014 to 03-Oct-2014 (1 ex, CBG); Owen Sound, 21-Aug-2014 to 04-Sep-2014 (1 ex, CBG).

####### Additional Canadian records.

See Campbell (1991) and Brunke et al. (2014) for details of earlier records from Canada and United States (as *M.triangulatus*).

####### Diagnostic information.

Body length: 3.1–4.3 mm. Habitus as in Fig. 4A. Ocular puncture of head located at inner edge of eye. Discal pronotal punctures absent. Elytral disc with only one row of punctures, and elytral microsculpture only distinct in apical half. Aedeagus as in Fig. 4B.

####### Bionomic notes.

In the Nearctic, Campbell (1991) reported this species from a vole nest, spruce litter, car net, flood debris, and from a deciduous forest. Brunke et al. (2014) collected this species (as *M.triangulatus*) from soybean fields and their adjacent hedgerows in Ontario, Canada. The barcoded Canadian specimens were collected with Malaise traps, one in a suburban residential area and the other in grassland habitat.

####### Comments.

Campbell (1991) described *Mycetoporustriangulatus* and stated that it is “almost certainly introduced” but was unable to match it to Palaearctic species available for study. The Palaearctic species of *Mycetoporus* were only recently revised to include the complex sclerites of the internal sac (e.g., Schülke 2012b) and the Nearctic fauna had not been reviewed since. The Finnish specimens in the BIN are identified as *M.clavicornis* (Stephens, 1832), a close relative of *M.reichei*. These specimens may be misidentified (they were identified by MP before the presence of *M.reichei* in Finland was detected) and need to be re-examined.

**Figures 5, 6.**
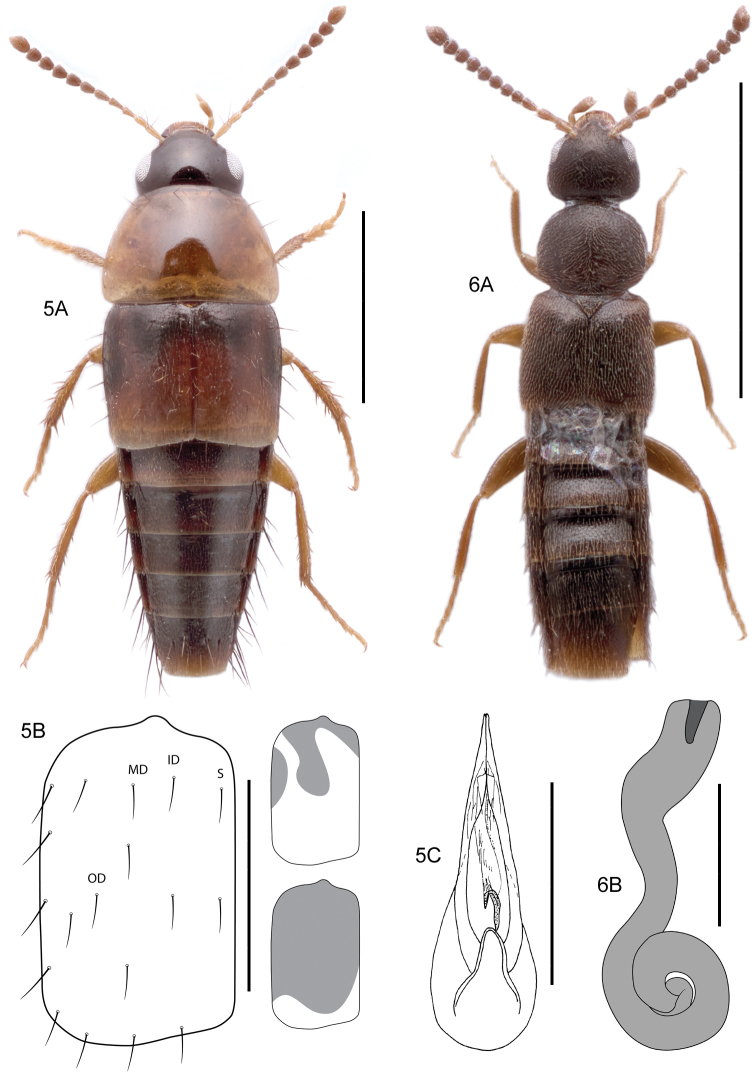
**5***Tachyporusatriceps* Stephens **5A** habitus **5B** elytral chaetotaxy (re-drawn from Assing and Schülke 2012) and pattern variability **5C** aedeagus, ventral view, M. Schülke, modified. Abbreviations: OD outer discal row, MD middle discal row, ID inner discal row, S sutural row **6***Amischadecipiens* (Sharp) **6A** habitus **6B** spermatheca, re-drawn from Muona (1990). Scale bars: 1.0 mm (**5A; 6A**), 0.5 mm (**5B, C**), 0.1 mm (**6B**).

#### Tachyporinae

##### Tachyporini

###### Tachyporus atriceps

Taxon classificationAnimaliaColeopteraCarabidae

Stephens, 1832

88851873-50DB-5276-9C38-83AE92383DC8

Figure 5

####### Distribution.

Native to the Palaearctic region, where it is widespread (Schülke 2012b). Adventive in the Nearctic region (British Columbia, Ontario, Quebec, Nova Scotia, New Brunswick, and Prince Edward Island, Canada).

####### Canadian records

(DNA barcoded specimens). British Columbia: Burnaby, 21-Sep-2015 to 02-Oct-2015 (1 ex, CBG). Ontario: Ancaster, 21-Sep-2015 to 02-Oct-2015 (1 ex, CBG); Ausable-Bayfield Conservation Authority, 30-Jun-2015 (1 ex, CBG); Cambridge, 22-Sep-2014 to 03-Oct-2014 (2 exx, CBG); Cambridge, 24-Apr-2015 to 01-May-2015 (49 exx, CBG); Carillon Park, 06-May-1982 (2 ex, CNC); Courtice, 19-Sep-2016 to 30-Sep-2016 (1 ex, CBG); Guelph, 22-Sep-2014 to 03-Oct-2014 (1 ex, CBG); Guelph, 23-Sep-2013 to 04-Oct-2013 (2 exx, CBG); Guelph, 26-Sep-2014 to 29-Sep-2014 (1 ex, CBG); Hartington, 04-Oct-2017 (1 ex, CBG); Mississauga, 24-May-2016 to 26-May-2016 (1 ex, CBG); Point Pelee National Park, 06-Jul-2015 (1 ex, CBG); Puslinch Township, 19-Sep-2010 to 27-Sep-2010 (2 exx, CBG); Puslinch Township, 24-Oct-2010 to 31-Oct-2010 (1 ex, CBG); Red Rock, 21-Sep-2015 to 02-Oct-2015 (1 ex, CBG); Rondeau Provincial Park, 09-Jul-2015 (1 ex, CBG); Rouge National Urban Park, 03-Jun-2013 to 09-Jun-2013 (13 exx, CBG); Rouge National Urban Park, 15-Sep-2013 (1 ex, CBG); Stayner, 21-Sep-2015 to 02-Oct-2015 (1 ex, CBG). Quebec: Forillon National Park, 16-Sep-2013 to 23-Sep-2013 (2 exx, CBG). New Brunswick: Florenceville-Bristol, 22-Sep-2014 to 03-Oct-2014 (3 exx, CBG); Fredericton, 19-Sep-2016 to 30-Sep-2016 (1 ex, CBG). Nova Scotia: Cape Breton Highlands National Park, 13-Sep-2013 to 20-Sep-2013 (1 ex, CBG); Hubbards, 19-Sep-2016 to 30-Sep-2016 (1 ex, CBG). Prince Edward Island: Prince Edward Island National Park, 11-Sep-2013 to 18-Sep-2013 (1 ex, CBG).

####### Additional Canadian records.

Ontario: Carillon Park, 06-May-1982 (2 exx, CNC); Renfrew County, 4 km SE Cobden, 15-Sep-1980 (1 ex, CNC); Wentworth County, Stoney Creek, 03-Mar-1973 (1 ex, CNC). Quebec: Montreal, 07-Sep-1984 (1 ex, CNC). Nova Scotia: Halifax, 1988 (1 ex, CNC).

####### Diagnostic information.

Body length 2.4–3.6 mm. Habitus as in Fig. 5A. Head black, strongly contrasting with pronotum, elytra with either medial and ovoid lateral markings across disc or disc nearly entirely darkened (Fig. 5B). Elytral chaetotaxy as in Fig. 5B, with sutural row of punctures and three rows of discal punctures, with two punctures in inner discal row. Aedeagus as in Fig. 5C.

####### Bionomic notes.

This species occurs in a variety of moist to very dry microhabitats (Schülke 2012b). In the Nearctic, this species occurs in disturbed habitats. It was collected by Brunke et al. (2014) in soybean fields and adjacent hedgerows in Ontario (misidentified as *T.canadensis* Campbell, 1979, to which it is similar). The barcoded specimens were collected in suburban residential areas and protected land adjacent to cities, mainly with Malaise or pitfall traps. Some of the barcoded specimens are larvae extracted from soil or leaf litter.

####### Comments.

*Tachyporusatriceps* has the same elytral chaetotaxy as *T.borealis* Campbell, 1979, *T.nimbicola* Campbell, 1979, and *T.canadensis* Campbell, 1979 but can be separated from the first two by the elytra with discal markings. *Tachyporuscanadensis* has a dark red-brown head, bright yellow pronotum, and either a pair of narrow linear lateral dark markings (and medial darkening) or entirely immaculate elytra, while *T.atriceps* has a deep black head, slightly darkened (dingy yellow-orange) pronotum and lateral elytral markings that are ovoid or entirely fused with the medial marking to form a broad darkened area over much of the elytra. The internal sac sclerite of *T.atriceps* is similarly shaped to *T.nimbicola* and *T.borealis* (cane-shaped, Fig. 5C) while *T.canadensis* possesses a characteristic arc-shaped sclerite that is not hooked. *Tachyporusatriceps* appears to be common in at least southern Canada and has been present in the Nearctic since at least 1973. This species was probably recently introduced, or has only recently become widespread and common, as it was not detected by Campbell (1979). *Tachyporusatriceps* is split into two closely clustered BINs which show no differences in morphology, including male genitalia.

**Figures 7, 8.**
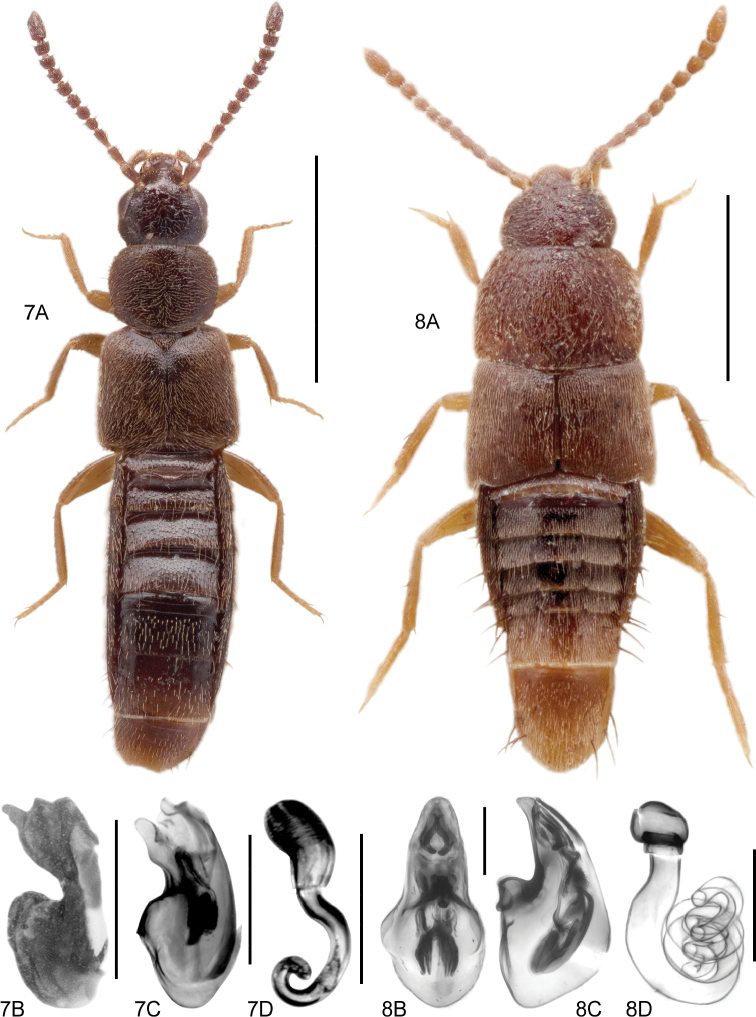
**7A***Athetavaga* (Heer), habitus **7B***Athetavaga*, aedeagus, lateral view **7C***Athetafanatica* Casey, aedeagus, lateral view **7D***Athetafanatica*, spermatheca **8***Myllaenainfuscata* Kraatz **8A** habitus **8B** aedeagus, ventral view **8C** aedeagus, lateral view **8D** spermatheca **8B–D** V. Assing. Scale bars: 1.0 mm (**7A**), 0.2 mm (**7B–D**), 0.5 mm (**8A**), 0.1 mm (**8B–D**).

#### Aleocharinae

##### Athetini

###### Amischa decipiens

Taxon classificationAnimaliaColeopteraStaphylinidae

(Sharp, 1869)

310E6F8C-E2B0-5D8B-9574-6A7E448B58F6

Figure 6

####### Distribution.

Native to the Palaearctic region, occurring broadly in Europe and also reported from the Canary Islands, Tunisia, Turkey, and Mongolia (Schülke and Smetana 2015). Adventive in the Nearctic region (British Columbia and Ontario, Canada).

####### Canadian records.

British Columbia: Abbotsford, 22-Sep-2014 to 03-Oct-2014 (1 ex, CBG); Burnaby, 21-Sep-2015 to 02-Oct-2015 (2 exx, CBG); Port Coquitlam, 20-Apr-2015 to 08-May-2015 (1 ex, CBG); Surrey, 19-Sep-2016 to 30-Sep-2016 (1 ex, CBG). Ontario: Aylmer, 19-Sep-2016 to 30-Sep-2016 (1 ex, CBG); Brantford, 22-Apr-2013 to 03-May-2013 (1 ex, CBG); Cambridge, 20-Apr-2015 to 08-May-2015 (1 ex, CBG); Chesley, 22-Sep-2014 to 03-Oct-2014 (1 ex, CBG); Ethel, 22-Apr-2013 to 03-May-2013 (1 ex, CBG); Georgian Bay Islands National Park, 28-Apr-2013 to 03-May-2013 (1 ex, CBG); Hagersville, 22-Apr-2013 to 03-May-2013 (1 ex, CBG); Hagersville, 23-Sep-2013 to 04-Oct-2013 (1 ex, CBG); Little Britain, 19-Sep-2016 to 30-Sep-2016 (1 ex, CBG); London, 19-Sep-2016 to 30-Sep-2016 (1 ex, CBG); London, 22-Sep-2014 to 03-Oct-2014 (1 ex, CBG); Manitowaning, 21-Sep-2015 to 02-Oct-2015 (5 exx, CBG); Milverton, 22-Apr-2013 to 03-May-2013 (1 ex, CBG); Napanee, 22-Sep-2014 to 03-Oct-2014 (2 exx, CBG); Perth East, 23-Sep-2013 to 04-Oct-2013 (1 ex, CBG); St. Thomas, 22-Sep-2014 to 03-Oct-2014 (10 exx, CBG); Stayner, 21-Sep-2015 to 02-Oct-2015 (2 exx, CBG); Teeswater, 22-Apr-2013 to 03-May-2013 (1 ex, CBG); Teeswater, 23-Sep-2013 to 04-Oct-2013 (1 ex, CBG); Walkerton, 22-Apr-2013 to 03-May-2013 (2 exx, CBG); Walkerton, 22-Sep-2014 to 03-Oct-2014 (2 exx, CBG); Whitby, 23-Sep-2013 to 04-Oct-2013 (1 ex, CBG).

####### Diagnostic information.

Body length: 2.0–2.2 mm. Habitus as in Fig. 6A. Tergite VII in both sexes without a distinct notch. Spermatheca as in Fig. 6B.

####### Bionomic notes.

This eurytopic species is usually found in moist microhabitats such as leaf litter and moldy hay (Koch 1989a). Good (1995) reported this species from agricultural fields and grasslands in Ireland. Most of the barcoded Canadian specimens were collected with Malaise traps in suburban residential areas.

####### Comments.

One of the most distinctive species of this difficult genus, *A.decipiens* can be recognized by tergite VII lacking a notch in both sexes and by the distinctive spermatheca that bears an elongate capsule (Fig. 6B) (Muona 1990). In at least some parts of its European range (e.g., Ireland), the species is considered to be parthenogenetic (Williams 1969; Good 1995). All examined voucher specimens from the Nearctic were females, suggesting that this species is also parthenogenetic in North America. The genus *Amischa* in North America is unrevised and all Nearctic specimens that cannot be matched to Palaearctic *A.analis* (Gravenhorst, 1802) or *A.decipiens* should be treated as unidentifiable pending a comprehensive study. An examination of all North American types was outside the scope of this study and should ideally be accompanied by further DNA sequencing work of both Nearctic and Palaearctic *Amischa*.

###### Atheta vaga

Taxon classificationAnimaliaColeopteraStaphylinidae

(Heer, 1839)

24BAF663-6F36-5A15-9E4C-0267D64C109D

Figure 7

####### Distribution.

Native to the Palaearctic region, widespread in Europe and reported from Algeria, Tunisia, East and West Siberia, and Mongolia (Schülke and Smetana 2015). Adventive in the Nearctic region (California, United States, and Nova Scotia, Canada).

####### Canadian records.

Nova Scotia: Halifax, 30-May-2013 to 06-Jul-2013 (2 exx, CBG).

####### Diagnostic information.

Body length 2.5–2.8 mm. Habitus as in Fig. 7A. Aedeagus as in Fig. 7B. Spermatheca as in *Athetafanatica* Casey (Fig. 7D).

####### Bionomic notes.

Palm (1970) wrote that the species is common at sap runs on trees, on carrion, in fungi, in compost, and in the nests of birds, including ravens and birds of prey. It consistently occurs in a wide variety of bird nests in Europe (Hicks 1959). Its sister species, native Nearctic *A.fanatica* Casey, 1910, apparently lives in the same way (Klimaszewski et al. 2018) and has been collected in artificial owl nest boxes (Majka et al. 2006, Webster et al. 2009). The Canadian specimens were collected with a Malaise trap in a forested part of Point Pleasant Park in Halifax, Nova Scotia.

####### Comments.

First reported from North America by Muona (1984) from California (without specimen data including date). This is the first record from eastern North America and for Canada. Populations in eastern and western North America may represent separate introductions, and dissection and sequencing of further material may reveal a more detailed introduction history. With the exception of its native sister species, *A.fanatica*, *A.vaga* can be easily recognized by the shape of the median lobe in lateral view and spermatheca. It can be distinguished from *A.fanatica* by the less strongly sinuate tubus of the median lobe in lateral view (compare with Fig. 7C). The spermathecae of the two species are identical. The close relationship and separate species status of the two species is confirmed by two well-separated BINs. Although the two examined vouchers from Canada are females, we are confident of their identity based on identical DNA barcode haplotypes shared with European material of *A.vaga*. *Athetafanatica* forms a separate BIN cluster (BOLD:ACL9881 ) which shows ca. 10% divergence from *A.vaga*.

#### Aleocharinae

##### Myllaenini

###### Myllaena infuscata

Taxon classificationAnimaliaColeopteraStaphylinidae

Kraatz, 1853

A2A7C7EF-BD70-555C-9C87-705C991415B2

Figure 8

####### Distribution.

Native to the western Palaearctic region, widely distributed in Europe but rare in the north (Palm 1968) and also reported from India (Kashmir) (Schülke and Smetana 2015), though many of these records need confirmation (Assing 2018). It has previously been reported as occurring in the Nearctic region by Schülke and Smetana (2015) but this is probably based on an erroneous synonymy of North American *M.immunda* Casey with this species in older literature. Klimaszewski (1982) corrected the synonymy, and *M.immunda* is now considered a synonym of *M.arcana* Casey, 1911. The true *M.infuscata* is reported here as adventive in the Nearctic region (Ontario, Canada).

####### Canadian records.

Ontario: Rouge National Urban Park, 15-Sep-2013 (1 ex, CBG).

####### Diagnostic information.

Body length 1.2–1.5 mm. Habitus as in Fig. 8A. Aedeagus as in Fig. 8B, C. Spermatheca as in Fig. 8D.

####### Bionomic notes.

*Myllaenainfuscata* occurs in both exposed and shaded microhabitats along the margins of still and running water (Reid 1991). The Canadian specimen was extracted from soil and leaf litter collected near the mouth of Rouge River.

####### Comments.

*Myllaenainfuscata* is distinctive in the Nearctic fauna for its spermathecal shape, which forms concentric circular coils (Fig. 8D). The median lobe of the aedeagus in lateral view is also distinctive among species in North America (Fig. 8B, C). Within the western Palaearctic fauna, only *M.minuta* has similar genitalia but differs in the shape of the median lobe in lateral view. The single female from Ontario bears the characteristic spermatheca and its corresponding barcode sequence falls within the BIN associated with *M.infuscata*, rather than *M.minuta*, its similar sister species. It is unknown whether this is a recent introduction to North America or if its small size has impeded its detection.

#### Oxytelinae

##### Blediini

###### Bledius gallicus

Taxon classificationAnimaliaColeopteraStaphylinidae

(Gravenhorst, 1806)

6B6DD97A-AE0F-5387-875D-363D09EF30EE

Figure 9

= Blediusphiladelphicus Fall, 1919, syn. nov.

####### Distribution.

Native to the Palaearctic region, trans-Palaearctic (Schülke 2012a). Adventive in the Nearctic region (Maryland, Massachusetts, New Jersey, New York, and Pennsylvania, United States, and Ontario, Quebec, New Brunswick, and Newfoundland, Canada (Herman 1972, Bousquet et al. 2013, as *B.philadelphicus*).

####### Canadian records

(DNA barcoded specimens). Ontario: Georgian Bay Islands National Park, 19-Aug-2013 to 27-Aug-2013 (1 ex, CBG); Grundy Lake Provincial Park, 13-Jul-1995 (1 ex, CNC); Hamilton, 21-Jul-2017 (3 exx, CBG). Quebec: Montreal, 19-Aug-1981 (1 ex, CNC).

####### Additional Canadian records.

See Herman (1972) for a list of earlier records from Canada and the United States (as *B.philadelphicus*).

####### Diagnostic information.

**Figures 9, 10.**
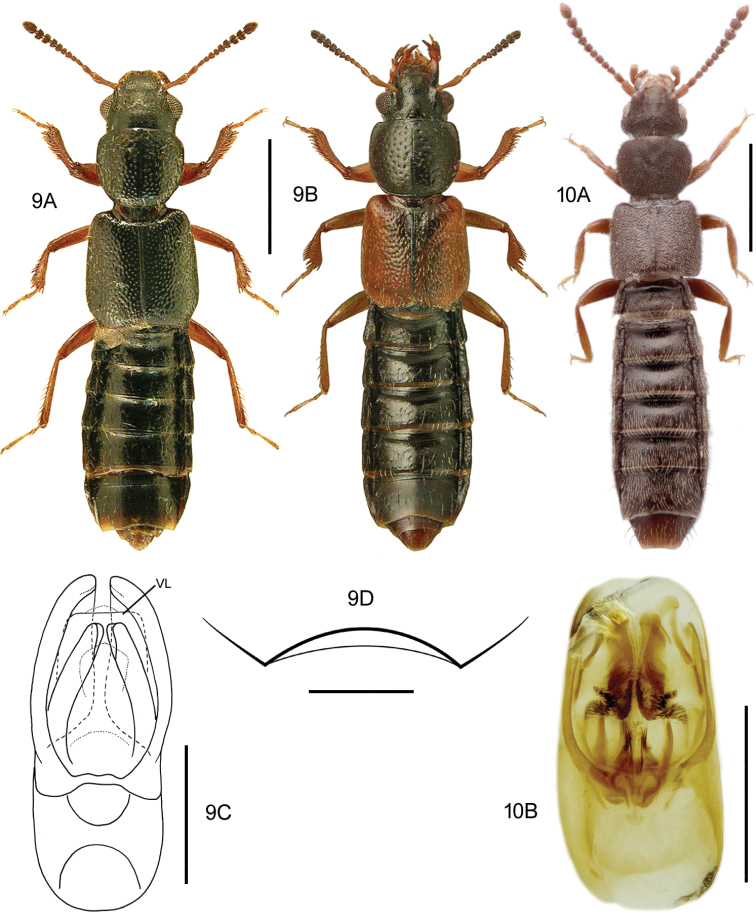
**9***Blediusgallicus* (Gravenhorst) **9A** habitus, black elytra, L. Borowiec **9B** habitus, red-brown elytra, L. Borowiec **9C** aedeagus, ventral view, VL = ventral lamella M. Schülke **9D** male sternite VII **10***Carpelimuselongatulus* (Erichson) **10A** habitus **10B** aedeagus, ventral view, H. Schillhammer. Scale bars: 1.0 mm (**9A, B; 10A**), 0.2 mm (**9C**), 0.2 mm (**9D**), 0.25 mm (**10B**).

Body length: 3.7–4.8 mm. Habitus as in Fig. 9A, B. Male sternite VII as in Fig. 9D. Aedeagus as in Fig. 9C.

####### Bionomic notes.

Palm (1961) (as synonym *B.fracticornis*) states that this species can be found in half-moist sand, gravel, clay or mineral soil mixed with humus, with or without vegetation cover. In Central Europe, this species occurs on sandy to muddy river banks, and also in damp field edges (Schülke 2012a). Three of the CBG specimens were collected at a UV light at a forest edge, one was caught in a Malaise trap in a forested peninsula.

####### Comments.

*Blediusgallicus* can be recognized within Herman’s (1972) ‘semiferrugineus group’ using the following combination of characters: last segment of metatarsus in dorsal view gradually expanded to apex, male sternite VII emarginate, with membranous lobe but emargination not bordered by a pair of spines, pronotum with midlongitudinal groove. The species will key easily to *B.philadelphicus* Fall, 1919 in Herman’s (1972) key and we here consider these two species synonyms. Specimens in the CNC identified as *B.philadelphicus* by Lee Herman and included in his revision of the ‘*semiferrugineus* group’ (Herman 1972) were dissected and revealed to be *B.gallicus*. The description of *Blediusphiladelphicus* in Herman (1972) corresponds to that of *B.gallicus* in Schülke (2012a), including the characteristic male sternite VII (though the membranous part is slightly deeper in both Nearctic and Palaearctic populations than indicated by the illustration). *Blediusgallicus* is closely related to the Palaearctic *B.femoralis* (Gyllenhal, 1827) (Schülke 2012a). The two species have extremely similar aedeagi, differing only in the apex of the ventral lamella (Fig. 9C) (acute in *B.femoralis* and broadly truncate in *B.gallicus*). These two species are more easily separated by the shape of male sternite VII (Schülke 2012a).

Based on the specimens available at the CNC and reported by Herman (1972), *B.gallicus* has been in North America for quite a long time, since at least as early as 1910, when Fall (1910) first described *B.philadelphicus* as *B.dissimilis* (not Erichson 1840, preoccupied name replaced by Fall (1919)). The earliest Canadian specimens are from the 1920s.

#### Oxytelinae

##### Oxytelini

###### Carpelimus elongatulus

Taxon classificationAnimaliaColeopteraStaphylinidae

(Erichson, 1839)

455EF112-AE32-537C-8714-B471C02E0176

Figure 10

####### Distribution.

Native to the Palaearctic region, distributed from western Europe to the Baikal region of Russia (Schülke 2012a). Adventive in the Nearctic region (Ontario, Quebec, and New Brunswick, Canada).

####### Canadian records

(DNA barcoded specimens). Ontario: Ausable-Bayfield Conservation Authority, 30-Jun-2015 (1 ex, CBG); Indian Point Provincial Park, 28-Jul-2015 (1 ex, CBG); Puslinch Township, 09-May-2010 (1 ex, CBG).

####### Additional Canadian records.

Ontario: Guelph, 10-Apr-2009, (2 exx, DEBU); Minesing Swamp, 26-Jan-2008 (1 ex, DEBU). Quebec: Dorval, 10-Oct-1975, (1 ex, FMNH); Sainte-Foy, 27-May-1976, (1 ex, FMNH). New Brunswick: Charlotte County, 05-Jun-2008 (1 ex, RWC); Jackson falls, 22-May-2010, (1 ex, RWC); Musquash, 07-May-2006 (1 ex, RWC).

####### Diagnostic information.

Body length: 2.0–2.6 mm. Habitus as in Fig. 10A. Aedeagus as in Fig. 10B.

####### Bionomic notes.

This species occurs on banks of waterways, wet meadows, agricultural fields and in damp leaf litter (Schülke 2012a). The Canadian specimens were collected with Malaise traps in forests and extracted from leaf litter from a wetland and a river bank.

####### Comments.

As the Nearctic *Carpelimus* have not been revised in modern times, it is currently necessary to dissect males to match with published illustrations of the aedeagus (see Webster et al. 2016). Although only female voucher specimens from the Nearctic were available for study, they easily key to *C.elongatulus* in Schülke (2012a) and two of the barcoded Canadian specimens share identical haplotypes with European specimens of *C.elongatulus*. Similar but much smaller Palaearctic species such as *C.subtilis* are represented in BOLD and form separate BINs deeply divergent from *C.elongatulus*.

#### Scydmaeninae

##### Cyrtoscydmini

###### Stenichnus collaris

Taxon classificationAnimaliaColeopteraStaphylinidae

(Müller & Kunze, 1822)

A3D13407-94DB-5F51-8251-6103289ED70F

Figure 11

####### Distribution.

Native to the western Palaearctic region, widely distributed in Europe (Schülke and Smetana 2015). Adventive in the Nearctic region (Ontario, Canada).

####### Canadian records.

Ontario: Peterborough, 31-May-2015 to 06-Jun-2015 (1 ex, CBG); Rouge National Urban Park, 03-Jun-2013 to 09-Jun-2013 (2 exx, CBG).

####### Diagnostic information.

**Figures 11, 12.**
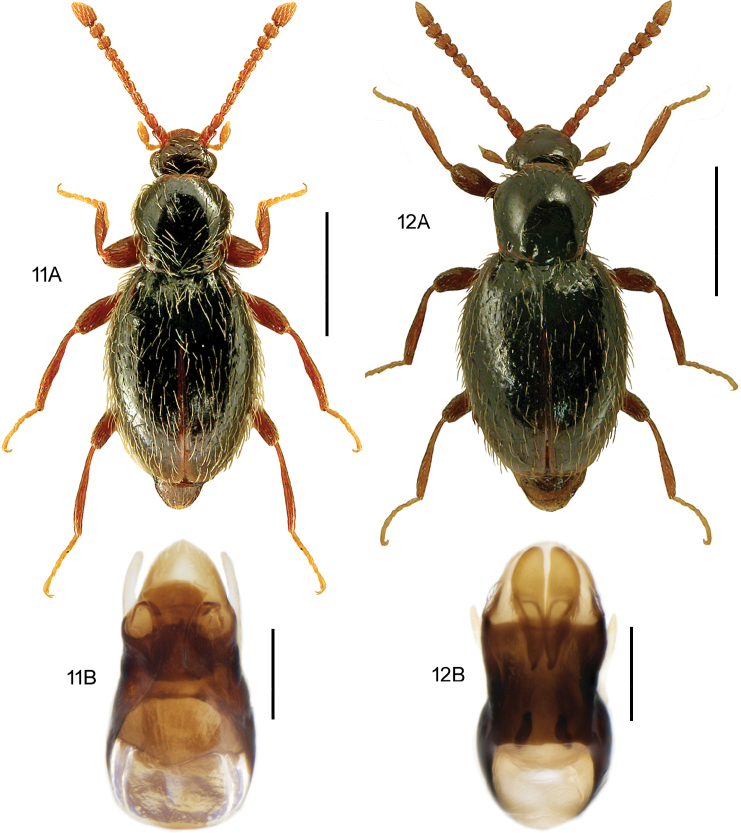
**11***Stenichnuscollaris* (Müller & Kunze) **11A** habitus, L. Borowiec **11B** aedeagus, dorsal view **12***Stenichnusscutellaris* (Müller & Kunze) **12A** habitus, L. Borowiec **12B** aedeagus, dorsal view. Scale bars: 0.5 mm (**11A; 12A**), 0.1 mm (**11B; 12B**).

Body size: 1.55–1.70 mm. Habitus as in Fig. 11A. Aedeagus as in Fig. 11B.

####### Bionomic notes.

Koch (1989a) reports that this species is found in moist forests, forest edges and gardens among leaves and fungi, in dead wood, and at sap flows. One of the Canadian specimens was collected with a Malaise trap on farmland, the other two with pitfall traps in grassland and a river bank.

####### Comments.

As the Nearctic *Stenichnus* fauna remains unrevised, it is only possible to associate Nearctic specimens with Palaearctic species through dissected males or barcodes. The Canadian specimens share identical barcode haplotypes with European material, and the identification was verified by examination of the male genitalia.

###### Stenichnus scutellaris

Taxon classificationAnimaliaColeopteraStaphylinidae

(Müller & Kunze, 1822)

6F4609D1-1430-57D7-9561-88934A2574EF

Figure 12

####### Distribution.

Native to the western Palaearctic region, widespread in Europe (Schülke and Smetana 2015). Adventive in the Nearctic region (Ontario, Canada).

####### Canadian records.

Ontario: Cambridge, 07-May-2015 to 14-May-2015 (5 exx, CBG); Cambridge, 15-Jul-2017 (15 exx, CBG); Cambridge, 25-May-2015 to 01-Jun-2015 (1 ex, CBG); Cambridge, 25-May-2015 to 31-May-2015 (4 exx, CBG); Cambridge, 29-Apr-2015 to 07-May-2015 (1 ex, CBG); Guelph, 13-May-2017 (1 ex, CBG); Guelph, 22-Apr-2013 to 03-May-2013 (2 exx, CBG); Guelph, 22-Apr-2017 (1 ex, CBG); Kitchener, 22-Apr-2013 to 03-May-2013 (1 ex, CBG); Mississauga, 15-Sep-2015 to 17-Sep-2015 (3 exx, CBG); Owen Sound, 22-Apr-2013 to 03-May-2013 (1 ex, CBG); Pickering, 24-Jun-2017 to 25-Jun-2017 (1 ex, CBG); Rouge National Urban Park, 03-Jun-2013 to 09-Jun-2013 (7 exx, CBG); Rouge National Urban Park, 11-Jun-2013 to 18-Jun-2013 (1 ex, CBG); Rouge National Urban Park, 15-Sep-2013 (1 ex, CBG); Rouge National Urban Park, 24-Jun-2017 (1 ex, CBG); Rouge National Urban Park, 29-Apr-2013 to 03-May-2013 (1 ex, CBG); Warsaw, 05-May-2014 to 23-May-2013 (1 ex, CBG); Whitby, 22-Apr-2013 to 03-May-2013 (1 ex, CBG).

####### Diagnostic information.

Body length: 1.4–1.5 mm. Habitus as in Fig. 12A. Male profemur widened apicad to form an abrupt 90° angle in dorsal view. Aedeagus as in Fig. 12B.

####### Bionomic notes.

This species lives in leaf litter and dead wood (Koch 1989a). It is mostly collected in forests or at forest edges, occasionally in wetlands and grasslands (Koch 1989a). Most Canadian specimens were collected using Malaise traps, pitfall traps, or by sifting leaf litter. Most specimens were collected in disturbed forest fragments but some were from grasslands and wetlands.

####### Comments.

As the Nearctic *Stenichnus* fauna remains unrevised, it is only possible to associate Nearctic specimens with Palaearctic species through dissected males or barcodes. The Canadian specimens share identical barcode haplotypes with European material, and the identification was verified by examination of the male genitalia. The modified male profemur of *S.scutellaris* is unique among the Central European fauna (Franz and Besuchet 1971). Without a revision of the Nearctic fauna, it is not possible to know whether other North American species also possess this character.

#### Scydmaeninae

##### Scydmaenini

###### Scydmaenus rufus

Taxon classificationAnimaliaColeopteraStaphylinidae

Müller & Kunze, 1822

4F8AF982-5D75-5F5E-83A6-09856DBB1476

Figure 13

####### Distribution.

Native to the western Palaearctic region, widespread in Europe and also reported from Algeria, Tunisia, and Lebanon (Schülke and Smetana 2015). Adventive in the Nearctic region (Ontario, Canada).

####### Canadian records.

Ontario: Guelph, 17-Sep-2017 (6 exx, CBG).

####### Diagnostic information.

**Figures 13, 14.**
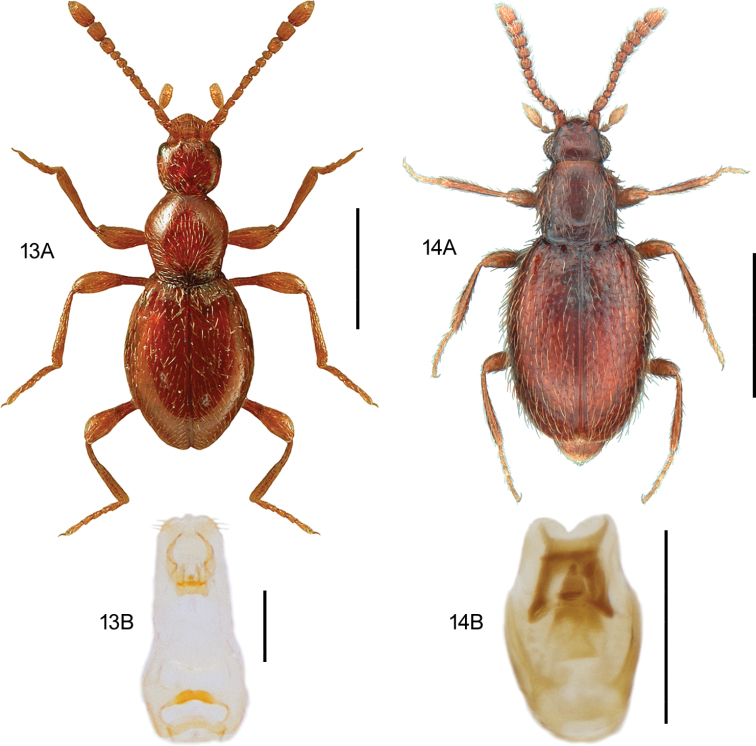
**13***Scydmaenusrufus* Müller & Kunze **13A** habitus, L. Borowiec **13B** aedeagus, ventral view **14***Scydmoraphesminutus* (Chaudoir) **14A** habitus **14B** aedeagus, ventral view. Scale bars: 0.5 mm (**13A; 14A**), 0.1 mm (**13B; 14B**).

Body length: 1.4 mm. Habitus as in Fig. 13A. Aedeagus as in Fig. 13B.

####### Bionomic notes.

This eurytopic species occurs along forest edges and in parks, gardens, floodplains, and fields (Koch 1989a). The Canadian specimens were sifted from a compost heap.

####### Comments.

As the Nearctic *Scydmaenus* fauna remains unrevised, it is only possible to associate Nearctic specimens with Palaearctic species through dissected males or barcodes. Three of the Canadian specimens share identical barcode haplotypes with European material, and the identification was verified by examination of the male genitalia.

#### Scydmaeninae

##### Glandulariini

###### Scydmoraphes minutus

Taxon classificationAnimaliaColeopteraStaphylinidae

(Chaudoir, 1845)

9C48F20C-750C-5B34-9101-4316237AC4D1

Figure 14

####### Distribution.

Native to the Palaearctic region, widespread in Europe and also reported from the Russian Far East (Schülke and Smetana 2015). Adventive in the Nearctic region (Ontario, Canada).

####### Canadian records.

Ontario:Georgian Bay Islands National Park, 30-Jul-2013 to 06-Aug-2013 (1 ex, CBG); Peterborough, 30-Jul-2015 (1 ex, CBG).

####### Diagnostic information.

Body length: 1.15–1.30 mm. Habitus as in Fig. 14A. Head without supraantennal notches or frontal impression, submentum with lateral sutures broadly separated. Pronotum setose, laterally margined, with a transverse groove at the base. Aedeagus as in Fig. 14B.

####### Bionomic notes.

This species is associated with ants, especially species of the *Formicarufa* Linnaeus, 1761 group, and *Lasiusfuliginosus* (Latreille, 1798) and *L.brunneus* (Latreille, 1798) in Europe (Franz and Besuchet 1971). Koch (1989a) reports *S.minutus* with *Lasius* ants in hollow trees, under loose bark and in fallen logs. The Canadian specimens were collected with Malaise traps, one in a forested peninsula and the other on farmland.

####### Comments.

The genus *Scydmoraphes* Reitter, 1891 is here reported for the first time from North America. It was distinguished recently from the similar Nearctic genus *Parascydmus* Casey, 1897 (Jałoszynski 2019), and it does not appear to be an obvious synonym of the other similar Nearctic genus *Brachycepsis* Brendel, 1889. A detailed systematic study of the Nearctic glandulariine genera is warranted. The genus *Scydmoraphes* (with a single species in the Nearctic region) may be recognized within the Nearctic fauna of Glandulariini by the unique combination of a transverse groove on the base of the pronotum, which is margined laterally (Fig. 14A), submentum with lateral sutures broadly separated, and head dorsally lacking frontal impression and supraantennal notches (Jałoszynski 2019). In habitus, *Scydmoraphes* is similar to *Brachycepsis* and *Parascydmus* but can be easily recognized by the transverse pronotal groove.

The following couplets from O’Keefe (2001) were modified to include *Scydmoraphes*:

**Table:** 

19a (18)	Base of pronotum with transverse groove (Fig. 14A)	***Scydmoraphesminutus***
–	Base of pronotum with only impressed basal foveae	**19b**
19b (19a)	Pronotum with 4 basal foveae (fig. 42.20); scutellum large; often light to dark brown in color	***Brachycepsis***
–	Pronotum with 6 basal foveae (fig. 43.20); scutellum minute; often black in color	***Parascydmus***

#### Paederinae

##### Paederini

###### Lathrobium geminum

Taxon classificationAnimaliaColeopteraStaphylinidae

Kraatz, 1857

D76A728E-FA23-54CA-BD79-199DDA1AB7F0

Figure 15

####### Distribution.

Native to the Palaearctic region, distributed from Europe to the Far East of Russia (Schülke and Smetana 2015). Adventive in the Nearctic region (British Columbia, Canada).

####### Canadian records.

British Columbia: Gulf Islands National Park Reserve, 17-Jun-2014 to 22-Jun-2014 (2 exx, CBG).

####### Diagnostic information.

**Figures 15, 16.**
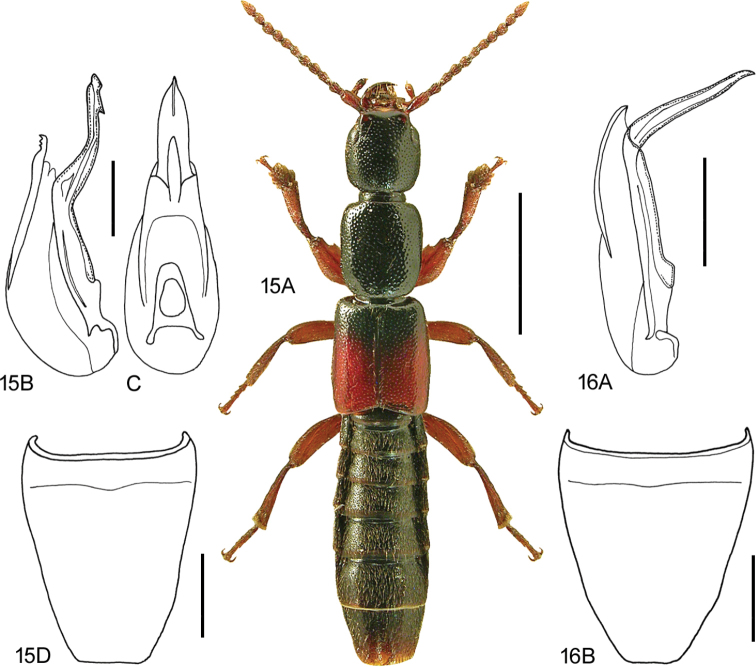
**15***Lathrobiumgeminum* Kraatz **15A** habitus, L. Borowiec **15B** aedeagus, lateral view V. Assing **15C** aedeagus, ventral view V. Assing **15D** female sternite VIII, modified from Assing and Schülke (2012) **16***Lathrobiumlineatocolle* Scriba **16A** aedeagus, lateral view, V. Assing **16B** female sternite VIII, V. Assing. Scale bars: 2.0 mm (**15A**), 0.5 mm (**15B–D**), 0.5 mm (**16A, B**).

Body length: 8.0–9.0 mm. Habitus as in Fig. 15A. Female sternite VIII elongate and truncate apically, as in Fig. 15D. Aedeagus distinctive in lateral view, as in Fig. 15B, C.

####### Bionomic notes.

In Central Europe, this is a common species in unforested, humid microhabitats such as wetlands, shorelines, agricultural fields, gardens, and heath (Assing 2012). The Canadian specimens were collected in a wetland adjacent to a lake, one with pitfall traps and the other by Berlese funnel extraction.

####### Comments.

The voucher specimens from North America are, unfortunately, females but share identical barcode haplotypes with Palaearctic specimens of *L.geminum* from Germany and Finland. North American vouchers key to *L.geminum* in Assing (2012) and female sternite VIII is consistent with the shape described for this species. As the Nearctic fauna of *Lathrobium* is unrevised, comparisons with North American species are not yet possible.

###### Lathrobium lineatocolle

Taxon classificationAnimaliaColeopteraStaphylinidae

Scriba, 1859

4E692C81-F9D3-50EA-9BF7-917AC9D355D7

Figure 16

####### Distribution.

Native to the Palaearctic region, widespread in Europe and reported from China, Iran, Turkey, and the Russian Far East (Schülke and Smetana 2015). Adventive in Canada (Ontario).

####### Canadian records.

Ontario: Rouge National Urban Park, 29-Apr-2013 to 03-May-2013 (1 ex, CBG); Rouge National Urban Park, 03-Jun-2013 to 09-Jun-2013 (1 ex, CBG).

####### Diagnostic information.

Female sternite VIII elongate to narrow, scarcely emarginate apex, as in Fig. 16B. Aedeagus distinctive in lateral view, as in Fig. 16A.

####### Bionomic notes.

In Central Europe, this species occurs mostly in riparian habitats and in wet meadows (Assing 2012). One of the Canadian specimens was collected with a Malaise trap in a forest patch; the other was caught in a riverside pitfall trap.

####### Comments.

As the Nearctic fauna of *Lathrobium* is unrevised, comparisons with North American species are not yet possible.

###### Medon apicalis

Taxon classificationAnimaliaColeopteraStaphylinidae

(Kraatz, 1857)

900DB771-16BD-5470-9759-4BEAABEBACD3

Figure 17

####### Distribution.

Native to the western Palaearctic region, widespread in Europe and also reported from Algeria, Morocco, Turkey, the Canary Islands, and Madeira (Schülke and Smetana 2015). Adventive in the Nearctic region (Ontario, Canada).

####### Canadian records.

Ontario: Guelph, 30-Jun-2018 (1 ex, CBG).

####### Diagnostic information.

**Figures 17–19.**
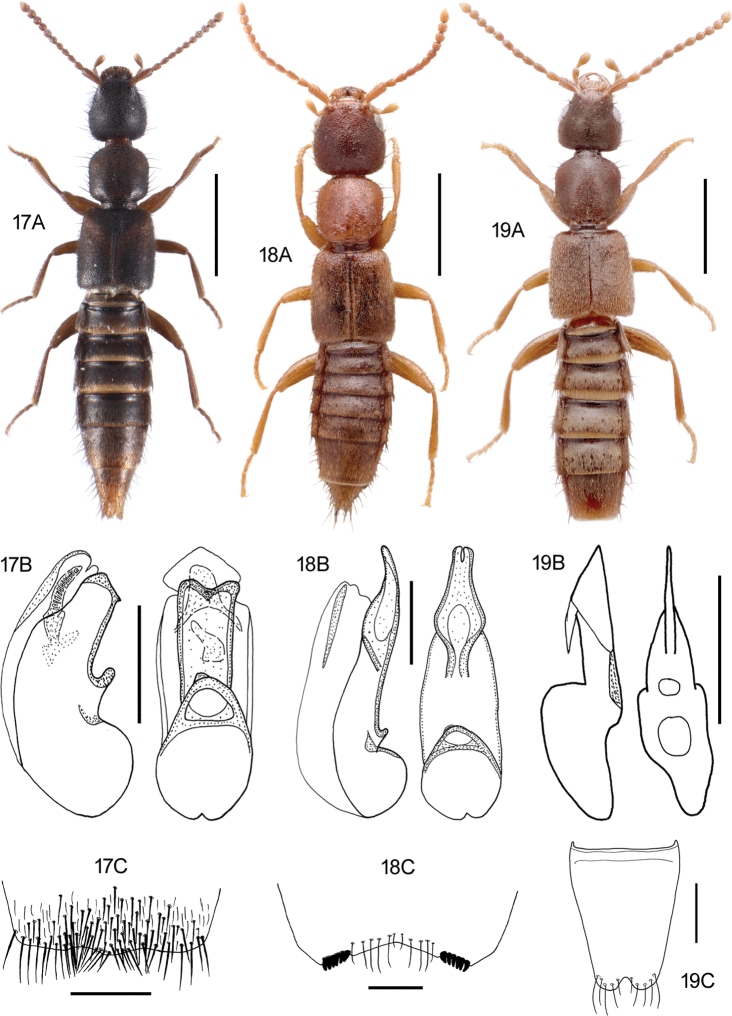
**17***Medonapicalis* (Kraatz) **17A** habitus **17B** aedeagus, ventral and lateral view, V. Assing **17C** male sternite VII, V. Assing **18***Medonripicola* (Kraatz) **18A** habitus **18B** aedeagus, ventral and lateral view, V. Assing **18C** male sternite VII, V. Assing **19***Pseudomedonobscurellus* (Erichson) **19A** habitus **19B** aedeagus, lateral view, V. Assing **19C** male sternite VIII, V. Assing. Scale bars: 1.0 mm (**17A; 18A; 19A**), 0.2 mm (**17B, C; 18B, C; 19B, C**).

Body length: 3.8–4.6 mm. Habitus as in Fig. 17A. Male sternite VII as in Fig. 17C. Aedeagus as in Fig. 17B.

####### Bionomic notes.

This species has been collected in a variety of habitats in Europe, but the breeding habitat requirements are unknown (Assing 2006). Most specimens have been collected in flight (car nets, flight interception traps) (Assing 2006). Specimens have also been collected from stream edges, haystacks, woodland and at light (Assing 2006). This species is less likely to occur in the nests of small mammals than other species of the genus (Assing 2006). The Canadian specimen was collected at a UV light in a mixed forest.

####### Comments.

A single female voucher from Canada was available for study and, while males would normally be necessary to confirm a positive identification in *Medon*, its barcode sequence is identical to German and Austrian specimens of *M.apicalis*. All similar, widespread Palaearctic species that could be confused with *M.apicalis* (*M.ripicola* (Kraatz, 1854), *M.brunneus* (Erichson, 1839), *M.fusculus* (Mannerheim, 1830)) are represented in BOLD and form distinct BIN clusters. The female voucher was also morphologically compared to representatives of all widespread western Palaearctic *Medon* species and was consistent with the variability of body proportions, punctation and color of *M.apicalis*. Four species known from the southwestern Palaearctic are closely related to *M.apicalis* and cannot be reliably distinguished by external characters: *M.perniger* Coiffait, 1978 (Italy and extreme southern parts of France and Switzerland); *M.maronitus* (Saulcy, 1864) (Greece to Turkmenistan); *M.sericellus* Fairmaire, 1860 (North Africa) and *M.beydaghensis* Fagel, 1969 (Turkey) (Assing 2004, 2006). None of these species are currently represented on BOLD. Although one or more of these species might share a BIN with *M.apicalis*, the Ontario specimen has an identical DNA barcode haplotype to specimens from Germany and Austria where *M.apicalis* is the only known representative of this species group. As the Nearctic fauna of *Medon* is unrevised, comparisons with North American species are not yet possible. Recognizing this species in the Nearctic region requires dissected males or DNA barcoding.

###### Medon ripicola

Taxon classificationAnimaliaColeopteraStaphylinidae

(Kraatz, 1854)

3EF4F982-D2C5-5FA9-A2C5-D5CCA59C1044

Figure 18

####### Distribution.

Native to the western Palaearctic region, widespread in Europe and also reported from Algeria, Morocco, Turkey, and Madeira (Schülke and Smetana 2015). Adventive in the Nearctic region (Nova Scotia, Canada).

####### Canadian records.

Nova Scotia: Cape Breton Highlands National Park, 10-May-2013 to 21-May-2013 (1 ex, CBG).

####### Diagnostic information.

Body length: 3.7–4.2 mm. Habitus as in Fig. 18A. Male sternite VII as in Fig. 18C. Aedeagus as in Fig. 18B.

####### Bionomic notes.

This species is rarely collected in the Palaearctic, with its breeding microhabitat unknown (probably in deeper litter or mammal burrows). In Central Europe, specimens have been collected mostly in wetlands (floodplains, ponds), in flood debris, mole nests, and deeper deciduous leaf litter (Assing 2012). Palm (1963) wrote that this species was rarely collected in Scandinavia: once in Sweden under pebbles on the seashore and in Denmark under seaweed. Its occasional but typical appearance near water suggests that heavy rains may flood out the breeding microhabitat and deposit the beetles elsewhere (e.g., flood debris). The collection of *M.ripicola* on northern European seashores suggests a potential mechanism for introduction to the Canadian Maritimes through ocean commerce. The Canadian specimen was collected with a Malaise trap in a riverside forest.

####### Comments.

A single female voucher from Canada was available for study and, while males would normally be necessary to confirm a positive identification in *Medon* by morphology, its barcode sequence clustered within the European material of *M.ripicola* with only two nucleotide sites differing from the nearest European specimen. All similar Palaearctic species that could be confused with *M.ripicola* (*M.apicalis* (Kraatz, 1857), *M.brunneus* (Erichson, 1839), *M.fusculus* (Mannerheim, 1830)) are represented in BOLD in separate BIN clusters. The female voucher was also morphologically compared to representatives of all Palaearctic *Medon* species and was consistent with the body proportions, punctation and color of *M.ripicola*. As the Nearctic fauna of *Medon* is unrevised, useful comparisons with North American species are not yet possible. Recognizing this species in the Nearctic region is reliably accomplished, at present, using dissected males or DNA barcoding.

###### Pseudomedon obscurellus

Taxon classificationAnimaliaColeopteraStaphylinidae

(Erichson, 1840)

3FDD0819-8790-5C9E-8EF2-1C96E343CFE4

Figure 19

####### Distribution.

Native to the western Palaearctic region, widespread in Europe and also reported from Algeria, Morocco, Madeira, Tunisia, Cyprus, and Turkey (Schülke and Smetana 2015). Adventive in the Neotropical region (Chile; Assing 2009) and the Nearctic region (Nova Scotia, Canada).

####### Canadian records.

Nova Scotia: Cape Breton Highlands National Park, 07-Jun-2013 to 24-Jun-2013 (1 ex, CBG).

####### Diagnostic information.

Body length: 3.0–3.4 mm. Habitus as in Fig. 19A. Male sternite VIII as in Fig. 19C. Aedeagus as in Fig. 19B.

####### Bionomic notes.

This species inhabits wetlands and can be collected from rotting organic matter (Assing 2012). The Canadian specimen was collected at the same site and in the same Malaise trap as the *M.ripicola* specimen.

####### Comments.

A single female voucher from Canada was available for study and, while males would normally be necessary to confirm a positive identification in *Pseudomedon*, its barcode sequence is identical to German specimens of *P.obscurellus*. The morphologically similar Palaearctic species *P.obsoletus* forms a separate BIN cluster (BOLD:ABY0636 ). The female voucher from Canada also was consistent with the typical coloration of *P.obscurellus* given by Assing (2012). As the Nearctic fauna of *Pseudomedon* is unrevised, comparisons with North American species are not yet possible. Recognizing this species in the Nearctic region is reliably accomplished, at present, using dissected males or DNA barcoding.

Due to taxonomic confusion until the 1970s, reports of *Pseudomedonobscurellus* and *P.obsoletus* from regions outside of the Palaearctic need re-confirmation (Assing 2009, Klimaszewski et al. 2013). The record of *P.obsoletus* from British Columbia from Hatch (1957) is doubtful and likely refers to *P.obscurellus* as it was described as being partly dark rufous, a color more typically associated with this species (Assing 2012). To our knowledge, this is the first verified record of any Palaearctic *Pseudomedon* species from the Nearctic.

### Scarabaeidae

#### Melolonthinae

##### Melolonthini

###### Phyllophaga implicita

Taxon classificationAnimaliaColeopteraMelolonthidae

(Horn, 1887)

C398D9CD-12F7-5CC9-864A-238BFFBD91F2

####### Distribution.

Native to North America. Occurs across most of the Mississippi River drainage basin in the United States (Luginbill and Painter 1953).

####### Canadian records.

Ontario: Point Pelee National Park, 05-Jun-2008 (1 ex, CBG).

####### Diagnostic information

(partially based on Luginbill and Painter 1953). Body length 14.5–17.0 mm. Dorsal surface pale to dark brown, moderately shiny (not pruinose or iridescent), glabrous, and without scales. Clypeus emarginate. Antennae with nine antennomeres. See Luginbill and Painter (1953) for images of habitus and genitalia.

####### Bionomic notes.

Adults have been observed on numerous plants including *Tilia* L., *Fagus* L., *Betula* L., *Ulmus* L., *Lonicera* L., *Acer* L., *Platanus* L., *Rosa* L., *Juglans* L., *Salix* L. and cultivated legumes (Luginbill and Painter 1953). The Canadian specimen was collected in a mixed forest close to the shore of Lake Erie using an ultraviolet light.

####### Comments.

It is not surprising to find a range extension of this species into Canada considering the widespread distribution in eastern North America and the apparent broad range of host plants. Since only a single specimen was collected in Canada it is difficult to assess how firmly established this species is. There are hundreds of species of *Phyllophaga* with a similar overall appearance; therefore, it is crucial to use the male or female genitalia for morphological species identifications.

##### Clambidae

##### Calyptomerinae

###### Calyptomerus dubius

Taxon classificationAnimaliaColeopteraClambidae

(Marsham, 1802)

5A244D85-B06E-521F-879A-27A5398EEBDC

Figure 20

####### Distribution.

Native to the Palaearctic region, widespread in Central Europe and around the Mediterranean (Endrödy-Younga 1961, Löbl 2006). Adventive in the Afrotropical region (South Africa), the Australian region (Australia) (Endrödy-Younga 1974) and in the Nearctic region (British Columbia, Canada).

####### Canadian records.

British Columbia: Abbotsford, 22-Sep-2014 to 03-Oct-2014 (1 ex, CBG); Vancouver, 22-Sep-2014 to 03-Oct-2014 (1 ex, CBG); Victoria, 03-Sep-2014 to 10-Sep-2014 (1 ex, CBG).

####### Diagnostic information

**Figures 20, 21.**
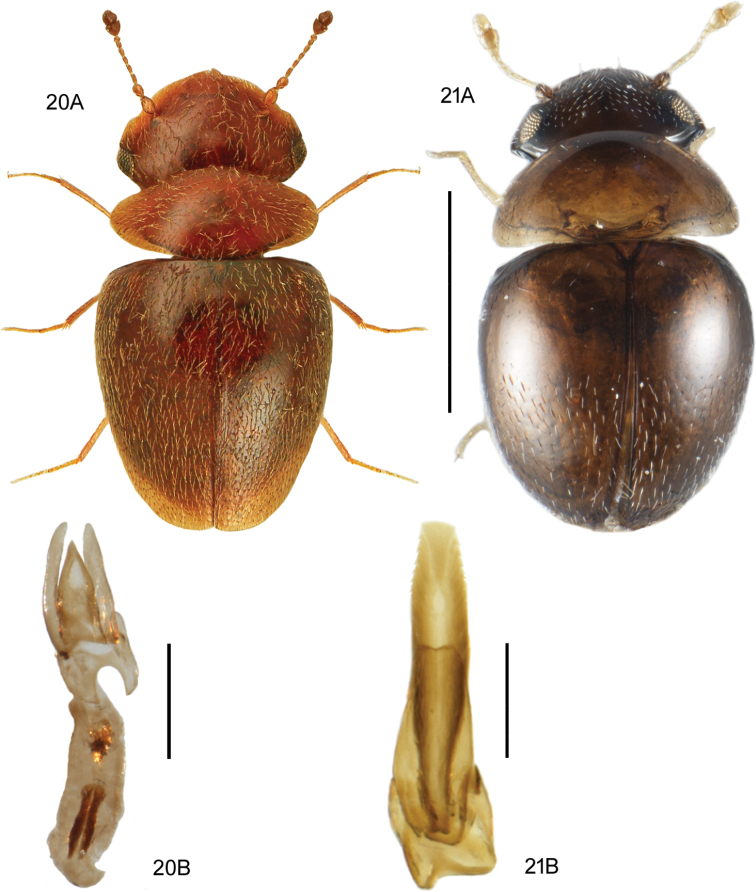
**20***Calyptomerusdubius* (Marsham) **20A** habitus, L. Borowiec **20B** aedeagus, dorsal view **21***Clambussimsoni* Blackburn **21A** habitus **21B** aedeagus, ventral view. Scale bars: 0.5 mm (**20A; 21A**), 0.2 mm (**20B**), 0.1 mm (**21B**).

(based on Endrödy-Younga 1961). Body length 1.1–1.6 mm. Habitus as in Fig. 20A. Red-brown, with the edges of pronotum and elytra paler. The antennal groove forms a continuous and even curve with the side of the frons in front of the eyes. Lateral edges of pronotum bluntly angled. Pubescence on the dorsal surface long and sparse. Elytra angled apicolaterally and truncate at the hind margin. Aedeagus as in Fig. 20B.

####### Bionomic notes.

This species is known from decaying plant material. It has been collected from dead, fungus-infested logs of deciduous trees, leaf litter, composts, moldy hay, etc. (Koch 1989b). The Canadian specimens were collected with Malaise traps in residential areas.

####### Comments.

*Calyptomerusoblongulus* (Mannerheim, 1853) is the only other representative of this genus known from North America. It is larger than *C.dubius* (body length 1.8–2.0 mm), with a rounded angle between the antennal groove and the lateral margin of frons, rounded lateral edges of pronotum, evenly curved (not truncate) elytral hind margins, shorter and denser pubescence on the dorsal surface, and different male genitalia (Endrödy-Younga 1961).

##### Clambinae

###### Clambus simsoni

Taxon classificationAnimaliaColeopteraClambidae

Blackburn, 1902

9BFF3D61-8705-5C55-BF76-598BCA946EC3

Figure 21

####### Distribution.

Native to the Australian region. Described from Australia, where the species is widespread and common (Endrödy-Younga 1990). Also recorded from New Zealand (Johnson 1997). Adventive in the Afrotropical region (South Africa), the Palaearctic region (British Isles and Central Europe; Endrödy-Younga 1990, Johnson 1997, Meybohm 2004, Löbl 2006), and the Nearctic region (British Columbia, Canada).

####### Canadian records.

British Columbia: West Vancouver, 20-Apr-2015 to 08-May-2015 (1 ex, CBG).

####### Diagnostic information

(based on Endrödy-Younga 1990 and Johnson 1997). Body length 1.0–1.2 mm. Habitus as in Fig. 21A. Pale red-brown, with head and anterior part of pronotum darkened. Lateral angles of head narrowly rounded, rectangular, a line drawn between the angles level with the posterior margin of eyes. Dorsal surface without microsculpture. Apical part of elytra with large punctures. Pubescence of elytra relatively long and sparse, individual setae only a little longer than distance between seta-bearing punctures. Aedeagus as in Fig. 21B, penis finely serrate laterally in the apical quarter.

####### Bionomic notes.

This species is known from decaying plant material. It has been collected from heaps of cut grass, heaps of shredded bark, and (in New Zealand) from tree fungi (Johnson 1997). The Canadian specimen was collected with a Malaise trap in a suburban residential area.

####### Comments.

Morphologically, *Clambussimsoni* is most reliably identified by its characteristic male genitalia. The Canadian specimen is a male which shares an identical barcode sequence with a specimen sampled from Germany. In Endrödy-Younga’s (1981) key to the New World species of *Clambus*, *C.simsoni* leads to *C.spangleri* Endrödy-Younga in couplet 14. *Clambussimsoni* is slightly larger (*C.spangleri* is 0.8–0.9 mm according to Endrödy-Younga), and the pubescence on the dorsal surface is sparser than in *C.spangleri*.

### Scirtidae

#### Scirtinae

##### Contacyphon fuscescens

Taxon classificationAnimaliaColeopteraScirtidae

(Klausnitzer, 1976)

780BF9E6-5688-5F1F-829F-06262A07658B

Figure 22

###### Distribution.

Native to North America. Described from New York State (Ithaca) (Klausnitzer 1976).

###### Canadian records.

Yukon Territory: Kluane National Park and Reserve, 15-Jul-2014 to 24-Jul-2014 (1 ex, CBG). British Columbia: Naikoon Provincial Park, 24-Jun-2014 to 03-Jul-2014 (4 exx, CBG); Naikoon Provincial Park, 13-Jul-2014 to 31-Jul-2014 (5 exx, CBG); Naikoon Provincial Park, 08-Aug-2014 to 15-Aug-2014 (4 exx, CBG). Ontario: Puslinch Township, 12-Jun-2010 to 19-Jun-2010 (1 ex, CBG); Short Hills Provincial Park, 26-May-2014 to 09-Jun-2014 (4 exx, CBG); Short Hills Provincial Park, 23-Jun-2014 to 07-Jul-2014 (1 ex, CBG).

###### Diagnostic information

**Figures 22–24.**
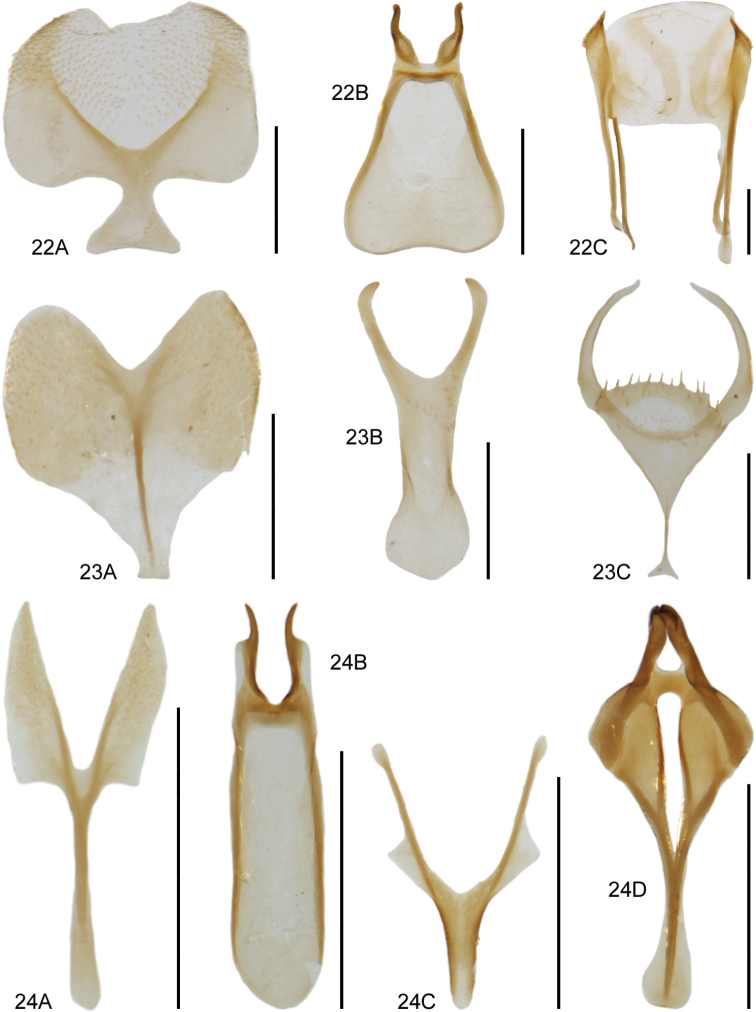
**22***Contacyphonfuscescens* (Klausnitzer) **22A** aedeagus, dorsal plate **22B** aedeagus, ventral plate **22C** male tergite 8 and 9 **23***Contacyphonkongsbergensis* (Munster) **23A** aedeagus, dorsal plate **23B** aedeagus, ventral plate **23C** male sternite 9 and accessory sclerite **24***Contacyphonobscurellus* (Klausnitzer) **24A** aedeagus, dorsal plate **24B** aedeagus, ventral plate **24C** male sternite 9 **24D** male tergite 9. Scale bars: 0.2 mm (**22A–C; 23A–C**), 0.5 mm (**24A–D**).

(based on Klausnitzer 1976). Body length 2.5–3.0 m. Dark brown, basal margin of the pronotum and elytral suture a little paler. Head with granulate punctation, pronotal punctation fine, elytra a little more coarsely punctate than pronotum. Elytra with longitudinal ribs. Male tergite VIII and IX as in Fig. 22C. Aedeagus as in Fig. 22A, B.

###### Bionomic notes.

The Canadian specimens were collected with Malaise traps in wetlands and close to open water in forests and farmland.

###### Comments.

*Contacyphonfuscescens* belongs to the *C.coarctatus* group of species. It is most reliably identified by the male genitalia. The identification of the Canadian specimens is based on dissected male representatives of the BIN.

##### Contacyphon kongsbergensis

Taxon classificationAnimaliaColeopteraScirtidae

(Munster, 1924)

B60342D2-EE04-5D0C-AC64-5E596AF4A37A

Figure 23

###### Distribution.

Holarctic. Recorded from across the Palaearctic region, from Western Europe to the Russian Far East (Klausnitzer 2006). In North America, apparently previously known only from Alaska (Kalsin Bay, Kodiak (Nyholm 1972)). This record was overlooked by Bousquet et al. (2013).

###### Canadian records.

British Columbia: Smithers, 28-Jul-2014 to 05-Aug-2014 (4 exx, CBG). Alberta: Jasper National Park, 02-Aug-2010 to 05-Aug-2010 (1 ex, CBG). Manitoba: Churchill, 05-Aug-2005 (1 ex, CBG); Churchill, 18-Aug-2006 (1 ex, JBWM); Churchill, 21-Jul-2007 (2 exx, CBG); Riding Mountain National Park, 16-Jul-2012 to 23-Jul-2012 (1 ex, CBG).

###### Diagnostic information

(based on Nyholm 1972 and Lohse 1979). Body length 2.7–3.2 mm. Reddish or yellowish brown, head usually darker than pronotum and elytra. Head and pronotum with dense, coarse, granulate punctation. Elytra densely punctate. Male sternite IX and accessory sclerite as in Fig. 23C. Aedeagus as in Fig. 23A, B.

###### Bionomic notes.

This species is known from acidic *Sphagnum* bogs (Nyholm 1972). Most Canadian specimens were collected with Malaise traps; also collected with light traps and by sweep netting.

###### Comments.

*Contacyphonkongsbergensis* is morphologically most reliably identified by its genitalia. The lack of a modern revision of North American *Contacyphon* prevents detailed comparison with related species.

##### Contacyphon obscurellus

Taxon classificationAnimaliaColeopteraScirtidae

(Klausnitzer, 1976)

E5C70F4E-252F-5EED-A4EC-7796AC47C2C7

Figure 24

###### Distribution.

Native to North America. Described from New York State (Adirondack, Long Lake) (Klausnitzer 1976).

###### Canadian records.

Ontario: Georgian Bay Islands National Park, 30-Jul-2013 to 06-Aug-2013 (1 ex, CBG); Guelph, 30-Jun-2018 (1 ex, CBG); Perth, 03-Jul-2014 to 17-Jul-2014 (1 ex, CBG); Warsaw, 04-Jul-2014 to 18-Jul-2014 (1 ex, CBG). New Brunswick: Kouchibouguac National Park, 19-Aug-2009 (1 ex, CBG). Nova Scotia: Cape Breton Highlands National Park, 14-Jul-2013 to 19-Jul-2013 (1 ex, CBG); Cape Breton Highlands National Park, 19-Jul-2013 to 26-Jul-2013 (1 ex, CBG); Cape Breton Highlands National Park, 02-Aug-2013 to 09-Aug-2013 (1 ex, CBG); Kejimkujik National Park, 08-Aug-2013 to 22-Aug-2013 (1 ex, CBG). Newfoundland: Gros Morne National Park, 25-Jun-2013 to 02-Jul-2013 (1 ex, CBG); Gros Morne National Park, 09-Jul-2013 to 16-Jul-2013 (1 ex, CBG); Gros Morne National Park, 09-Jul-2013 to 20-Jul-2013 (9 exx, CBG); Gros Morne National Park, 10-Jul-2013 to 20-Jul-2013 (47 exx, CBG); Gros Morne National Park, 11-Jul-2013 (1 ex, CBG); Gros Morne National Park, 12-Jul-2013 (2 exx, CBG); Gros Morne National Park, 15-Jul-2013 (1 ex, CBG); Gros Morne National Park, 17-Jul-2013 (1 ex, CBG); Gros Morne National Park, 22-Aug-2013 to 27-Aug-2013 (1 ex, CBG); Terra Nova National Park, 24-Jul-2013 to 30-Jul-2013 (3 exx, CBG).

###### Diagnostic information

(based on Klausnitzer 1976). Body length 2.4 mm. Brown, elytral suture pale brown, antennae and legs yellow-brown. Male sternite IX and tergite IX as in Fig. 24C, D. Aedeagus as in Fig. 24A, B.

###### Bionomic notes.

The Canadian specimens were collected in conifer and mixed forests, mainly with Malaise traps.

###### Comments.

*Contacyphonobscurellus* belongs to the *C.variabilis* group of species. It is most reliably identified by its genitalia. The species is split into three closely clustered BINs, which show no obvious morphological differences. The identification of the Canadian specimens is based on dissected male representatives of these BINs.

### Throscidae

#### Aulonothroscus distans

Taxon classificationAnimaliaColeopteraThroscidae

Blanchard, 1917

748BB954-D03A-5A65-9A46-1B97A7A54FB5

##### Distribution.

Native to North America. Blanchard (1917) listed records from Massachusetts, New York and North Carolina.

##### Canadian records.

Ontario: Balsam Lake Provincial Park, 02-Jun-2014 to 16-Jun-2014 (1 ex, CBG); Cambridge, 21-May-2015 to 27-May-2015 (1 ex, CBG); Cambridge, 25-May-2015 to 31-May-2015 (1 ex, CBG); Cambridge, 04-Jun-2015 to 11-Jun-2015 (3 exx, CBG); Cambridge, 18-Jun-2015 to 24-Jun-2015 (1 ex, CBG); Elizabethtown-Kitley, 14-May-2010 to 18-May-2010 (1 ex, CBG); Elizabethtown-Kitley, 28-May-2010 to 30-May-2010 (2 exx, CBG); Georgian Bay Islands National Park, 04-Jun-2013 to 11-Jun-2013 (1 ex, CBG); Georgian Bay Islands National Park, 11-Jun-2013 to 18-Jun-2013 (1 ex, CBG); Georgian Bay Islands National Park, 18-Jun-2013 to 26-Jun-2013 (3 exx, CBG); Georgian Bay Islands National Park, 26-Jun-2013 to 30-Jun-2013 (2 exx, CBG); Georgian Bay Islands National Park, 30-Jul-2013 to 06-Aug-2013 (2 exx, CBG); Georgian Bay Islands National Park, 06-Aug-2013 to 19-Aug-2013 (1 ex, CBG); Georgian Bay Islands National Park, 03-Sep-2013 to 10-Sep-2013 (2 exx, CBG); Guelph, 17-Oct-2013 (1 ex, CBG); Hanover, 30-Jul-2014 to 13-Aug-2014 (1 ex, CBG); Rouge National Urban Park, 04-Jun-2013 to 11-Jun-2013 (1 ex, CBG); Rouge National Urban Park, 11-Jun-2013 to 18-Jun-2013 (6 exx, CBG); Rouge National Urban Park, 02-Jul-2013 to 09-Jul-2013 (1 ex, CBG); Rouge National Urban Park, 23-Jul-2013 to 30-Jul-2013 (1 ex, CBG); Rouge National Urban Park, 20-Aug-2013 to 27-Aug-2013 (1 ex, CBG); Rouge National Urban Park, 26-May-2014 to 03-Jun-2014 (3 exx, CBG); Rouge National Urban Park, 03-Jun-2014 to 10-Jun-2014 (2 exx, CBG); Rouge National Urban Park, 10-Jun-2014 to 17-Jun-2014 (3 exx, CBG); Rouge National Urban Park, 17-Jun-2014 to 24-Jun-2014 (3 exx, CBG); Rouge National Urban Park, 25-May-2014 to 01-Jul-2014 (1 ex, CBG); Rouge National Urban Park, 01-Jul-2014 to 08-Jul-2014 (1 ex, CBG); Rouge National Urban Park, 08-Jul-2014 to 15-Jul-2014 (1 ex, CBG); Rouge National Urban Park, 22-Jul-2014 to 29-Jul-2014 (2 exx, CBG); Rouge National Urban Park, 29-Jul-2014 to 05-Aug-2014 (1 ex, CBG); Rouge National Urban Park, 12-Aug-2014 to 19-Aug-2014 (1 ex, CBG); Rouge National Urban Park, 19-Aug-2014 to 26-Aug-2014 (1 ex, CBG); Rouge National Urban Park, 16-Sep-2014 to 23-Sep-2014 (1 ex, CBG); Rouge National Urban Park, 30-Sep-2014 to 07-Oct-2014 (1 ex, CBG); Thousand Islands National Park, 01-Jun-2012 to 08-Jun-2012 (2 exx, CBG); Thousand Islands National Park, 07-Jul-2012 to 13-Jul-2012 (1 ex, CBG); Thousand Islands National Park, 08-Jun-2012 to 15-Jun-2012 (1 ex, CBG); Thousand Islands National Park, 22-Jun-2012 to 29-Jun-2012 (1 ex, CBG); Thousand Islands National Park, 25-May-2012 to 01-Jun-2012 (1 ex, CBG); Waterloo, 19-Sep-2016 to 30-Sep-2016 (1 ex, CBG).

##### Diagnostic information

(based on Blanchard 1917). Body length 2.6–3.0 mm. Piceous brown. Eyes with a small semicircular emargination. Head with longitudinal carinae separate and slightly diverging on the frons. Prosternal striae entire.

##### Bionomic notes.

The Canadian specimens were collected with Malaise traps, mostly in deciduous or mixed forests.

##### Comments.

The shallow semicircular emargination of the eyes separates this species from the other known North American species of *Aulonothroscus* (Blanchard 1917).

#### Trixagus carinifrons

Taxon classificationAnimaliaColeopteraThroscidae

(Bonvouloir, 1859)

6E2FCF69-0FE5-5CC6-8E9B-FC730F7E7DAC

Figure 25

##### Distribution.

Native to the Palaearctic region. Widespread in Europe, also recorded from the Russian Far East (Leseigneur 2007). Adventive in the Nearctic region (Ontario, Canada).

##### Canadian records.

Ontario: Guelph, 01-Aug-2013 to 08-Aug-2013 (3 exx, CBG); Guelph, 15-Aug-2013 to 22-Aug-2013 (1 ex, CBG); Guelph, 29-Aug-2013 to 05-Sep-2013 (1 ex, CBG); Guelph, 30-Sep-2013 to 04-Oct-2013 (1 ex, CBG); Rouge National Urban Park, 28-May-2013 to 04-Jun-2013 (1 ex, CBG); Rouge National Urban Park, 18-Jun-2013 to 25-Jun-2013 (4 exx, CBG); Rouge National Urban Park, 27-Aug-2013 to 03-Sep-2013 (1 ex, CBG); Rouge National Urban Park, 03-Jun-2014 to 10-Jun-2014 (1 ex, CBG); Rouge National Urban Park, 24-Jun-2014 to 01-Jul-2014 (2 exx, CBG); Rouge National Urban Park, 29-Jul-2014 to 05-Aug-2014 (2 exx, CBG); Rouge National Urban Park, 05-Aug-2014 to 12-Aug-2014 (4 exx, CBG).

##### Diagnostic information

**Figures 25, 26.**
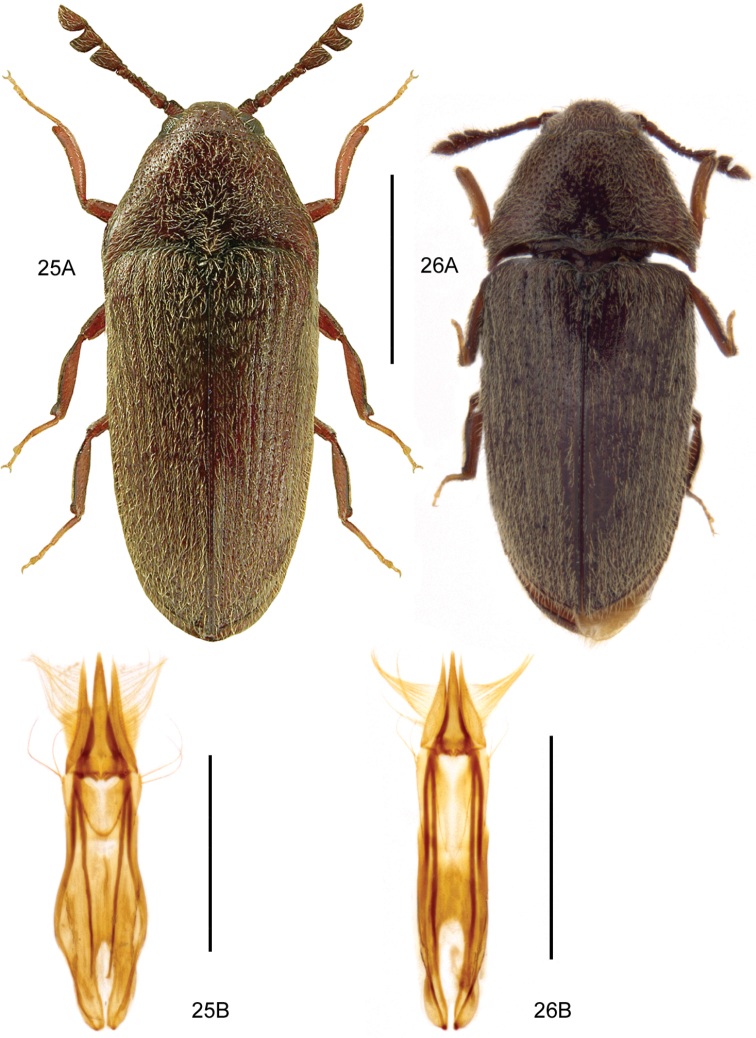
**25***Trixaguscarinifrons* (Bonvouloir) **25A** habitus, L. Borowiec **25B** aedeagus **26***Trixagusmeybohmi* Leseigneur **26A** habitus **26B** aedeagus. Scale bars: 1.0 mm (**25A; 26A**), 0.5 mm (**25B; 26B**).

(based on Leseigneur 1998, 2005). Body length 2.5–3.0 mm. Habitus as in Fig. 25A. Eyes deeply emarginate. Head with longitudinal carinae which converge towards the vertex. Sides of pronotum sinuate in males, slightly or not sinuate in females. Elytral apex in males with a fringe of hairs longer than the lateral pubescence, often hidden under the elytra and not visible without removal of the abdomen. Aedeagus as in Fig. 25B.

##### Bionomic notes.

In Europe, this species is usually found in dry, warm habitats: heaths, forest edges, gravel pits, etc. (Koch 1989b). The Canadian specimens were collected in Malaise traps in a patch of forest and on a lawn between buildings on the University of Guelph campus.

##### Comments.

The genus *Trixagus* includes several overlooked and probably undescribed species in Canada based on DNA barcode data and initial studies of male genitalia of the barcoded material (Bouchard et al. 2017, MP unpublished data). Until the genus is revised, the two Palaearctic *Trixagus* species reported here are most reliably identified using DNA barcodes or male genitalia.

#### Trixagus meybohmi

Taxon classificationAnimaliaColeopteraThroscidae

Leseigneur, 2005

FA4B4D7D-FFBB-5B76-94E5-4E67D9E48FEA

Figure 26

##### Distribution.

Recently described, distribution not yet thoroughly known. Apparently widespread in Europe (Leseigneur 2005, Mertlik and Leseigneur 2007, Silfverberg 2010, Rassi et al. 2015). Adventive in the Nearctic region (British Columbia, Ontario, Quebec, and Nova Scotia, Canada).

##### Canadian records.

British Columbia: North Vancouver, 21-Sep-2015 to 02-Oct-2015 (2 exx, CBG); Vancouver, 20-May-2014 to 26-May-2014 (2 exx, CBG); Vancouver, 03-Jun-2014 to 10-Jun-2014 (1 ex, CBG); Vancouver, 12-Aug-2014 to 19-Aug-2014 (2 exx, CBG); Vancouver, 26-Aug-2014 to 02-Sep-2014 (1 ex, CBG); Vancouver, 22-Sep-2014 to 03-Oct-2014 (1 ex, CBG); West Vancouver, 21-Sep-2015 to 02-Oct-2015 (12 exx, CBG). Ontario: Dundas, 22-Sep-2014 to 03-Oct-2014 (1 ex, CBG); Toronto, 21-Sep-2015 to 02-Oct-2015 (3 exx, CBG). Quebec: Montreal, 19-Sep-2014 to 26-Sep-2014 (1 ex, CBG). Nova Scotia: Point Pleasant Park, 25-May-2013 to 01-Jun-2013 (1 ex, CBG); Point Pleasant Park, 14-Jun-2013 to 22-Jun-2013 (1 ex, CBG); Point Pleasant Park, 06-Jul-2013 to 13-Jul-2013 (2 exx, CBG); Point Pleasant Park, 03-Aug-2013 to 10-Aug-2013 (9 exx, CBG); Point Pleasant Park, 10-Aug-2013 to 17-Aug-2013 (7 exx, CBG); Point Pleasant Park, 17-Aug-2013 to 24-Aug-2013 (6 exx, CBG); Point Pleasant Park, 24-Aug-2013 to 31-Aug-2013 (4 exx, CBG); Point Pleasant Park, 31-Aug-2013 to 07-Sep-2013 (1 ex, CBG); Point Pleasant Park, 07-Sep-2013 to 14-Sep-2013 (2 exx, CBG).

##### Diagnostic information

(based on Leseigneur 2005). Body length 2.4–3.2 mm. Habitus as in Fig. 26A. Eyes deeply emarginate. Head with slender longitudinal carinae, which are parallel or slightly converging towards the vertex. Sides of pronotum not or only slightly sinuate, slightly angulate in males, rounded or weakly angulate in females. Elytral apex in males with a fringe of long setae (setae as long as antennomere 11), which can be tucked under the elytra and may not be visible without removal of the abdomen. Aedeagus as in Fig. 26B.

##### Bionomic notes.

The Canadian specimens were collected with Malaise traps in city parks and suburban residential areas.

##### Comments.

Until the North American species of *Trixagus* are revised, *T.meybohmi* is most reliably identified using DNA barcodes or male genitalia.

### Elateridae

#### Dendrometrinae

##### Prosternini

###### Pseudanostirus tigrinus

Taxon classificationAnimaliaColeopteraElateridae

(Fall, 1901) comb. nov.

8449BD30-12E0-5B31-847C-A405BDFAC43D

BOLD: ACU2924

####### Distribution.

Native to North America. Previously known only from the United States, where the species is known from areas near Lake Tahoe in California (Fall 1901). CNC has additional specimens collected in northern Oregon and near Lake Tahoe in Nevada.

####### Canadian record

(DNA barcoded specimen). British Columbia: Gulf Islands National Park Reserve, 30-May-2014 to 08-Jun-2014 (1 ex, CBG).

####### Additional Canadian record.

British Columbia: Parksville, 11-Apr-2018 (1 ex, CNC).

####### Diagnostic information

(based on Brown 1936). Body length 9.0–11.2 mm. Antennae with antennomeres 3 and 5 of equal length. Pronotum black, with pubescence pale except for two to circular patches of dark setae on each side. Elytra red-brown with pale setae, with band of darker setae surrounding scutellar shield and three angulate transverse bands of dark setae extending from suture to epipleura.

####### Bionomic notes.

*Pseudanostirustigrinus* has been collected by beating *Pseudotsuga* Carrière on a grassy hillside with *Quercusgarryana* Douglas ex Hook. trees. Other specimens have been collected in Malaise and funnel traps also in semi-open woodland with *Arbutus* L. and *Pseudotsuga* trees in warm-summer Mediterranean climate areas. The barcoded specimen was collected with a Malaise trap in a coastal mixed forest.

####### Comments.

This species was described as *Corymbitestigrinus* Fall, 1901. Brown (1936) placed this species in *Ludius* Berthold, 1827 as *Ludiustigrinus* (Fall, 1901), part of the *L.triundulatus* species group. Lane (1948) found that *Ludius* Eschscholtz, 1829 was a synonym of *Elater* Linnaeus, 1758 and transferred all North American *Ludius* to *Ctenicera* Latreille, 1829. Johnson (2002) indicated that all species of Brown’s *triundulatus* group should be transferred to *Pseudanostirus* but did not formally present any new combinations. This combination has not been used previously in the scientific literature. Therefore the resulting combination *Pseudanostirustigrinus* (Fall, 1901) is used here for the first time.

*Pseudanostirustigrinus* is similar to *P.nebraskensis* (Bland, 1863). Its independent placement in a separate BIN cluster supports the validity of *P.tigrinus*.

### Cantharidae

#### Cantharinae

##### Podabrini

###### Dichelotarsus lapponicus

Taxon classificationAnimaliaColeopteraCantharidae

(Gyllenhal, 1810)

1BEB17CF-AC90-5159-99FA-58D594AA6A5E

Figure 27

####### Distribution.

Previously only recorded from the Palaearctic region. A northern species, found in Norway, Sweden, and Finland, and across the northern Palaearctic to the Russian Far East and Japan (Hokkaido) (Kazantsev and Brancucci 2007; Silfverberg 2010; Rassi et al. 2015). Probably Holarctic and previously overlooked in North America.

####### Canadian records.

Yukon Territory: Ivvavik National Park, 17-Jun-2014 to 23-Jun-2014 (3 exx CBG); Ivvavik National Park, 23-Jun-2014 to 29-Jun-2014 (16 exx, CBG).

####### Diagnostic information

**Figures 27, 28.**
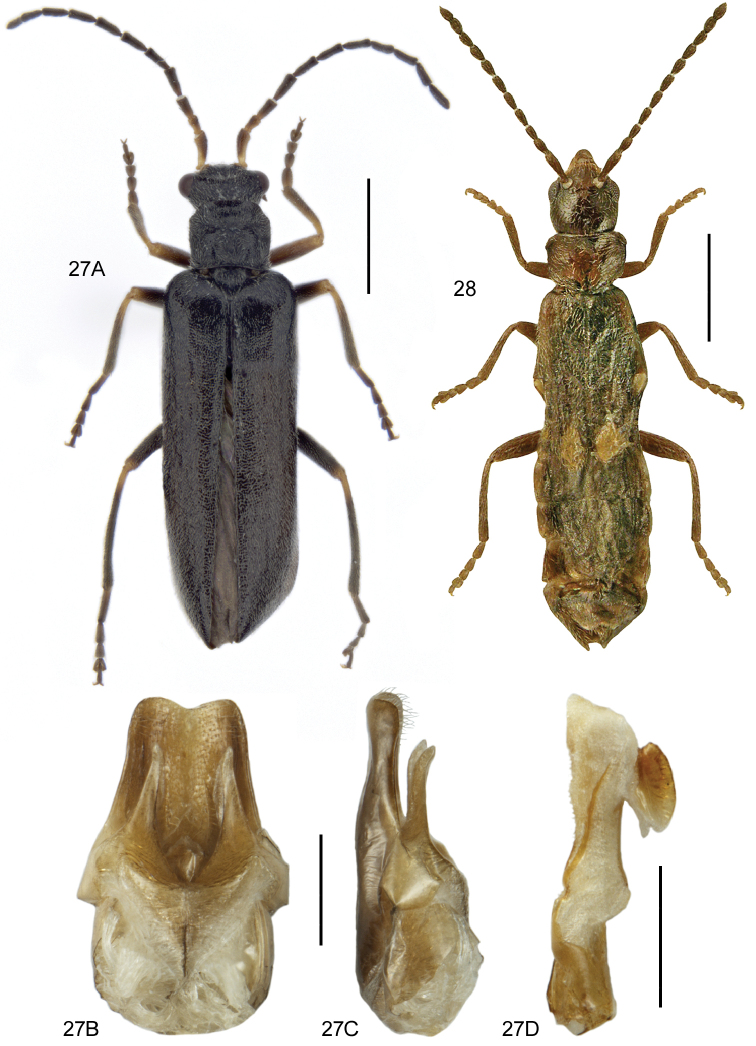
**27***Dichelotarsuslapponicus* (Gyllenhal) **27A** habitus **27B** aedeagus, ventral view (everted endophallus removed) **27C** aedeagus, lateral view (everted endophallus removed) **27D** endophallus, lateral view **28***Malthodespumilus* (Brébisson), habitus, L. Borowiec. Scale bars: 2.0 mm (**27A**), 0.5 mm (**27B–D**), 0.3 mm (**28**).

(based on Kazantsev 1998). Body length 7–10 mm. Habitus as in Fig. 27A. Dark brown to black, basal antennomeres, mandibles and usually clypeus (at least at the margins) yellow. Legs variably yellow, usually at least the profemora yellow. Third antennomere in males ca. 1.5 times as long as the second. Pronotum as wide as long or slightly wider than long, with sides concave before acute hind angles. All tarsal claws in both sexes with a broad, blunt basal tooth, no claws deeply cleft. Aedeagus as in Fig. 27B−D, with dorsal plate with apical notch.

####### Bionomic notes.

In Northern Finland, this species is found both above and below the treeline, usually in wetlands (MP, pers. obs.). The Canadian specimens were collected with a Malaise trap on tundra close to the Arctic treeline.

####### Comments.

The remote arctic collecting locality suggests that this species is more likely to be Holarctic than adventive from the Palaearctic region. The legs and basal antennomeres of the Canadian specimens are darker and the body length is slightly smaller compared to North European material we examined (including the DNA barcoded Finnish specimens with which the Canadian specimens share the BIN cluster). The male genitalia and shape of the pronotum show no differences between the European and Canadian specimens. Based on the identification keys, descriptions and figures by Fall (1927) and Fender (1961), *D.lapponicus* closely resembles *D.piniphilus* (Eschscholtz, 1830). The tarsal claw formula is the same and the shape of the pronotum is very similar in both species. The dorsal plate of the aedeagus has an apical notch in *D.lapponicus* (as in Fig. 27B), whereas in *D.piniphilus* it is apically truncate or subtruncate. The clypeus of *D.lapponicus* is usually yellow at least at the margins. The yellow color is more extensive in males in the material we have seen, and only faint red-brown spots are visible on the clypeus of some female specimens. The clypeus is black in *D.piniphilus*. Pelletier and Hébert (2014) state that *D.lapponicus* resembles *D.perplexus* (W.J. Brown, 1940), which is known from across boreal and arctic Canada, but *D.perplexus* is smaller (body length 5.0–6.5 mm) and has a different tarsal claw formula.

#### Malthininae

##### Malthodini

###### Malthodes pumilus

Taxon classificationAnimaliaColeopteraCantharidae

(Brébisson, 1835)

96D1DDBC-E7D1-5226-9ED3-C21A7C7B6221

Figure 28

####### Distribution.

Native to the Palaearctic region. Widespread in Europe, also recorded from Iran and Turkey (Kazantsev and Brancucci 2007). Adventive in the Nearctic region (British Columbia, Alberta, Saskatchewan, Manitoba, Ontario, Quebec, New Brunswick, Nova Scotia, Prince Edward Island, and Newfoundland, Canada).

####### Canadian records.

British Columbia: Mount Revelstoke National Park, 19-Jun-2014 to 26-Jun-2014 (15 exx, CBG); Mount Revelstoke National Park, 04-Jul-2014 to 09-Jul-2014 (22 exx, CBG); New Afton Mine, 06-Jun-2013 to 13-Jun-2013 (4 exx, CBG); New Afton Mine, 20-Jun-2013 to 27-Jun-2013 (1 ex, CBG); Vancouver, 03-Jun-2014 to 10-Jun-2014 (4 exx, CBG); Vancouver, 17-Jun-2014 to 24-Jun-2014 (1 ex, CBG); Yoho National Park, 25-Jun-2014 to 07-Jul-2014 (1 ex, CBG). Alberta: Banff National Park, 17-Jun-2012 (1 ex, CBG); Banff National Park, 19-Jun-2012 (1 ex, CBG); Banff National Park, 15-Jun-2012 to 20-Jun-2012 (1 ex, CBG); Elk Island National Park, 22-Jun-2012 to 29-Jun-2012 (1 ex, CBG); Elk Island National Park, 01-Jul-2012 (1 ex, CBG); Elk Island National Park, 02-Jul-2012 (1 ex, CBG); Elk Island National Park, 29-Jun-2012 to 03-Jul-2012 (17 exx, CBG); Elk Island National Park, 06-Jul-2012 to 13-Jul-2012 (8 exx, CBG); Jasper National Park, 04-Jul-2012 to 11-Jul-2012 (1 ex, CBG); Waterton Lakes National Park, 27-Jun-2012 to 04-Jul-2012 (3 exx, CBG); Waterton Lakes National Park, 10-Jul-2012 to 17-Jul-2012 (4 exx, CBG); Waterton Lakes National Park, 17-Jul-2012 to 24-Jul-2012 (3 exx, CBG). Saskatchewan: Grasslands National Park, 29-Jun-2014 to 08-Jul-2014 (3 exx, CBG). Manitoba: Riding Mountain National Park, 02-Jul-2012 to 09-Jul-2012 (10 exx, CBG). Ontario: Algonquin Provincial Park, 01-Jul-2014 to 15-Jul-2014 (2 exx, CBG); Bayview Escarpment Provincial Park, 29-May-2014 to 12-Jun-2014 (6 exx, CBG); Bayview Escarpment Provincial Park, 26-Jun-2014 to 10-Jul-2014 (1 ex, CBG); Bruce Peninsula National Park, 21-Jun-2012 to 28-Jun-2012 (2 exx, CBG); Bruce Peninsula National Park, 28-Jun-2012 to 07-Jul-2012 (3 exx, CBG); Bruce Peninsula National Park, 05-Jul-2012 to 12-Jul-2012 (3 exx, CBG); Bruce Peninsula National Park, 12-Jul-2012 to 18-Jul-2012 (2 exx, CBG); Elizabethtown-Kitley, 02-Jun-2010 to 04-Jun-2010 (1 ex, CBG); Elizabethtown-Kitley, 04-Jun-2010 to 06-Jun-2010 (1 ex, CBG); Elizabethtown-Kitley, 06-Jun-2010 to 08-Jun-2010 (1 ex, CBG); Elizabethtown-Kitley, 12-Jun-2010 to 14-Jun-2010 (1 ex, CBG); Elizabethtown-Kitley, 15-Jun-2011 to 20-Jun-2011 (2 exx, CBG); Frontenac Provincial Park, 05-Jun-2014 to 19-Jun-2014 (3 exx, CBG); Georgian Bay Islands National Park, 04-Jun-2013 to 11-Jun-2013 (1 ex, CBG); Georgian Bay Islands National Park, 11-Jun-2013 to 18-Jun-2013 (7 exx, CBG); Georgian Bay Islands National Park, 18-Jun-2013 to 26-Jun-2013 (6 exx, CBG); Guelph, 19-Jun-2015 to 26-Jun-2015 (1 ex, CBG); Hartington, 30-May-2017 (1 ex, CBG); Hartington, 13-Jun-2017 (2 exx, CBG); Hartington, 28-Jun-2017 (1 ex, CBG); Inverhuron Provincial Park, 25-Jun-2014 to 09-Jul-2014 (4 exx, CBG); Lion`s Head Provincial Park, 26-Jun-2014 to 10-Jul-2014 (1 ex, CBG); Lower Madawaska River Provincial Park, 02-Jul-2014 to 16-Jul-2014 (2 exx, CBG); Murphy`s Point Provincial Park, 05-Jun-2014 to 19-Jun-2014 (1 ex, CBG); Peterborough, 24-May-2015 to 30-May-2015 (1 ex, CBG); Peterborough, 07-Jun-2015 to 13-Jun-2015 (2 exx, CBG); Pinery Provincial Park, 25-Jun-2014 to 09-Jul-2014 (1 ex, CBG); Puslinch Township, 05-Jun-2010 to 12-Jun-2010 (1 ex, CBG); Rouge National Urban Park, 03-Jun-2013 to 09-Jun-2013 (2 exx, CBG); Sandbanks Provincial Park, 05-Jun-2014 to 19-Jun-2014 (1 ex, CBG); Short Hills Provincial Park, 26-May-2014 to 09-Jun-2014 (1 ex, CBG); Sudbury, 08-Jun-2010 (1 ex, CBG); Thousand Islands National Park, 01-Jun-2012 to 08-Jun-2012 (1 ex, CBG); Thousand Islands National Park, 08-Jun-2012 to 15-Jun-2012 (5 exx, CBG); Thousand Islands National Park, 15-Jun-2012 to 22-Jun-2012 (3 exx, CBG). Quebec: Forillon National Park, 05-Jul-2013 to 15-Jul-2013 (4 exx, CBG); Forillon National Park, 15-Jul-2013 to 22-Jul-2013 (6 exx, CBG); Forillon National Park, 22-Jul-2013 to 30-Jul-2013 (4 exx, CBG); Forillon National Park, 28-Jun-2013 to 05-Jul-2013 (1 ex, CBG); Mingan Archipelago National Park Reserve, 02-Jul-2013 to 09-Jul-2013 (1 ex, CBG). New Brunswick: Fundy National Park, 18-Jun-2013 to 25-Jun-2013 (1 ex, CBG); Fundy National Park, 02-Jul-2013 to 09-Jul-2013 (22 exx, CBG); Fundy National Park, 09-Jul-2013 to 16-Jul-2013 (33 exx, CBG); Fundy National Park, 16-Jul-2013 to 23-Jul-2013 (8 exx, CBG). Nova Scotia: Cape Breton Highlands National Park, 14-Jul-2013 to 19-Jul-2013 (1 ex, CBG); Cape Breton Highlands National Park, 19-Jul-2013 to 26-Jul-2013 (1 ex, CBG); Kejimkujik National Park, 13-Jun-2013 to 20-Jun-2013 (1 ex, CBG); Kejimkujik National Park, 27-Jun-2013 to 05-Jul-2013 (1 ex, CBG); Sable Island National Park Reserve, 01-Jul-2014 to 14-Jul-2014 (3 exx, CBG). Prince Edward Island: Prince Edward Island National Park, 03-Jul-2013 to 10-Jul-2013 (1 ex, CBG). Newfoundland: Gros Morne National Park, 12-Jul-2013 (1 ex, CBG); Gros Morne National Park, 09-Jul-2013 to 16-Jul-2013 (109 exx, CBG); Gros Morne National Park, 09-Jul-2013 to 20-Jul-2013 (24 exx, CBG); Gros Morne National Park, 10-Jul-2013 to 20-Jul-2013 (6 exx, CBG); Gros Morne National Park, 23-Jul-2013 to 30-Jul-2013 (25 exx, CBG); Gros Morne National Park, 06-Aug-2013 to 13-Aug-2013 (2 exx, CBG); Terra Nova National Park, 25-Jun-2013 to 02-Jul-2013 (4 exx, CBG); Terra Nova National Park, 09-Jul-2013 to 16-Jul-2013 (37 exx, CBG); Terra Nova National Park, 24-Jul-2013 to 30-Jul-2013 (3 exx, CBG).

####### Diagnostic information

(based on Wittmer 1979). Body length 1.3–1.5 mm. Habitus as in Fig. 28. Unicolorous dark brown to black, tarsi slightly paler. Mandibles with a finely serrate additional tooth on the inner surface. Male with the last ventrite long and narrow, deeply emarginate (almost to the middle), last visible tergite likewise deeply emarginate.

####### Bionomic notes.

In Europe, this eurytopic species is usually found in dry, warm habitats such as exposed forest edges, dry meadows etc. (Koch 1989b). The larvae probably live in dead wood as predators (Koch 1989b). The Canadian specimens were collected in a variety of habitats, mainly forests, and mainly in Malaise traps.

####### Comments.

The minute size distinguishes this species from all other Canadian species of *Malthodes* except for *M.parvulus* (LeConte, 1851) (Fender 1951, Pelletier and Hébert 2014). *Malthodesparvulus* is paler, with the first two antennomeres, pronotum, elytra and legs yellow (Pelletier and Hébert 2014). The structure of the terminal abdominal segments in males is also quite different between these species (see Fender (1951) or Pelletier and Hébert (2014) for figures of *M.parvulus*). However, all morphologically examined Canadian specimens of *M.pumilus* were females. It is probably a mainly parthenogenetic species, as males are rare in Europe as well (Wittmer 1979). The genitalia and modifications of the terminal abdominal segments of males are often crucial for morphological identification of *Malthodes* species (Fender 1951; Wittmer 1970). The extreme scarcity of males for morphological diagnosis combined with the lack of recent taxonomic work on the genus in North America probably explains why *M.pumilus* has remained undetected despite being apparently widespread and common across Canada.

### Dermestidae

#### Attageninae

##### Attagenini

###### Attagenus smirnovi

Taxon classificationAnimaliaColeopteraDermestidae

Zhantiev, 1973)

5D6A0B62-20A6-5C44-A285-5805E87FBB66

Figure 29

####### Distribution.

Native to the Afrotropical region. Adventive in the Palaearctic region, first recorded from Europe in the 1960s (misidentified under various species names), distribution expanded in recent decades (Stengaard Hansen et al. 2012). Adventive in the Nearctic region (Ontario, Canada).

####### Canadian records.

Ontario: Toronto, 19-Jul-2016 (3 exx, CBG).

####### Diagnostic information

**Figure 29.**
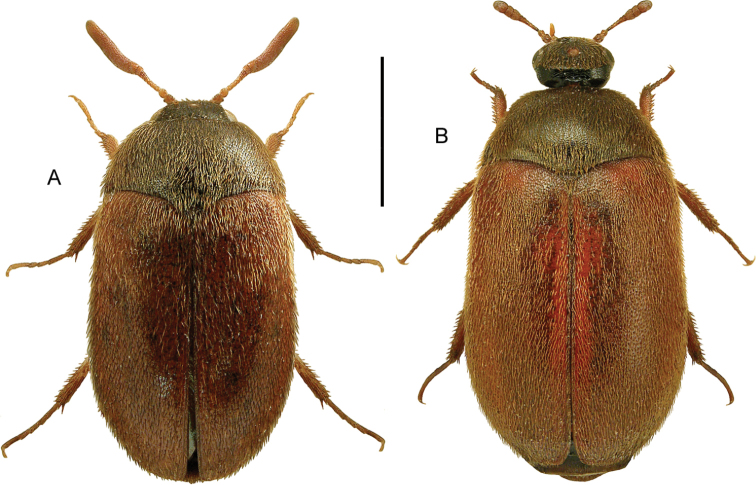
*Attagenussmirnovi* Zhantiev, habitus, L. Borowiec **A** male **B** female. Scale bar: 1.0 mm.

(based on Peacock 1979, Halstead 1981, and Kalik 1992). Body length 2.3–4.0 mm. Habitus as in Fig. 29A, B. Dark brown to black with yellow pubescence, elytra red-brown in males, usually paler yellow-brown in females. Antennae and legs red-brown or yellow-brown. Male antennomere 11 slightly curved at the base, ca. four times longer than wide and ca. four times as long as the combined length of antennomeres 9 and 10. Female antennal club elongate, last antennomere not modified, ovoid. Propleurotrochantin exposed. Anterior ventral carina of mesofemur prominent and sharp, posterior carina weakly developed. Metacoxa reaching metepimeron.

####### Bionomic notes.

This species is recorded from the nests of the Little swift (*Apusaffinis* (J.E. Gray, 1830)) in Kenya (Peacock 1979). It is an indoor pest of various materials of animal origin in Europe (Stengaard Hansen et al. 2012). The Canadian specimens (two larvae and one adult female) were collected in an apartment in Toronto.

####### Comments.

Vernacularly known as the brown carpet beetle. The coloration makes this species quite distinctive among *Attagenus* species recorded from Canada. Presence of adults and larvae in a home suggest establishment in Canada. It is unknown how large or viable Canadian populations of this species are.

### Ptinidae

#### Dorcatominae

##### Petalium incisum

Taxon classificationAnimaliaColeopteraAnobiidae

Ford, 1973

A70F565F-D5F7-5D81-B4A0-BF5DFD46218B

###### Distribution.

Native to North America. Widespread in eastern United States (White 1982; Arango and Young 2012).

###### Canadian records.

Ontario: Burlington, 07-Aug-2014 to 20-Aug-2014 (1 ex, CBG); Cambridge, 15-Jul-2017 (4 exx, CBG); Guelph, 30-Jul-2017 (1 ex, CBG).

###### Diagnostic information.

See Ford (1973) and Arango and Young (2012).

###### Bionomic notes.

Ford (1973) reared this species from *Rhustoxicodendron* L. and *Robiniapseudoacacia* L. (probably from dead dry wood). One Canadian specimen was caught with a Malaise trap; the rest were collected by beating vegetation in deciduous and mixed forests.

### Erotylidae

#### Cryptophilinae

##### Cryptophilini

###### Cryptophilus obliteratus

Taxon classificationAnimaliaColeopteraErotylidae

Reitter, 1874

13E3B3FA-34F0-5820-A4C7-202136FE9F56

####### Distribution.

Native to the Palaearctic region (Asia). Adventive in the western Palaearctic region, recorded in Europe at least from Austria, Denmark, Germany, and France (Denux and Zagatti 2010). Adventive in the Nearctic region (Arkansas, Massachusetts, Maryland, Minnesota, and Wisconsin, United States (Esser 2017), and Ontario, Canada).

####### Canadian records.

Ontario: Ailsa Craig, 22-Apr-2013 to 03-May-2013 (1 ex, CBG); Dunnville, 22-Apr-2013 to 03-May-2013 (1 ex, CBG); London, 22-Apr-2013 to 03-May-2013 (2 exx, CBG); Orillia, 20-Apr-2015 to 08-May-2015 (1 ex, CBG); Peterborough, 05-Jul-2015 to 11-Jul-2015 (1 ex, CBG); Port Rowan, 22-Apr-2013 to 03-May-2013 (2 exx, CBG); Scarborough, 20-Apr-2015 to 08-May-2015 (1 ex, CBG); Whitby, 22-Apr-2013 to 03-May-2013 (5 exx, CBG).

####### Diagnostic information

(based on Esser 2016). Body length ca. 2.5 mm. Color red-brown with more-or-less extensive black markings on the elytra, usually a transverse black band or black lateral spots. Lateral margins of pronotum rounded, more abruptly narrowed basally, concave just in front of the sharp, approximately right-angled hind angles. Meso- and metatibiae bent inwards in males. Parameres relatively shorter than in the other two species recorded from North America. For habitus and genital figures, see Esser (2016) and Jens Esser’s homepage: http://cryptophagidae.de/Cryptophilus-Erotylidae-pleasing-fungus-beetles/

####### Bionomic notes.

This species has been collected from heaps of compost and garden waste in Germany (Franzen 1991). The Canadian specimens were collected with Malaise traps, mainly from suburban residential areas.

####### Comments.

Esser (2017) reported *Cryptophilusobliteratus* from the United States and synonymized *Cryptophilusseriatus* Casey, 1924 (described from Massachusetts) with it. This species has obviously been present in North America for a long time, and Canadian records older than those reported here may well be found in collections.

###### Cryptophilus propinquus

Taxon classificationAnimaliaColeopteraErotylidae

Reitter, 1874

4193C61E-4D7A-57F4-8141-42B84A29BB52

####### Distribution.

This species was confused with *Cryptophilusangustus* (Rosenhauer, 1856) under the name *Cryptophilusinteger* (Heer, 1841) until recently. Therefore, its distribution is not yet very well known. *Cryptophiluspropinquus* was described from Japan, and has been recorded at least from Germany, India, Italy, Turkey, and the United States (Esser 2016, 2017). Adventive in the Nearctic region (Maryland, Minnesota, Mississippi, and Wisconsin, United States (Esser 2017), and British Columbia and Ontario, Canada).

####### Canadian records.

British Columbia: Victoria, 25-Jun-2014 to 02-Jul-2014 (1 ex, CBG); Victoria, 23-Jul-2014 to 30-Jul-2014 (2 exx, CBG). Ontario: Cambridge, 04-Jun-2015 to 11-Jun-2015 (1 ex, CBG); Guelph, 15-Jul-2010 to 01-Aug-2010 (1 ex, CBG); Guelph, 17-Sep-2017 (2 exx, CBG); Rouge National Urban Park, 15-Sep-2013 (1 ex, CBG).

####### Diagnostic information

(based on Esser 2016). Body length ca. 2 mm. Red-brown without black markings on the elytra. Sides of pronotum evenly rounded in dorsal view, no concavity before blunt hind angles. Meso- and metatibiae dilated distally, sometimes with a blunt angle on the dorsal edge in males, but not bent ventrad in either sex. Parameres relatively longer than in *C.obliteratus*, but shorter than in *C.angustus*. For habitus and genital figures, see Esser (2016) and Jens Esser’s homepage: http://cryptophagidae.de/Cryptophilus-Erotylidae-pleasing-fungus-beetles/

####### Bionomic notes.

This species is found in decaying plant material, e.g., in compost heaps (Koch 1989b, Ruta et al. 2011). The DNA barcoded Canadian specimens were collected in Malaise traps in suburban residential areas, in pitfall traps in forest fragments, and by sifting compost heaps.

####### Comments.

*Cryptophilusinteger* (Heer, 1841) is listed as occurring in Canada by (Bousquet et al. 2013), but Esser (2016, 2017) discovered that the name is not valid and refers specimens identified as *C.integer* to two different species: *C.propinquus* Reitter, 1874 and *C.angustus* (Rosenhauer, 1856) (= *C.simplex* (Wollaston, 1857)). Esser (2017) reported that both of these species occur in the Nearctic region but listed no records from Canada for either species. We found only *C.propinquus* among the DNA barcoded Canadian specimens, but not *C.angustus*.

### Cryptophagidae

#### Cryptophaginae

##### Cryptophagini

###### Henoticus mycetoecus

Taxon classificationAnimaliaColeopteraCryptophagidae

(Park, 1929)

38A84043-F2D1-5707-BCC5-AA90DCC12423

####### Distribution.

Native to North America. Described from Illinois (Park 1929), also recorded from Iowa (Downie and Arnett 1996).

####### Canadian records.

Ontario: Rouge National Urban Park, 18-Jun-2013 to 25-Jun-2013 (1 ex, CBG).

####### Diagnostic information

(based on Park (1929)). Body length 1.8–2.0 mm. More or less uniformly red-brown, with legs, antennae, and medial part of elytra paler. Lateral margins of pronotum serrate, sublateral carinae absent. Posterior of pronotum with two deep foveae connected by a distinct basal groove.

####### Bionomic notes.

Park (1929) collected the type specimens from decaying fruiting bodies of the polypore fungus *Climacodonseptentrionalis* (Fr.) P. Karst. in a sugar maple forest. The Canadian specimen was caught with a Malaise trap in a patch of forest.

### Phalacridae

#### Phalacrinae

##### Acylomus ergoti

Taxon classificationAnimaliaColeopteraPhalacridae

Casey, 1890

D503A22B-134D-50E1-A82D-1474FD4DE15E

Figure 30

###### Distribution.

United States (Gimmel 2013). *Tinodemusgrouvellei* Guillebeau, 1894, synonymized under *A.ergoti* by Gimmel (2013), was described from Michigan.

###### Canadian records.

Ontario: Brantford, 19-Sep-2016 to 30-Sep-2016 (1 ex, CBG); Breslau, 22-Apr-2013 to 03-May-2013 (1 ex, CBG); Cambridge, 07-May-2015 to 14-May-2015 (3 exx, CBG); Cambridge, 14-May-2015 to 21-May-2015 (1 ex, CBG); Cambridge, 25-May-2015 to 31-May-2015 (1 ex, CBG); Elizabethtown-Kitley, 30-Apr-2010 to 02-May-2010 (1 ex, CBG); Elizabethtown-Kitley, 09-May-2010 to 14-May-2010 (1 ex, CBG); Elizabethtown-Kitley, 14-May-2010 to 18-May-2010 (1 ex, CBG); Elizabethtown-Kitley, 30-May-2010 to 02-Jun-2010 (1 ex, CBG); Embro, 22-Apr-2013 to 03-May-2013 (1 ex, CBG); Georgian Bay Islands National Park, 06-May-2013 to 12-May-2013 (1 ex, CBG); Georgian Bay Islands National Park, 12-May-2013 to 23-May-2013 (9 exx, CBG); Georgian Bay Islands National Park, 30-Jul-2013 to 06-Aug-2013 (1 ex, CBG); Hagersville, 23-Sep-2013 to 04-Oct-2013 (1 ex, CBG); Hartington, 28-Jun-2017 (1 ex, CBG); London, 22-Apr-2013 to 03-May-2013 (1 ex, CBG); Mississauga, 21-Sep-2015 to 02-Oct-2015 (1 ex, CBG); Orangeville, 22-Sep-2014 to 03-Oct-2014 (1 ex, CBG); Peterborough, 31-May-2015 to 06-Jun-2015 (1 ex, CBG); Peterborough, 29-Jun-2015 to 02-Jul-2015 (1 ex, CBG); Point Pelee National Park, 02-May-2012 to 09-May-2012 (8 exx, CBG); Point Pelee National Park, 16-May-2012 to 23-May-2012 (10 exx, CBG); Point Pelee National Park, 27-Jun-2012 to 04-Jul-2012 (1 ex, CBG); Point Pelee National Park, 05-Sep-2012 to 12-Sep-2012 (1 ex, CBG); Rouge National Urban Park, 07-Jun-2013 (2 exx, CBG); Rouge National Urban Park, 03-Jun-2013 to 09-Jun-2013 (10 exx, CBG); Rouge National Urban Park, 17-Jun-2014 to 24-Jun-2014 (1 ex, CBG); Rouge National Urban Park, 08-Jul-2014 to 15-Jul-2014 (1 ex, CBG); Williamstown, 22-Sep-2014 to 03-Oct-2014 (2 exx, CBG). Quebec: Montreal, 24-Jul-2014 to 02-Aug-2014 (1 ex, CBG); Montreal, 19-Sep-2014 to 26-Sep-2014 (1 ex, CBG). New Brunswick: Springfield, 21-Sep-2015 to 02-Oct-2015 (1 ex, CBG).

###### Diagnostic information

**Figures 30, 31.**
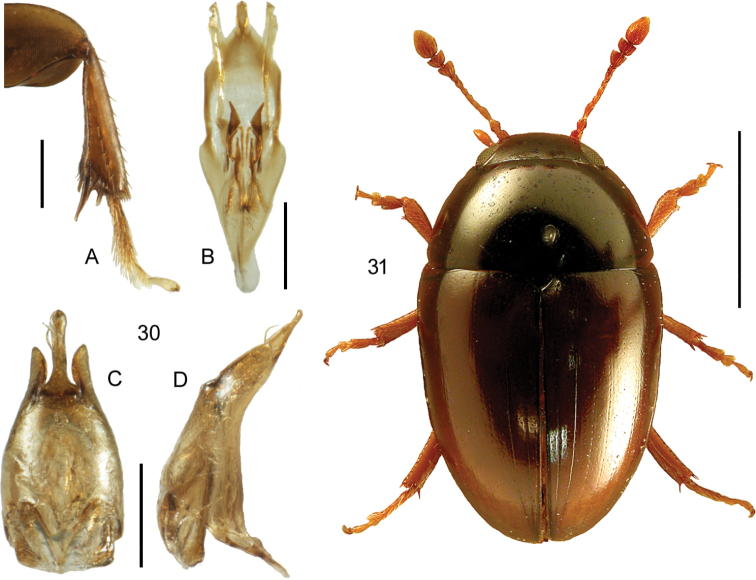
**30***Acylomusergoti* Casey **30A** male metatibia, ventral view **30B** aedeagus, dorsal view **30C** tegmen, dorsal view **30D** tegmen, lateral view **31***Olibrusliquidus* Erichson, habitus, L. Borowiec. Scale bars: 0.2 mm (**30A–D**), 1.0 mm (**31**).

(based on the redescription of *Tinodemusgrouvellei* by Švec 2002). Body length 2.0 mm. Oval, dark brown, elytral suture and base of pronotum laterally paler. Legs, antennae and mouthparts red, ventral side orange. Head and pronotum without microsculpture. Scutellum and elytra finely and densely transversely strigose. Sutural stria of elytra present in the apical 5/8. Male metatibiae short and widened apically, twice as wide at apex as proximally, medio-apical spine ca. twice as long as metatarsomere 1, curved at apex (Fig. 30A). Female metatibiae not modified. Male genitalia as in Fig. 30B, C.

###### Bionomic notes.

The Canadian specimens were collected in various habitats (grasslands, forests, wetlands, residential areas etc.), mainly with Malaise traps.

###### Comments.

The lack of a modern species-level revision of *Acylomus* prevents detailed comparison to most other Nearctic species of *Acylomus*. The only other species of *Acylomus* previously known from Canada, *A.pugetanus* Casey, 1916, was redescribed by Steiner and Singh (1987). It is darker, especially ventrally, and has different male genitalia and no apparent sexual dimorphism in metathoracic leg structure. At least one more species of *Acylomus* (BOLD:ACM7465 ) occurs in Canada according to DNA barcode data and initial morphological analysis of the barcoded specimens, but we have not been able to identify it to species level.

##### Olibrus liquidus

Taxon classificationAnimaliaColeopteraPhalacridae

Erichson, 1845

50676576-D421-5074-A4C1-EBB90F9C0F09

Figure 31

###### Distribution.

Native to the Palaearctic region, widespread in Europe and North Africa (Švec 2007). Adventive in the Nearctic region (British Columbia, Canada).

###### Canadian records.

British Columbia: Burnaby, 20-Apr-2015 to 08-May-2015 (1 ex, CBG).

###### Diagnostic information

(based on Thompson 1958; Vogt 1967b). Body length 1.9–2.6 mm. Habitus (Fig. 31) elongate-oval, narrower towards the elytral apex. Dark brown, elytra paler towards the apex, antennae, palpi and legs yellow-brown. Head and pronotum without microsculpture. Elytra fully covered by fine, net-like microsculpture in females, anterior third without microsculpture in males. Elytra with two sutural striae, which converge and usually meet towards the apex. Metaventrite densely and coarsely punctate, especially laterally. See Thompson (1958) for figures of the male genitalia (tegmen) and female ovipositor.

###### Bionomic notes.

This species feeds on a variety of Asteraceae genera, usually in dry and warm habitats in Europe (Koch 1989b). The Canadian specimen was collected with a Malaise trap in a suburban residential area.

###### Comments.

Lack of a modern revision of North American *Olibrus* prevents detailed comparison of *O.liquidus* to the native species. It is most reliably identified using male genitalia or DNA barcodes. Good illustrations of the genitalia are provided in volume 5, part 5b of the Handbooks for the Identification of British Insects (Thompson 1958).

### Nitidulidae

#### Epuraeinae

##### Epuraeini

###### Epuraea unicolor

Taxon classificationAnimaliaColeopteraNitidulidae

(Olivier, 1790)

5CC45DF9-AABD-5743-A45E-212183FE23C1

Figure 32

####### Distribution.

Native and widespread in the Palaearctic region. Recorded from North Africa and all of Europe to the Russian Far East and Japan (Audisio 1993; Jelínek and Audisio 2007) One of the most common and abundant species of the genus in Europe (Audisio 1993). Adventive in the Nearctic region (Ontario, Canada).

####### Canadian records.

Ontario: Guelph, 01-Nov-2009 (1 ex, CBG); Guelph, 22-Apr-2017 (1 ex, CBG); Guelph, 06-Jun-2018 (3 exx, CBG); Guelph, 30-Jun-2018 (1 ex, CBG); Rouge National Urban Park, 03-Jun-2013 to 09-Jun-2013 (1 ex, CBG).

####### Diagnostic information

**Figure 32.**
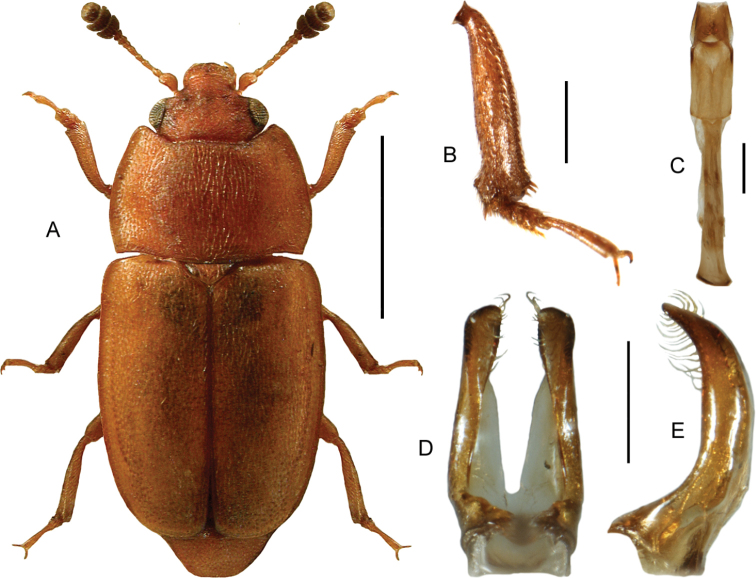
*Epuraeaunicolor* (Olivier) **A** habitus, L. Borowiec **B** male mesotibia **C** penis **D** parameres, ventral view **E** parameres, lateral view. Scale bars: 1.0 mm (**A**), 0.2 mm (**B–E**).

(based on Audisio 1993). Body length 2.3–3.2 mm. Habitus elongate, subparallel, rather flattened (Fig. 32A). Color variable, body, legs, and antennae usually yellowish or red-brown, pronotum and elytra often laterally paler, antennal club usually darkened. Elytra variably darkened, with a rounded dark spot on each elytron at the apical third, or with more extensive, irregular but symmetric dark patterns. Antennae with club ca. 1.5 times as long as wide. Head with subcircular, moderately impressed punctation, punctures approximately the size of the ommatidia, separated by 0.5–0.6 times their diameter, interspace with fine microsculpture. Punctures on pronotum and elytra slightly larger, but with similar microsculpture and relative distance between them. Pronotum 1.45–1.65 times as wide as long, broadest in the basal third, abruptly narrowed towards the protruding hind angles, anterior edge with a deep, wide, trapezoidal emargination. Elytral apices separately broadly rounded. Metaventrite with a wide V-shaped emargination at the hind edge. Male mesotibia distally slightly widened, with a small tooth at the inner margin (Fig. 32B). Female mesotibia unmodified. Male genitalia as in Fig. 32C−E.

####### Bionomic notes.

This species occurs in decaying and fermenting organic material (e.g., fruit, fruiting bodies of fungi, tree sap), under the bark of dead trees etc., probably feeding on the microbes decomposing these materials (Audisio 1993). Often found in anthropogenic habitats such as orchards, cultivated fields, and garbage dumps (Audisio 1993). The Canadian specimens were collected by sifting a compost heap in a suburban backyard, in a Malaise trap in a residential area, and in pitfall traps at a riverside in Rouge National Urban Park.

####### Comments.

The lack of a modern revision of North American *Epuraea* prevents detailed comparison to other Canadian species at the moment. *Epuraeaunicolor* can be reliably separated by DNA barcodes from all other Palaearctic and Nearctic *Epuraea* species sampled so far. The diagnostic information above, in particular the male mesotibia and genitalia, should allow morphological identification.

### Coccinellidae

#### Coccinellinae

##### Chilocorini

###### Chilocorus renipustulatus

Taxon classificationAnimaliaColeopteraCoccinellidae

(Scriba, 1791)

23520E20-69BE-5FFF-BD1C-53AA912C2451

Figure 33

####### Distribution.

Native to the Palaearctic region. Widespread in Europe, also recorded from Siberia and the Russian Far East (Kovář 2007). Adventive in the Nearctic region (Ontario, Canada).

####### Canadian records.

Ontario: Hamilton, 22-Sep-2014 to 03-Oct-2014 (1 ex, CBG); Mississauga, 19-Sep-2016 to 30-Sep-2016 (3 exx, CBG); Windsor, 22-Sep-2014 to 03-Oct-2014 (1 ex, CBG).

####### Diagnostic information

**Figures 33, 34.**
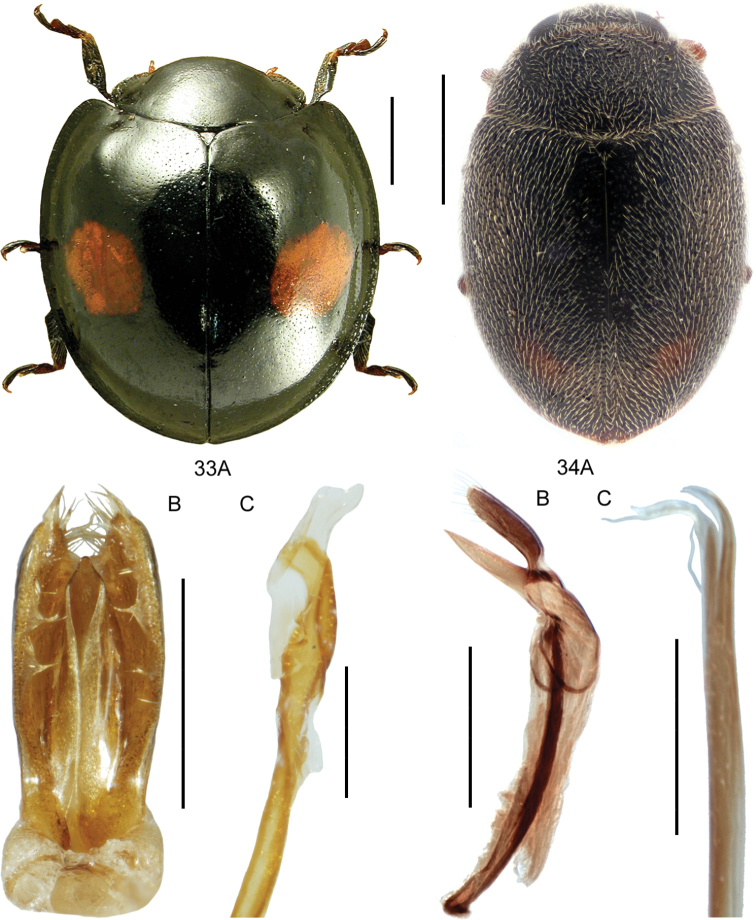
**33***Chilocorusrenipustulatus* (Scriba) **33A** habitus, L. Borowiec **33B** median lobe (penis guide) and parameres, ventral view **33C** penis (sipho), tip in lateral view **34***Nephusbisignatus* (Boheman) **34A** habitus, S. Karjalainen **34B** median lobe (penis guide) and parameres, lateral view **34C** penis (sipho), tip in lateral view. Scale bars: 1.0 mm (**33A**), 0.5 mm (**33B; 34A**), 0.2 mm (**33C; 34B**), 0.1 mm (**34C**).

(based on Fürsch 1967 and Gordon 1985). Body length 4–5 mm. Habitus as in Fig. 33A. Black, shiny, with a single rounded or slightly transverse orange-red macula on each elytron, abdomen laterally and apically orange, medial part of first ventrite black. Pronotum without distinct microsculpture on disc. Male genitalia as in Fig. 33B, C.

####### Bionomic notes.

The main habitat in Europe is broadleaf forest, and the preferred prey are scale insects, in particular *Chionaspissalicis* (Linnaeus, 1758) (Koch 1989b). The Canadian specimens were collected with Malaise traps in suburban residential areas.

####### Comments.

*Chilocoruskuwanae* Silvestri, 1909, an East Asian species introduced to the United States and recorded from across the country (Gordon 1985; Hendrickson et al. 1991), was recently synonymized with *C.renipustulatus* by Bieńkowski (2018). According to Bieńkowski, male genitalia are similar throughout the distribution areas of both species. However, Bieńkowski did not study any type material. One of the Canadian specimens shares an identical barcode haplotype with specimens of *C.renipustulatus* from Germany and Finland, others are slightly divergent (p-distance to European material varies from 0.006 to 0.015). Unfortunately, no barcode data are available for *C.kuwanae*. No Canadian records have been previously published under either name.

*Chilocorusrenipustulatus* is externally very similar to *Chilocorusstigma* (Say, 1835) and its closest relatives. It can be distinguished using the male genitalia and microsculpture of the pronotum. In *C.stigma* and allied species, the interspace between punctures on the disc of the pronotum is covered by finely engraved, netlike microsculpture. In *C.renipustulatus*, the interspace is smooth and shiny, with no visible microsculpture on disc. The orange maculae on the elytra are more transverse in *C.renipustulatus* than in *C.stigma* in the examined DNA barcoded Canadian material of these species, but the maculae are known to vary in size and shape in *C.renipustulatus* (Bieńkowski, 2018).

#### Coccinellinae

##### Scymnini

###### Nephus bisignatus

Taxon classificationAnimaliaColeopteraCoccinellidae

(Boheman, 1850)

21ED9D00-822A-5702-ADB2-87FF86DE1ACC

Figure 34

####### Distribution

Previously known only from Europe, where the species is more common in the north and rather sporadic in the central and southern parts (Fürsch 1965; Kovář 2007; Silfverberg 2010; Rassi et al. 2015). Probably Holarctic and previously overlooked in North America.

####### Canadian records.

Nunavut: Kugluktuk, 25-Jun-2010 (1 ex, CNC); Kugluktuk, 01-Jul-2010 (1 ex, CNC); Kugluktuk, 11-Jul-2010 (1 ex, CNC); Kugluktuk, 13-Jul-2010 (1 ex, CNC).

####### Diagnostic information

(based on Fürsch 1965, 1967, 1987). Body length 1.5–2.0 mm. Habitus elongate-oval (Fig. 34A). Black, with the anterior edge of the pronotum and often the apical edge of the elytra narrowly brown. Each elytron with a single small, obscurely delimited red-brown spot close to the apex, sometimes very faintly visible. Antennae with nine antennomeres. Pronotum very finely punctate, with strong, netlike microsculpture. Postcoxal lines on first abdominal ventrite briefly parallel to the hind margin of the ventrite at the middle, with the apices curved forward laterally. Male genitalia as in Fig. 34B, C.

####### Bionomic notes.

*Nephusbisignatus* prefers open, usually sandy habitats in Europe (Koch 1989b). The Canadian specimens were collected in mesic tundra with yellow pan and pitfall traps.

####### Comments.

*Nephusbisignatus* belongs to subgenusBipunctatus Fürsch, 1987, which is characterized by having only nine antennomeres (Fürsch 1987). All the previously recorded Canadian species have either ten or eleven antennomeres (Gordon 1976, 1985). The remote collecting localities in the arctic tundra indicate that this species is probably Holarctic and previously overlooked in North America rather than adventive from the Palaearctic region. Two subspecies are known from Europe (Kovář 2007), but we refrain from assigning the Canadian specimens to any subspecies. Among the Nearctic fauna, *N.bisignatus* resembles *N.georgei* (Weise, 1929), but has a narrower body outline and usually smaller and less conspicuous elytral spots. *Nephusgeorgei* also has ten antennomeres instead of nine. Gordon (1976) notes that specimens of *N.georgei* from the northern parts of the Northwest Territories are smaller and narrower compared to specimens from southern Canada and northern United States, and that the pale color pattern of the elytra is reduced in the northern specimens. Based on these notes, the arctic specimens of *N.georgei* may actually represent *N.bisignatus* and need to be re-examined.

###### Scymnus rubromaculatus

Taxon classificationAnimaliaColeopteraCoccinellidae

(Goeze, 1777)

28ACFC8F-58E2-5830-B81D-95EFA3D125EE

Figure 35

####### Distribution.

Native to the Palaearctic region, widespread across Eurasia from western Europe to the Russian Far East (Kovář 2007). Adventive in the Nearctic region (Ontario and Nova Scotia, Canada).

####### Canadian records.

Ontario: Barrie, 22-Apr-2013 to 03-May-2013 (1 ex, CBG); Burlington, 21-Jul-2017 (1 ex, CBG); Guelph, 06-Jun-2013 to 13-Jun-2013 (1 ex, CBG); Guelph, 20-Jun-2013 to 27-Jun-2013 (2 exx, CBG); Guelph, 01-Aug-2013 to 08-Aug-2013 (1 ex, CBG); Guelph, 15-Aug-2013 to 22-Aug-2013 (1 ex, CBG); Mississauga, 15-Sep-2015 to 17-Sep-2015 (1 ex, CBG); Mississauga, 19-Sep-2016 to 30-Sep-2016 (3 exx, CBG); Toronto, 19-Jun-2017 to 27-Jun-2017 (1 ex, CBG). Nova Scotia: Berwick, 20-Apr-2015 to 08-May-2015 (1 ex, CBG).

####### Diagnostic information

**Figure 35.**
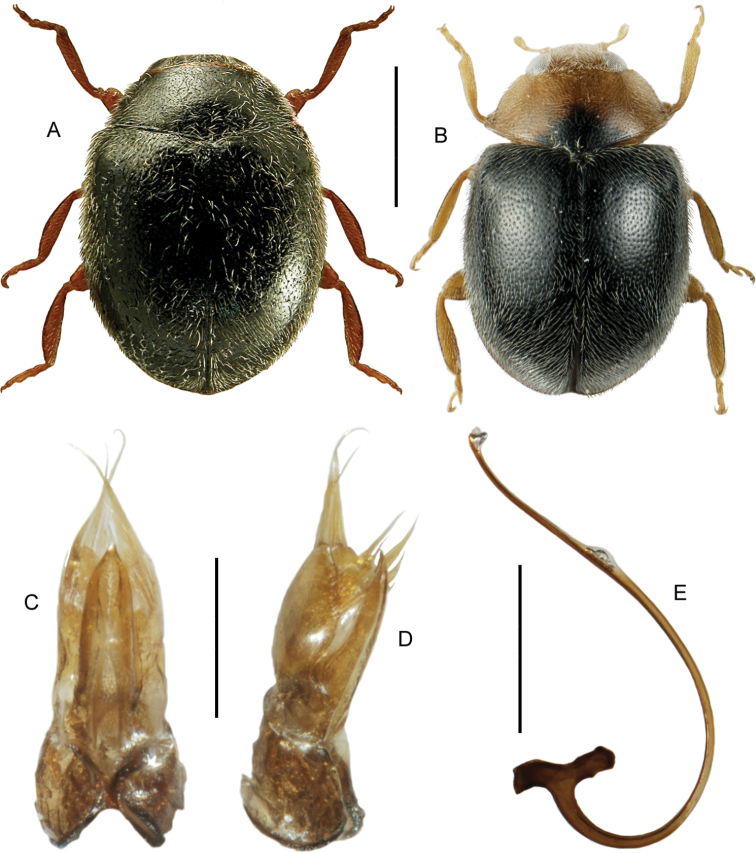
*Scymnusrubromaculatus* (Goeze) **A** female habitus, L. Borowiec **B** male habitus **C** median lobe (penis guide) and parameres, ventral view **D** median lobe (penis guide) and parameres, lateral view **E** penis (sipho), lateral view. Scale bars: 1.0 mm (**A, B**), 0.2 mm (**C, D**), 0.5 mm (**E**).

(based on Fürsch 1967). Body length 1.8–2.3 mm. Habitus as in Fig. 35A, B. Color sexually dimorphic. Male: head, pronotum (apart from a black mediobasal spot), and legs yellow, otherwise black. Female: almost completely black with only the mouthparts, labrum and legs yellow. Femora often darkened. Postcoxal lines on 1^st^ abdominal ventrite reaching the hind edge of the ventrite. Male genitalia as in Fig. 35C−E.

####### Bionomic notes.

This species prefers dry, warm habitats in Europe and is found mainly on Brassicaceae, occasionally on trees and bushes (Koch 1989b). Most Canadian specimens were collected with Malaise traps in suburban areas.

####### Comments.

*Scymnusrubromaculatus* leads to the couplets separating *Scymnusamericanus* Mulsant, 1850, *S.apicanus* Chapin, 1973 and *S.paracanus* Chapin, 1973 in Gordon’s keys to North American *Scymnus* (Gordon 1976, 1985). In *S.rubromaculatus*, the dorsal surface is more densely punctate and pubescent than in those three species, the hind margin of the elytra is slightly or not paler than the elytral disc, and the female has no pale markings on head (apart from labrum and mouthparts) or pronotum. The male genitalia differ: the apical hook of the penis (sipho), which is typical for *S.americanus* and related species, is absent in *S.rubromaculatus*.

### Corylophidae

#### Corylophinae

##### Orthoperini

###### Orthoperus corticalis

Taxon classificationAnimaliaColeopteraCorylophidae

(Redtenbacher, 1845)

89EC2E44-CEC0-53CD-A185-25030A4B2783

Figure 36

####### Distribution.

Native to the Palaearctic region. Widely distributed from Western Europe to Siberia (Bowestead 1999, 2007). Adventive in the Nearctic region (Ontario, Canada).

####### Canadian records.

Ontario: Cambridge, 29-Apr-2015 to 07-May-2015 (1 ex, CBG); Cambridge, 07-May-2015 to 14-May-2015 (1 ex, CBG); Cambridge, 21-May-2015 to 27-May-2015 (1 ex, CBG).

####### Diagnostic information

**Figures 36, 37.**
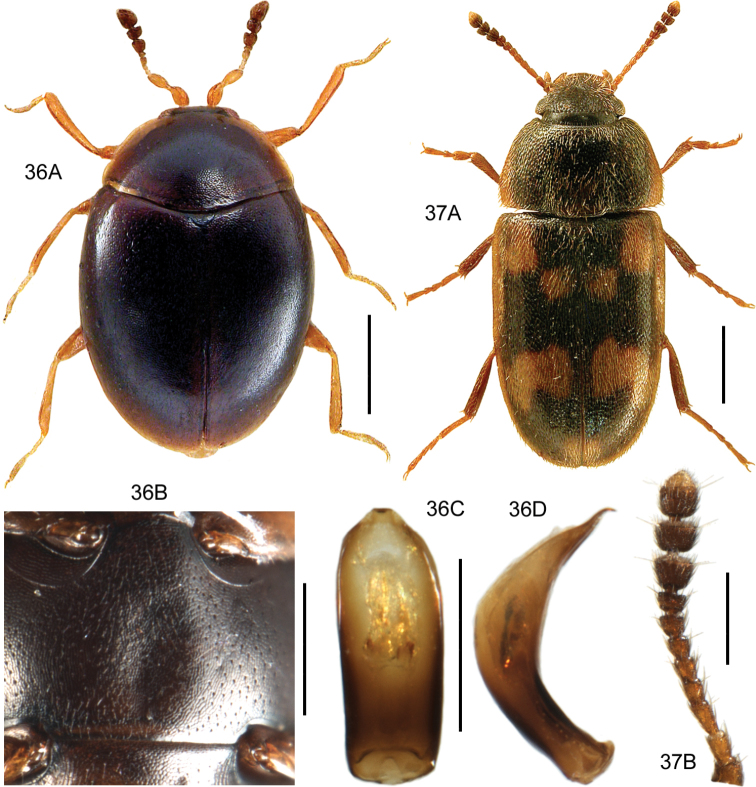
**36***Orthoperuscorticalis* (Redtenbacher) **36A** habitus, L. Borowiec **36B** male metaventrite **36C** aedeagus, ventral view **36D** aedeagus, lateral view **37***Litargusconnexus* (Geoffroy) **37A** habitus, L. Borowiec **37B** antenna. Scale bars: 0.25 mm (**36A**), 0.2 mm (**36B–D**), 0.5 mm (**37A**), 0.2 mm (**37B**).

(based on Bowestead 1999). Body length 0.8–1.0 mm. Habitus slightly elongate oval, strongly convex (Fig. 36A). Dark brown to black, antennal base and legs pale, five apical antennomeres dark brown. Pronotum finely punctate, often with a transverse row of larger punctures medioposteriorly, with isodiametric microsculpture throughout. Elytral punctation fine, punctures larger basally, interspaces with similar microsculpture as the pronotum, the microsculpture forming wavy transverse rows of cells especially basally. Sutural striae of elytra present only at the apex. Male metaventrite with an elongate depression medially, and a short median keel behind the depression, distance of the keel from hind edge of metaventrite ca. 1/12 of the length of the metaventrite (Fig. 36B). Aedeagus as in Fig. 36C, D.

####### Bionomic notes.

This species is mainly known from deciduous forests. It has been collected from a variety of fungus species growing on dead logs, and under the bark of fungus-infested logs (Bowestead 1999, Rutanen 2015). The Canadian specimens were collected with a Malaise trap at the edge of a forest.

####### Comments.

This is the second species of *Orthoperus* recorded as adventive in Canada: the Palaearctic *O.atomus* (Gyllenhal, 1808) is known from British Columbia in Canada, and Washington and Oregon in the United States (Klimaszewski et al. 2015). *Orthoperuscorticalis* is darker and slightly larger than *O.atomus*, with stronger punctation on the pronotum and elytral base and denser and more strongly impressed microsculpture (Bowestead 1999). Two native North American species are currently known from Canada: *O.scutellaris* LeConte, 1878 has small V-shaped scratches on the elytra instead of punctures, and *O.suturalis* LeConte, 1878 has fine but distinctly impressed sutural striae (only faintly visible close to the elytral apex in *O.corticalis*) (LeConte 1878, Downie and Arnett 1996).

### Mycetophagidae

#### Mycetophaginae

##### Mycetophagini

###### Litargus connexus

Taxon classificationAnimaliaColeopteraMycetophagidae

(Geoffroy, 1785)

66BCF5E3-CE40-52FA-862F-A90D4FCE3E9E

Figure 37

####### Distribution.

Native to the Palaearctic region. Widespread in Europe, also recorded from North Africa, and across the region to the Russian Far East and Japan (Nikitsky 2008). Adventive in the Nearctic region (British Columbia, Canada).

####### Canadian records.

British Columbia: Burnaby, 20-Apr-2015 to 08-May-2015 (1 ex, CBG).

####### Diagnostic information

(based on Vogt 1967a). Body length 2.4–2.8 mm. Dorsal habitus elongate, sides of elytra almost parallel (Fig. 37A). Black, elytra with transverse, undulating yellow bands at the base and just beyond midlength, and a yellow sutural spot close to the elytral apex. Yellow markings variable, basal band frequently broken into separate spots. Antennomere 8 somewhat wider than long, terminal antennomere approximately as long as wide, structure of the antennal club as in Fig. 37B. Elytra with epipleura concave, descending towards the lateral edge.

####### Bionomic notes.

This species is found in deciduous and mixed forests in fungus-infested dead wood (Koch 1989b). The Canadian specimen was collected with a Malaise trap in a suburban residential area.

####### Comments.

The combination of the elongate and nearly parallel-sided body, color pattern of the elytra and structure of the antennae will distinguish *L.connexus* from all other *Litargus* species known from Canada. Parsons (1975) provides diagnoses and illustrations of the native North American species.

### Ciidae

#### Ciinae

##### Ciini

###### Cis boleti

Taxon classificationAnimaliaColeopteraCiidae

(Scopoli, 1763)

BE06AB87-8049-583F-BCE6-C69A075D54A5

Figure 38

####### Distribution.

Native to the Palaearctic region. Recorded across Eurasia from western Europe to the Russian Far East and Japan (Jelínek 2008). Adventive in the Nearctic region (Ontario, Canada).

####### Canadian records

(barcoded specimens). Ontario: Guelph, 21-Aug-2017 (2 exx, CBG); Guelph, 21-Oct-2017 (2 exx, CBG);

####### Additional Canadian records.

Ontario: Horning’s Mills, 08-Nov-2015 (1 ex, DEBU); Milton, 28-May-2002 (1 ex, DEBU).

####### Diagnostic information

**Figures 38, 39.**
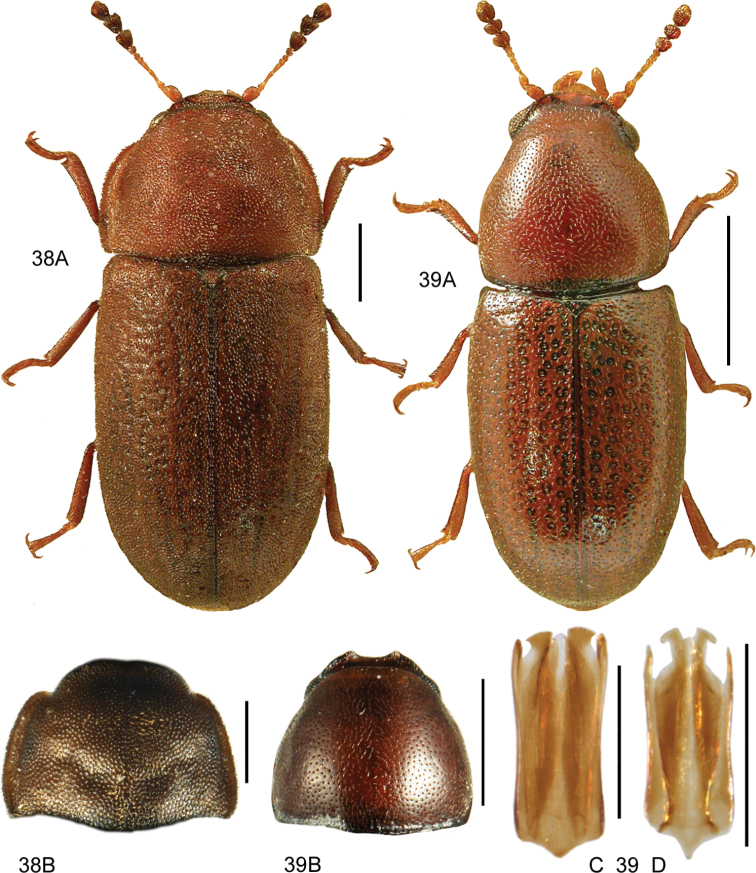
**38***Cisboleti* (Scopoli) **38A** habitus, L. Borowiec **38B** pronotum **39A***Cisglabratus* Mellié, female habitus, L. Borowiec **39B***Cisglabratus*, male head and pronotum **39C***Cisglabratus*, aedeagus **39D***Cislevettei* (Casey), aedeagus. Scale bars: 0.5 mm (**38A, B; 39A, B**), 0.2 mm (**39C, D**).

(based on Lohse 1967). Body length 2.8–4.0 mm. Habitus as in Fig. 38A. Dark brown to black (usually darker than in Fig. 38A). Pronotum more than 1.3 times wider than long, with concavities on disc (Fig. 38B). Lateral margins of pronotum widely deplanate and visible throughout in dorsal view. Elytra with dual punctation: larger punctures arranged in irregular rows, with the intervals finely and densely punctate. Elytral vestiture consists of short, stout bristles.

####### Bionomic notes.

This species feeds on polypore fungi, mainly *Trametes* species growing on deciduous trees (Koch 1989b, Reibnitz 1999). The barcoded Canadian specimens were collected from polypore fruiting bodies in a mixed forest.

####### Comments.

*Cissubmicans* Abeille de Perrin, 1874 (= *C.pistoria* Casey, 1898) is the only other representative of the mainly Palaearctic *C.boleti* species group known from North America (Lopes-Andrade et al. 2016). *Cisboleti* is a robust species, broader and on average larger than *C.submicans*, with the pronotum at least 1.3 times wider than long. The pronotal disc of *C.boleti* has indentations on both sides of the midline. These indentations are very shallow in *C.submicans*. The pronotum is also more densely punctate in *C.boleti* than in *C.submicans*.

###### Cis glabratus

Taxon classificationAnimaliaColeopteraCiidae

Mellié, 1848

ACA8288B-16A4-521E-9483-038690F1F69B

Figure 39

####### Distribution.

Native to the Palaearctic region, widespread in Europe (Jelínek 2008). Common in northern Europe, mainly found in higher elevations in Central Europe (Reibnitz 1999, Rassi et al. 2015). Adventive in the Nearctic region (Nova Scotia, Canada).

####### Canadian records.

Nova Scotia: Cape Breton Highlands National Park, 21-Jul-2009 (1 ex, CBG).

####### Diagnostic information

(based on Lohse 1967). Body length 1.5–2.0 mm. Red-brown to dark brown, habitus as in Fig. 39A. Clypeus in male with two large, broad teeth (Fig. 39B). Pronotum widest behind middle, distinctly tapering towards the front angles. Vestiture on pronotum fine and pale. Outer edge of protibia serrated. Male with a large abdominal fovea on 1^st^ abdominal ventrite. Aedeagus as in Fig. 39C.

####### Bionomic notes.

The main host fungus in Europe is *Fomitopsispinicola* (Sw.) P. Karst. (Reibnitz 1999). The Canadian specimen was collected in a jack pine forest in Cape Breton Highlands National Park.

####### Comments.

*Cisglabratus* is externally very similar to *C.levettei* (Casey, 1898) and leads to that species in the key to North American species (Lawrence 1971). The microscopic vestiture of the pronotum is longer and more conspicuous in *C.glabratus*, but the most reliable morphological differences are in the male genitalia (Fig. 39C, D). *Cislevettei* forms a separate BIN (BOLD:ACA7530 ) which is more closely clustered to other Palaearctic members of the *C.nitidus* species group (*C.castaneus* (Herbst, 1793), *C.jacquemartii* Mellié, 1848 and *C.lineatocribratus* Mellié, 1848) than to *C.glabratus*.

### Mordellidae

#### Mordellinae

##### Mordellistenini

###### Mordellistena militaris

Taxon classificationAnimaliaColeopteraMordellidae

LeConte, 1862

E3A85312-46A7-57A9-8166-3E4FB57FA954

####### Distribution.

Native to the Nearctic region. Previously recorded at least from Indiana, New York, North Carolina, and Ohio in the United States (Liljeblad 1945; Downie and Arnett 1996).

####### Canadian records.

Ontario: Point Pelee National Park, 27-Jun-2012 to 04-Jul-2012 (5 exx, CBG).

####### Diagnostic information.

See Liljeblad (1945).

####### Bionomic notes.

The Canadian specimens were collected with a Malaise trap in a savanna with *Opuntia* cacti and sparse woody vegetation.

####### Comments.

The coloration and the ridges of the hind legs of the Canadian specimens match both the Liljeblad (1945) diagnosis and the photographs of LeConte’s type specimen in the type database of the Museum of Comparative Zoology at Harvard University. Therefore, we consider this record reliable despite the lack of a modern revision of the North American *Mordellistena*.

### Zopheridae

#### Colydiinae

##### Synchitini

###### Lasconotus subcostulatus

Taxon classificationAnimaliaColeopteraZopheridae

Kraus, 1912

2F4D7DED-068C-553D-A9D3-322CCBB2D6F2

####### Distribution.

Native to the Nearctic region. Previously known from California, Idaho, Nevada, Oregon, Washington, South Dakota, Montana, and Nebraska in the United States (Lord et al. 2011–2013).

####### Canadian records

(DNA barcoded specimen). Saskatchewan: Grasslands National Park 21-May-2014 to 29-May-2014 (1 ex, CBG).

####### Additional Canadian records.

British Columbia: Aspen Grove, 20-Oct-1936 (5 exx, CNC); Merritt, 04-Jun-1922 (1 ex, CNC); Merritt, 08-Jun-1922 (2 exx, CNC); Merritt, 09-Jun-1922 (6 exx, CNC); Merritt, 10-Jun-1922 (1 ex, CNC); Merritt, 15-Jun-1922 (2 exx, CNC); Merritt, 18-Jun-1922 (1 ex, CNC); Merritt, 14-Sep-1923 (1 ex, CNC); Merritt, 03-Jun-1924 (1 ex, CNC); Merritt, 13-May-1925 (1 ex, CNC); Merritt, 25-Jul-1925 (1 ex, CNC); Olivier, 24-May-1958 (1 ex, CNC); Olivier, 12-Jun-1958 (10 exx, CNC); Olivier, 14-Jun-1958 (6 exx, CNC); Peachland, 19-Jul-1912 (1 ex, CNC); Peachland, 13-Jul-1919 (1 ex, CNC); Summerland, 25-Mar-1932 (16 exx, CNC); Summerland, 24-Sep-1932 (5 exx, CNC); Summerland, 7-Oct-1932 (1 ex, CNC); Summerland, 10-Oct-1932 (116 exx, CNC); Summerland, 11-Oct-1932 (5 exx, CNC); Summerland, 11-Nov-1932 (51 exx, CNC); Exact locality unknown, Sep-1923 (1 ex, CNC). Saskatchewan: Crane Valley, 06-Oct-1914 (1 ex, CNC). Manitoba: Aweme, 25-Jul-1919 (5 exx, CNC); Aweme, 31-Oct-1921 (1 ex, CNC); Onah, 24-Jul-1919 (5 exx, CNC).

####### Diagnostic information

(based on Stephan 1989 and Lord et al. 2011–2013). Body length 2.5–2.8 mm. Pronotum with a central concave area covering 1/3 to 1/2 total width of pronotum, concave area bordered laterally by longitudinal raised ridges. Pronotum carinate anteriorly with double “U” shaped anterior margin. Elytral interstriae 5 more raised than other interstriae, forming a median concave area of the elytra typically on posterior half only. See Lord et al. (2011–2013) for a habitus photograph.

####### Bionomic notes.

Hackwell (1973) reported that this species is associated with galleries of several species of bark beetles where it feeds on both fungi and bark beetles during larval development. Many of the CNC specimens were collected from pine trees (*Pinuscontorta* Douglas ex Loudon, *P.monticola* Douglas ex D.Don, *P.ponderosa* Douglas ex C.Lawson). The DNA barcoded Canadian specimen was collected with a Malaise trap in a grassland.

####### Comments.

The single DNA barcoded specimen from Saskatchewan (the only member of its BIN, with no closely clustered neighbors) was compared with specimens of this little-studied genus in the CNC. The identification of this specimen using data in Lord et al. (2011–2013) led to the further identification of several other Canadian specimens from British Columbia, Saskatchewan, and Manitoba. Examination of specimens collected 100 years ago in three provinces suggests that this species has long been part of the Canadian fauna.

### Tenebrionidae

#### Alleculinae

##### Alleculini

###### Isomira angusta

Taxon classificationAnimaliaColeopteraTenebrionidae

(Casey, 1891)

111EC2CC-C223-5A51-9B21-B85D023A01E9

Figure 40

####### Distribution.

Native to the Nearctic region. Previously known from Georgia and South Carolina in the United States (Bousquet et al. 2018).

####### Canadian records.

Ontario: Point Pelee National Park, 23-Jun-2010 (2 exx, CBG).

####### Diagnostic information

**Figure 40.**
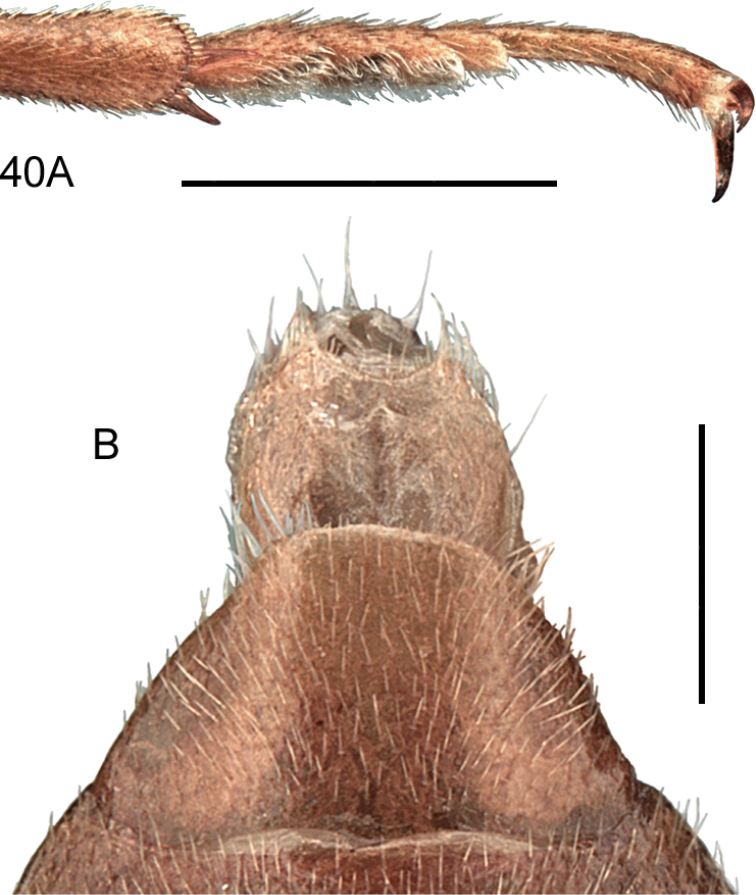
*Isomiraangusta* (Casey) **A** anterior tarsal pubescence **B** male sternite 8. Scale bars: 0.5 mm (**A**), 1.0 mm (**B**).

(based on Aalbu et al. 2002). Body length 5.5–6.0 mm. Ventral surface of tarsi densely, finely pubescent (Fig. 40A). Male with sternite VIII deeply bilobed apically (Fig. 40B), extending beyond posterior edge of abdominal ventrite 5.

####### Bionomic notes.

The Canadian specimens were collected with a UV light trap in a meadow patch in deciduous forest.

####### Comments.

This species was originally described as the only member of the new genus *Tedinus* by Casey (1891). *Tedinus* was included as valid in the key to the genera of Alleculini by Aalbu et al. (2002) where it was separated from species of *Isomira* Mulsant, 1856 based on the characters listed above. In addition to the new Canadian record, three new U.S. state records were found among the DNA barcoded specimens: Florida: Destin, 25-Mar-1980 (1 ex, CNC). Oklahoma: Willis, 15-Apr-2009 (6 exx, CBG & CNC); Willis, 18-Apr-2009 (1 ex, CNC). Illinois: Pine Hills Field Station, 22-May-1967 (1 ex, CNC).

### Chrysomelidae

#### Galerucinae

##### Alticini

###### Chaetocnema hortensis

Taxon classificationAnimaliaColeopteraChrysomelidae

(Geoffroy, 1785)

92C5A3EB-6A7D-5B9B-95D2-359095A879DA

Figure 41

####### Distribution.

Native to the Palaearctic region, widespread across the region and common in many parts (Döberl 2010; Konstantinov et al. 2011). Adventive in the Nearctic region (Ontario, Canada).

####### Canadian records.

British Columbia: Kelowna, 22-Sep-2014 to 03-Oct-2014 (2 exx, CBG); Revelstoke, 21-Sep-2015 to 02-Oct-2015 (2 exx, CBG). Ontario: Brampton, 19-Sep-2016 to 30-Sep-2016 (2 exx, CBG); Mississauga, 24-May-2016 to 26-May-2016 (1 ex, CBG); Mississauga, 19-Sep-2016 to 30-Sep-2016 (1 ex, CBG). Nova Scotia: Cape Breton Highlands National Park, 23-Jun-2013 to 29-Jun-2013 (1 ex, CBG); Elmwood, 01-Nov-2005 (1 ex, CBG); Kejimkujik National Park, 31-Jul-2009 (1 ex, CBG); Truro, 21-Sep-2015 to 02-Oct-2015 (2 exx, CBG). Labrador: Happy Valley-Goose Bay, 19-Sep-2016 to 30-Sep-2016 (1 ex, CBG). Newfoundland: Terra Nova National Park, 04-Jul-2009 (1 ex, CBG).

####### Diagnostic information

**Figure 41.**
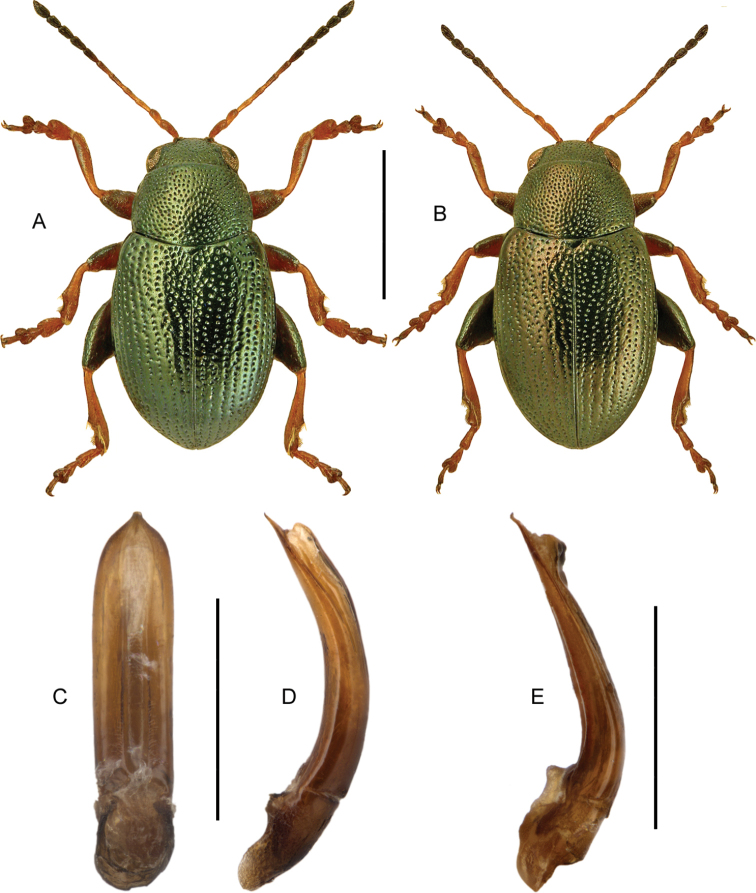
*Chaetocnemahortensis* (Geoffroy) **A** male habitus, L. Borowiec **B** female habitus, L. Borowiec **C***C.hortensis*, aedeagus, ventral view **D***C.hortensis*, aedeagus, lateral view **E***C.borealis* R. White, aedeagus, lateral view. Scale bars: 1.0 mm (**A, B**), 0.5 mm (**C–E**).

(based on Konstantinov et al. 2011). Body length (excluding head) 1.8–2.1 mm. Habitus as in Fig. 41A, B. Pronotum and elytra with a bronze or green metallic lustre. Four basal antennomeres yellow, antennomere 2 sometimes partly brown, femora brown, tibiae yellow. Pronotal punctures separated by approximately their own diameter. The two innermost elytral rows of punctures on basal half confused, third through fifth rows confused or regular, sixth row confused. Elytral humeral callus well developed. Aedeagus as in Fig. 41C, D.

####### Bionomic notes.

*Chaetocnemahortensis* has a wide range of host plants. It mainly feeds on various grasses (Poaceae), including cereal crop species (Koch 1992; Konstantinov et al. 2011). It has been recorded as a minor pest of wheat and barley in Europe (Pavlov 1960’ Vappula 1965). Most of the barcoded Canadian specimens were collected with Malaise traps in suburban environments. A few records are from grassland and forest habitats in Canadian national parks.

####### Comments.

*Chaetocnemahortensis* has previously been confused with *C.borealis* R. White, 1996 in Canada. We found that most Canadian specimens in CNC identified as *C.borealis* actually represent *C.hortensis*. The elytral punctation of the two species is similarly irregular basally. In *C.borealis*, the basal antennomeres are brown rather than pale yellow, and the dorsal surface has a blue rather than bronze or green lustre. The aedeagus is differently shaped in the two species (Fig. 41D, E). Based on comparison of the type specimens of *C.borealis* (deposited in CNC) with the diagnoses and figures in the recent revision of Palaearctic *Chaetocnema* species (Konstantinov et al. 2011), *C.borealis* is very similar to (and possibly synonymous with) the Palaearctic *C.sahlbergii* (Gyllenhal, 1827). Both species inhabit bogs and other types of wetlands (Koch 1992; White 1996). Records of *C.borealis* from agricultural fields and other drier habitats reported e.g., by Majka and LeSage (2010) probably represent *C.hortensis*.

###### Longitarsus lewisii

Taxon classificationAnimaliaColeopteraChrysomelidae

Baly, 1874

35747697-4DF9-58C7-8C57-5AA957073BB1

Figure 42

####### Distribution.

Native to the Palaearctic region. Widespread in Europe, recorded throughout Eurasia to China and the Russian Far East (Döberl 2010). Adventive in the Nearctic region (Ontario, Canada).

####### Canadian records.

Ontario: Cornwall, 19-Sep-2016 to 30-Sep-2016 (5 exx, CBG).

####### Diagnostic information

**Figure 42.**
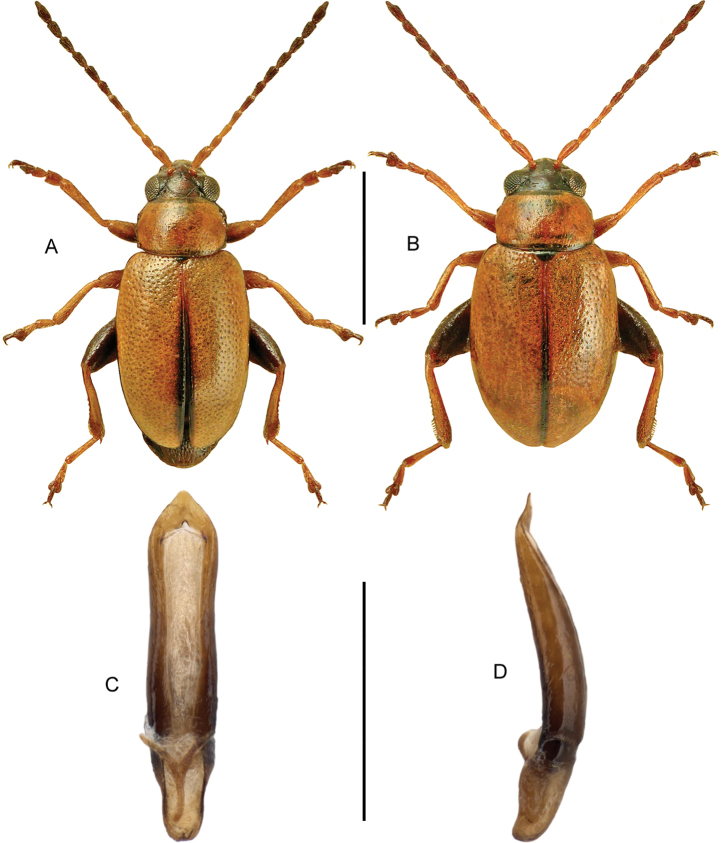
*Longitarsuslewisii* Baly **A** male habitus, L. Borowiec **B** female habitus, L. Borowiec **C** aedeagus, ventral view **D** aedeagus, lateral view. Scale bars: 1.0 mm (**A, B**), 0.5 mm (**C, D**).

(based on Warchalowski 1996 and Rutanen and Martikainen 2014). Body length 1.7–2.3 mm. Habitus as in Fig. 42A, B, convex in dorsal view, sides of elytra rounded. Head brown, pronotum and elytra yellow-brown, elytral suture usually narrowly dark at least near midlength, legs pale, metafemora darker. Ventral side red-brown to black. Pronotum ca. 1.5 times wider than long, finely punctate. Elytra densely and finely punctate, punctures slightly larger around scutellum. Male with a narrow longitudinal impression at the middle of the last ventrite, ending in a small, sharply delimited round pit. Last ventrite of females unmodified or with a very weak impression. Penis in lateral view strongly bent towards dorsum at the apex (Fig. 42C, D).

####### Bionomic notes.

This species feeds on *Plantago* species, especially *P.major* L. (Koch 1992, Rutanen and Martikainen 2014). In Finland, it is most often collected in dry, barren habitats (Rutanen and Martikainen 2014). The Canadian specimens were collected with a Malaise trap in a suburban residential area.

####### Comments.

*Longitarsuslewisii* is closely related to *L.pratensis* (Panzer, 1794), another adventive species from the Palaearctic region (Warchalowski 1996, Rutanen and Martikainen 2014). *Longitarsuslewisii* is more rounded and convex, and on average slightly larger than *L.pratensis* (1.7–2.3 mm vs. 1.4–2.1 mm) (Warchalowski 1996). The elytral suture is not darkened in *L.pratensis*, and the hind femora are paler. However, color is variable in this species group, and the male genitalia and the modifications of the last ventrite are the best distinguishing characters. In males of *L.pratensis*, the impression of the last ventrite is broad, circular and less sharply delimited than in males of *L.lewisii*. The penis of *L.pratensis* is shorter than that of *L.lewisii*, and less strongly bent. Females of *L.pratensis* have an elongate-oval, shallow medial impression on the last ventrite. The preferred host plant of *L.pratensis* is *Plantagolanceolata* L., but both species use several species of *Plantago* (Koch 1992; Döberl 1994; Rutanen and Martikainen 2014).

###### Lythraria salicariae

Taxon classificationAnimaliaColeopteraChrysomelidae

(Paykull, 1800)

465B67EB-F7E3-5873-A359-1A369023124B

Figure 43

####### Distribution.

Native to the Palaearctic region. Widespread in Europe, scattered records in Asia to East Siberia and Japan (Döberl 2010). Adventive in the Nearctic region (Ontario, Canada).

####### Canadian records.

Ontario: Cambridge, 25-May-2015 to 31-May-2015 (1 ex, CBG); Pickering, 24-Jun-2017 to 25-Jun-2017 (1 ex, CBG).

####### Diagnostic information

**Figure 43.**
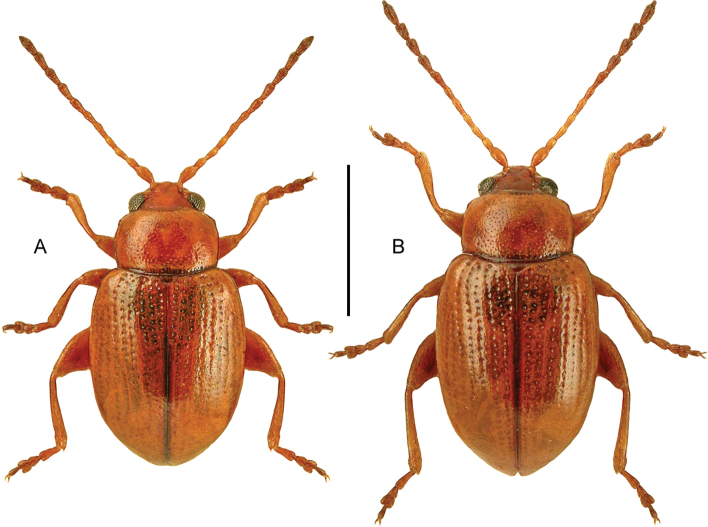
*Lythrariasalicariae* (Paykull), habitus, L. Borowiec **A** male **B** female. Scale bar: 1.0 mm.

(based on Mohr 1966). Body length 1.8–2.3 mm. Habitus elongate-oval (Fig. 43A, B). Yellow-brown or red-brown, apical antennomeres and ventral side darkened, sometimes also head, pronotum, and elytral suture darker brown. Base of pronotum without lateral furrows or a transverse impression. Procoxal cavities closed behind. Elytral punctures arranged in regular striae. Metatibia without a subapical dilation or tooth on the outer margin.

####### Bionomic notes.

*Lythrariasalicariae* is found in various wetland and marshy shoreline habitats as well as in forest depressions (Koch 1992). The larvae develop on *Lysimachia* species, and the adults occasionally feed also on *Lythrumsalicaria* L. (Koch 1992, Dolgovskaya et al. 2004). The Canadian specimens were collected with pan traps in a grassy wetland and a mixed habitat of agricultural fields and forest.

####### Comments.

*Lythraria* Bedel, 1897 is a monotypic genus reported here for the first time from North America. *Lythrariasalicariae* would be identified as *Pseudorthygia* Csiki, 1940 (couplet 75) using the key to genera of Galerucinae in Riley et al. (2002) based on its closed procoxal cavities, but *L.salicariae* is not as convex in lateral profile and has a more elongate body outline. Among previously recorded Canadian leaf beetles, the habitus of *L.salicariae* is somewhat similar to *Glyptinabrunnea* Horn, 1889, but the procoxal cavities are open behind in *Glyptina*.

#### Galerucinae

##### Luperini

###### Scelolyperus liriophilus

Taxon classificationAnimaliaColeopteraChrysomelidae

Wilcox, 1965

357F2C04-0430-55D3-AAEE-47BFA1A6E811

####### Distribution.

Native to the Nearctic region. Widespread in eastern United States (Clark 1996).

####### Canadian records.

Quebec: Forillon National Park, 05-Jul-2013 to 15-Jul-2013 (2 exx, CBG).

####### Diagnostic information.

See Clark (1996).

####### Bionomic notes.

This species has been collected from a wide variety of plant species (Clark 1996). The Canadian specimens were collected with a Malaise trap along a forest trail in Forillon National Park.

### Curculionidae

#### Brachycerinae

##### Erirhinini

###### Notaris scirpi

Taxon classificationAnimaliaColeopteraBrachyceridae

(Fabricius, 1793)

08EAD071-E9FD-5BF3-92A7-C89A7585D8E5

Figure 44

####### Distribution.

Native to the Palaearctic region. Widespread in Europe, with scattered records in Asia to the Russian Far East and Japan (Alonso-Zarazaga et al. 2017). Adventive in the Nearctic region (Quebec, Canada).

####### Canadian records

(DNA barcoded specimen). Quebec: Laval, 11-Jun-1997 (1 ex, CNC).

####### Additional Canadian records.

Quebec: Gatineau, 25-May-2012 (1 ex, CPTO); Henryville, 14-Jun-2015 (1 ex, CNC); Henryville, 07-Jun-2017 (1 ex, CMNC); Henryville, 07-Jun-2017 (9 exx, CCCH); Henryville, 14-Jun-2017 (1 ex, CMNC); Laval, 5-Jun-2004 (2 exx, CSDU); Laval, 19-Apr-2013 (1 ex, CSDU); Laval, 22-Jun-2013 (1 ex, CSDU); Longueuil, 21-May-2016 (1 ex, CPTO); Oka, 01-Jul-2004 (1 ex, CRVI); Oka, 06-Jun-2011 (1 ex, CRVI); Oka, 19-Aug-2012 (1 ex, CMNC); Oka, 26-Aug-2012 (1 ex, CPTO); Oka, 01-Jun-2014 (1 ex, CRVI); Oka, 21-Jun-2016 (1 ex, CRVI); Oka, 13-Jul-2016 to 20-Jul-2016 (2 exx, CRVI); Oka, 22-Jul-2016 (1 ex, CRVI); Oka, 15-May-2018 to 31-May-2018 (1 ex, CRVI); Saint-Côme, 13-Jul-2013 (1 ex, CSDU); Saint-Lazare, 20-Jun-2012 (1 ex, CPTO); Saint-Lazare, 17-Sep-2012 (1 ex, CPTO); Saint-Lazare, 14-Jun-2013 (1 ex, CPTO); Saint-Lazare, 18-Jul-2017 (1 ex, CSDU); Terrasse-Vaudreuil, 01-Jun-2013 (1 ex, CNC); Terrasse-Vaudreuil, 11-Jun-2007 (1 ex, CPTO); Terrasse-Vaudreuil, 30-Jun-2014 (1 ex, CPTO); Varennes, 07-Jun-2011 (1 ex, CCCH).

####### Diagnostic information

**Figures 44, 45.**
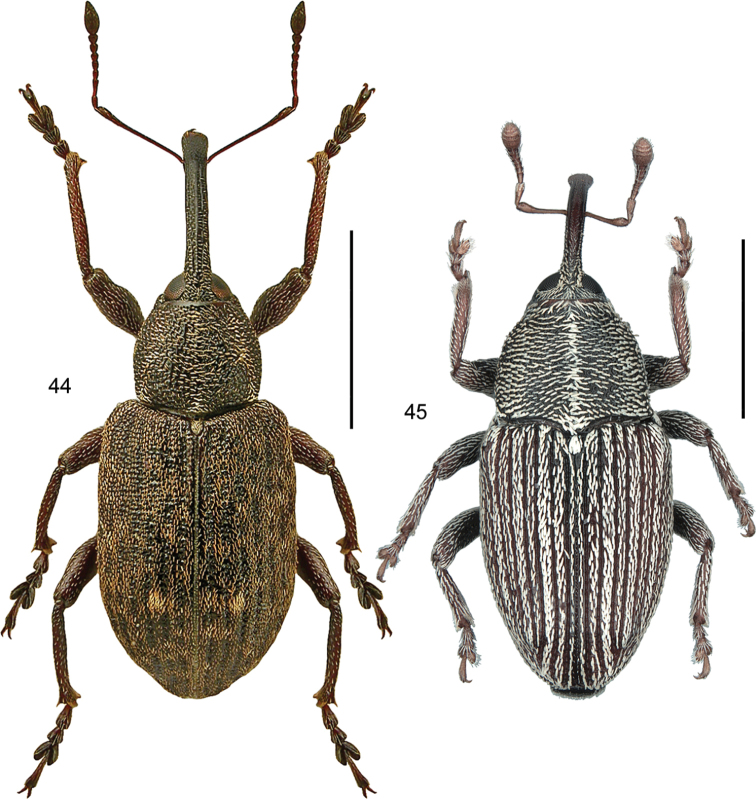
**44***Notarisscirpi* (Fabricius), habitus, L. Borowiec **45***Centrinopushelvinus* Casey, habitus. Scale bar: 2.0 mm (**44**), 1.0 mm (**45**).

(based on Hoffmann 1958). Body length: 4.7–7.0 mm. Habitus as in Fig. 44. Oblong-oval, black or brown, dorsal pubescence of small piliform scales more or less regularly distributed, with a speckled color pattern formed by patches of paler scales. Rostrum elongate, narrow, curved, punctate-striate and carinate. Prothorax approximately as long as wide, sides rounded, punctation dense and deep, with median line slightly elevated anteriorly. Elytra rounded at humeri in dorsal view, sides subparallel until slightly beyond middle. Ventrally with lateral portions of abdomen, metanepisternum, metanepimeron, and lateral portion of metaventrite with dense cream-colored scales.

####### Bionomic notes.

*Notarisscirpi* is oligophagous on *Scirpus* and *Carex* species in wet habitats (Koch 1992). Hoffmann (1958) notes that in France the species develops in the collar of *Carexacutiformis* Ehrh. and that adults can be collected in litter around wet areas.

####### Comments.

These are the first records of *Notarisscirpi* from the Nearctic region. After the identification of the DNA barcoded specimen deposited in CNC, 37 additional specimens from various localities in Quebec were found in other collections. The earliest record is from 1997, and the species seems to be firmly established in Quebec. *Notarisscirpi* is easily distinguished from *Tournotarisbimaculatus* (Fabricius, 1787) and *Notarispuncticollis* (LeConte, 1876), the two most similar species already known from North America, by the dense cream-colored scales on the lateral portions of the abdomen, metanepisternum, metanepimeron, and lateral portion of the metaventrite.

#### Baridinae

##### Madarini

###### Ampeloglypter sesostris

Taxon classificationAnimaliaColeopteraCurculionidae

(LeConte, 1876) 

792A7643-B894-55CC-901F-97D6E6134EF2

####### Distribution.

Native to the Nearctic region. Previously recorded from Indiana, Michigan, Ohio, Pennsylvania, Illinois, Florida, and Missouri (O’Brien and Wibmer 1982; Saunders and Tobin 2000) but likely more widespread in eastern and midwestern United States (Riedl and Taschenberg 1984).

####### Canadian records

(DNA barcoded specimens). Ontario: Pelee Island, 06-Jun-1982 (1 ex, CNC); Rouge National Urban Park, 25-Jun-2017 (1 ex, CBG).

####### Additional Canadian records.

Ontario: Pelee Island, 26-Jun-1940 (1 ex, CNC); Pelee Island, 27-Jun-1940 (1 ex, CNC); Windsor, 30-May-2002 (1 ex, CMNC).

####### Diagnostic information

(based on Blatchley and Leng 1916 and Anderson 2002). Body length: 2.7–3.0 mm. Body glabrous, shiny, elongate-oval, pale reddish brown throughout. Elytral interstriae flat. Femora not toothed. Tarsus with two claws connate at base.

####### Bionomic notes.

This species feeds on *Vitis* L. species, and it is considered a minor pest in vineyards (Bouchard et al. 2005). The female oviposits above a stem node and hollows out additional cavities along the longitudinal axis of shoots of the host plants. The larva develops and feeds on tissues inside the shoot, causing it to swell and thereby inducing gall formation (Lasnier et al. 2019).

####### Comments.

The red-brown *Ampeloglyptersesostris*, known commonly as the grape cane gallmaker, can be separated from the other two species in this genus in the United States and Canada by color: *A.ampelopsis* (Riley, 1869) and *A.longipennis* Casey, 1892 have a black integument.

#### Baridinae

##### Apostasimerini

###### Centrinopus helvinus

Taxon classificationAnimaliaColeopteraCurculionidae

Casey, 1892

F478DE75-633F-58D5-BE49-B672215BB975

Figure 45

####### Distribution.

Native to the Nearctic and Neotropical regions. Recorded from eastern and north central United States, and southward to Nicaragua (O’Brien and Wibmer 1982).

####### Canadian records.

Ontario: Waterloo, 21-Sep-2015 to 02-Oct-2015 (1 ex, CBG); Waterloo, 19-Sep-2016 to 30-Sep-2016 (1 ex, CBG).

####### Diagnostic information

(based on Casey 1920 and Anderson 2002). Body length: 2.0–2.7 mm. Body dark red-brown to black, oval, covered dorsally with pale scales, oriented perpendicularly to body axis on pronotum, oriented longitudinally on elytra (Fig. 45). Scales somewhat denser on elytral interstriae 3, 5, 7. Prothorax only slightly narrower than elytra in dorsal view. Scutellum densely covered with scales. Female with sharply defined longitudinal sulcus anterior to procoxae. Each procoxa in male with one anteriorly projecting spine-like process in front.

####### Bionomic notes.

Kissinger (1964) mentioned that adults in this genus are found on flowers of Asteraceae. According to Blatchley and Leng (1916)*Centrinopushelvinus* was taken on sweetscented joe pye weed, *Eutrochiumpurpureum* (L.) E.E. Lamont. We are not aware of any additional biological information published on this species. The barcoded Canadian specimens were collected with a Malaise trap on farmland.

####### Comments.

The genus *Centrinopus* Casey, 1892, which is in need of a taxonomic revision (Anderson 2002), contains six species in the eastern United States and is recorded here from Canada for the first time.

#### Ceutorhynchinae

##### Ceutorhynchini

###### Ceutorhynchus inaffectatus

Taxon classificationAnimaliaColeopteraCurculionidae

Gyllenhal, 1837

884D147A-B5CC-51EF-A997-9C092135CD4D

Figure 46

####### Distribution.

Native to the Palaearctic region. Widespread in Europe, recorded east to Kazakhstan and West Siberia (Alonso-Zarazaga et al. 2017). Adventive in the Nearctic region (Ontario, Canada).

####### Canadian records.

Ontario: Guelph, 02-Jun-2018 (1 ex, CBG).

####### Diagnostic information

**Figures 46–48.**
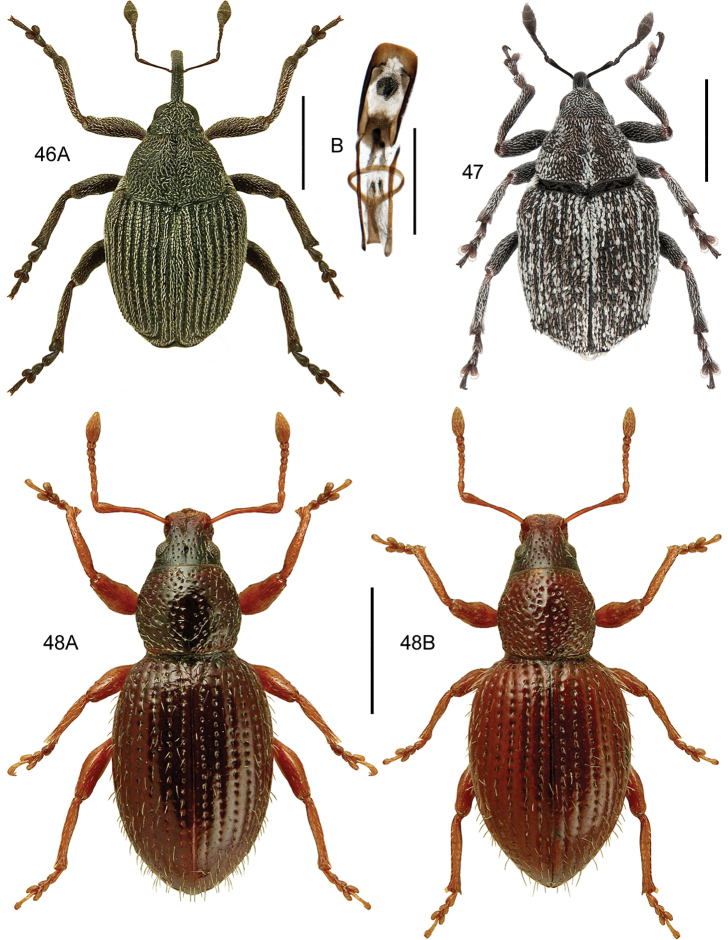
**46***Ceutorhynchusinaffectatus* Gyllenhal **46A** habitus, L. Borowiec **46B** aedeagus **47***Ceutorhynchusmutabilis* Dietz, habitus **48***Exomiastrichopterus* (Gautier des Cottes) **48A** male habitus, L. Borowiec **48B** female habitus, L. Borowiec. Scale bars: 1.0 mm (**46A; 47; 48**), 0.5 mm (**46B**).

(based on Lohse 1983). Body length 2.3–3.9 mm. Habitus as in Fig. 46A, appearing grey at low magnification due to the pale scales sparsely covering the black integument. Antennal funicle with seven antennomeres. Pronotum densely punctate, lateral tubercles absent. Elytra without apical calli, interstriae with narrow scales arranged in two or three longitudinal rows. Meso- and metafemora with small teeth. All tarsal claws with small basal tooth. Aedeagus as in Fig. 46B.

####### Bionomic notes.

This species feeds on *Hesperismatronalis* L. and *H.tristis* L. (Brassicaceae) (Koch 1992). The larvae develop in the seed pods, the adults feed on leaves and other parts of the host plants (Koch 1992, Larsen et al. 1992). The Canadian specimen was collected by sweep netting vegetation along a recreational trail where *H.matronalis* is abundant.

####### Comments.

*Ceutorhynchusinaffectatus* is similar in habitus to two other Palaearctic species adventive in North America: *C.obstrictus* (Marsham, 1802) and *C.rapae* Gyllenhal, 1837. These species have lateral pronotal tubercles, which are absent in *C.inaffectatus*. The combination of toothed femora, antennal funicle with seven antennomeres, pronotum lacking lateral tubercles and toothed tarsal claws will separate *C.inaffectatus* from native species of *Ceutorhynchus*. *Hesperismatronalis* (dame’s rocket or purple rocket) is an invasive weed in North America (Francis et al. 2009), and the strictly specialized *C.inaffectatus* could potentially be useful in its biological control.

###### Ceutorhynchus mutabilis

Taxon classificationAnimaliaColeopteraCurculionidae

Dietz, 1896

D0EDBAE9-AA63-5DED-BFE3-C66818B7A6C0

Figure 47

####### Distribution.

Native to the Nearctic region. This species is reported from Baja California, California, Oregon, Washington, Colorado, Kansas, and North Dakota in the United States (O’Brien and Wibmer 1982; Balsbaugh and Aarhus 1990).

####### Canadian records.

British Columbia: Radium, 24-Aug-1982 (2 exx, CNC); New Afton Mine, 20-Jun-2013 to 27-Jun-2013 (1 ex, CBG). Alberta: Calgary, 22-Jul-1976 (1 ex, CNC); Exact locality not specified, 20-Jun-1985 (1 ex, CNC); Exact locality not specified, 09-Jun-1990 (1 ex, CNC). Saskatchewan: Grasslands National Park, 19-Jul-2012 to 26-Jul-2012 (1 ex, CBG). Manitoba: Exact locality not specified, 24-Jul-1995 (1 ex, CNC).

####### Diagnostic information

(based on Scheibner 1963). Body length: 2.4 mm. Habitus as in Fig. 47, body with black integument covered with white to pale brown scales. Antennal funicle with seven antennomeres. Combination of two types of scales on pronotum and elytra, some broadly oval, others expanding from base with a truncate apex. Elytra with dense patch of appressed oval scales posterior to scutellum. Metafemora lacking tooth. Tarsal claws each with a small basal tooth.

####### Bionomic notes.

The natural history and host preferences of this little-studied species are unknown (Colonnelli 2004).

####### Comments.

Although this genus is in need of a revision, the combination of character states listed above, in combination with the habitus photograph (Fig. 47), should lead to the correct identification. Studies describing the biology of this and other native species of *Ceutorhynchus* Germar, 1824 are badly needed.

###### Peracalles pectoralis

Taxon classificationAnimaliaColeopteraCurculionidae

(LeConte, 1876)

AA1410D2-2A8A-51B7-9FFA-25426D0083E1

####### Distribution.

Native to the Nearctic region. Anderson (2002) reports this species from Illinois, Indiana, Ohio, Kentucky, and Missouri in the United States.

####### Canadian records.

Ontario: Point Pelee National Park, 06-Jul-2015 (1 ex, CBG); Point Pelee National Park, 16-Jun-2014 to 22-Jun-2014 (3 exx, CBG).

####### Diagnostic information

(based on Anderson 2002). Body length: 3.0–3.3 mm. Body black, covered with broad, flat, appressed dark brown to pale scales, broadly oval in dorsal view. Antennal funicle with seven articles. Prosternum with deep longitudinal sulcus for reception of rostrum, sulcus extending posteriorly to anterior edge of mesoventrite. Elytra strongly convex in lateral view, with a single row of flat, apically truncate, erect scales on each interstria.

####### Bionomic notes.

Adults in this genus occur in leaf litter (Anderson 2002). The Canadian specimens were collected from a marsh and a swampy forest using pan traps, pitfall traps and Berlese funnel extraction.

####### Comments.

The genus *Peracalles* Kissinger, 1964 contains two species in the United States (Anderson 2002) and is recorded here in Canada for the first time.

##### Entiminae

##### Sciaphilini

###### Exomias trichopterus

Taxon classificationAnimaliaColeopteraCurculionidae

(Gautier des Cottes, 1863)

F5180719-78EE-5A3E-8A14-E250180FC289

Figure 48

####### Distribution.

Native to the Palaearctic region. Widespread in Central Europe (Alonso-Zarazaga et al. 2017). Adventive in the Nearctic region (Ontario, Canada).

####### Canadian records.

Ontario: Rouge National Urban Park, 24-Jun-2017 to 25-Jun-2017 (1 ex, CBG).

####### Diagnostic information

(based on Rheinheimer and Hassler 2013). Body length: 2.7–3.4 mm. Habitus as in Fig. 48A, B. Body brown to black, covered with fine semi-erect to erect setae, legs pale yellow to red-brown. Rostrum with shallow longitudinal depression dorsally. Globose elytra with humeral angles obsolete. Elytra lacking row of long setae near suture on posterior half.

####### Bionomic notes.

This common European species is polyphagous on herbaceous plants (Balalaikins 2011) and could become a new pest of berry crops in Canada (see Kolov and Korotyaev 2017).

####### Comments.

*Exomiastrichopterus* is very similar in appearance to *E.pelluciduspellucidus* (Boheman, 1834), another adventive Palaearctic species which is common and widespread in North America. Both species were previously placed in the genus BarypeithesJacquelin du Val, 1854. The formersubgenusExomias was elevated to the generic level by Borovec (2013). *Exomiaspelluciduspellucidus* can be diagnosed primarily by the noticeably denser setae on their elytra, especially near the apex where an additional row of long setae is present along the elytral suture.

##### Scolytinae

##### Xyleborini

###### Ambrosiodmus rubricollis

Taxon classificationAnimaliaColeopteraCurculionidae

(Eichhoff, 1875)

B59C424A-FF37-5320-8EB8-3B226089B554

####### Distribution.

Native to the eastern Palaearctic and Oriental regions (Knížek 2011). Adventive in Europe, Australia, and the Nearctic region (widespread in the United States; Ontario, Canada) (Knížek 2011; Gomez et al. 2018).

####### Canadian records.

Ontario: Point Pelee National Park, 11-Jul-2012 to 18-Jul-2012 (1 ex, CBG); Point Pelee National Park, 16-Jun-2014 to 22-Jun-2014 (2 exx, CBG).

####### Diagnostic information

(based on Gomez et al. 2018). Body length 2.4–2.6 mm. Pronotum with asperities covering entire surface. Elytral declivity with tubercles on interstriae 2 as large as those on interstriae 1 and 3.

####### Bionomic notes.

*Ambrosiodmusrubricollis* uses symbiotic fungi to attack many genera of gymnosperm and dicot trees including species in the following Canadian genera: *Abies* Mill., *Aesculus* L., *Alnus* Mill., *Carya* Nutt., *Cornus* L., *Fraxinus* L., *Ilex* L., *Juglans* L., *Morus* L., *Pinus* L., *Populus* L., *Prunus* L., *Quercus* L., *Rhus* L. (Faccoli et al. 2009). One of the Canadian specimens was collected with a Malaise trap in a savanna, the two others were caught with pitfall traps in a swampy forest.

####### Comments.

This is the only *Ambrosiodmus* species known from Canada, although two larger-bodied species are known from states bordering southern Ontario (Gomez et al. 2018). *Ambrosiodmuslewisi* (Blandford, 1984), and *A.tachygraphus* (Zimmermann, 1868) can be distinguished from *A.rubricollis* by their greater body lengths (3.6 to 4.0 mm).

## Discussion

This study adds 60 new species to the Canadian beetle fauna and resolves taxonomic confusion in another three species. Among the 42 adventive species covered, 40 are native to the Palaearctic region. The remaining two species, *Clambussimsoni* and *Attagenussmirnovi*, are native to the Australian and Afrotropical regions respectively, but also occur in the Palaearctic as adventive species. *Nephusbisignatus* and *Dichelotarsuslapponicus* were previously known only from the Palaearctic region, but because they were collected in remote arctic localities in Canada, we consider it likely that they are Holarctic taxa whose occurrence in North America was previously overlooked. The remaining 19 new species are native to North America, and represent either previously overlooked occurrences in Canada, or recent range expansions. The fact that many new records of native species were of species that are difficult to identify by morphological methods suggests that most of these species have been long present in Canada but overlooked. Six species were found at Point Pelee, a forest and wetland area isolated from similar habitats in both United States and Canada, further suggesting that recent range extensions are an unlikely explanation for new Canadian records of these species. The fact that 54 of the 60 new species for Canada were found in general survey samples for insects clearly indicates that much more work is needed using specialized, taxon-specific collecting techniques to achieve a full inventory of the Coleoptera diversity in Canada. We also expect that increased insect survey activity in United States would recover records for many of the same adventive species there, plus additional species as-yet unrecorded from North America.

Species that are adapted to disturbed or ruderal habitats are more likely to be accidentally transported through human activities than species that require non-synanthropic habitats (Lockwood et al. 2013). Many of the adventive beetle species established in North America are strongly synanthropic and occur mainly in human-disturbed habitats and settlements (Klimaszewski et al. 2010, 2012). Not surprisingly, most of the new adventive species we report here were found mainly or exclusively in Southern Ontario, which is Canada’s most densely populated and biodiverse region, and in the Greater Vancouver area, the third largest metropolitan area in Canada and home to the busiest port in the country (also with high native insect diversity). Some of these adventive species have likely been present in Canada for a long time, but have been overlooked due to difficulties in morphological identification. *Stenichnusscutellaris* and *Amischadecipiens* are widespread and common in southern Ontario, while *A.decipiens* is also found in the Greater Vancouver area. *Malthodespumilus* occurs from East to West in both suburban and natural environments. All three species represent genera that have received little or no taxonomic investigation in North America in recent decades. Others, such as *Calyptomerusdubius*, *Clambussimsoni*, *Litargusconnexus*, and *Olibrusliquidus*, may have arrived more recently as they have only been found in one or a few urban localities, and some are only represented by singleton specimens in the Canadian DNA barcode data. Verifying that these species are well-established in Canada will require further monitoring and study of material in existing collections.

Most of the species newly recorded here that are shared between Europe and North America probably arrived into North America from Europe because they were discovered there first. Relatively few North American beetle species are known to occur as adventive in Europe, but more may well be uncovered especially in families where the Nearctic fauna is poorly known. Adventive insect species are sometimes described as new to science after arriving in a new area (Wheeler and Hoebeke 2009), as exemplified by the two new synonymies in Staphylinidae we establish in this study. New species are less likely to escape notice in Europe where the beetle fauna, including taxonomically challenging families such as Staphylinidae, is generally better known and more intensively studied compared to North America. Our analysis of European and Canadian DNA barcode data has uncovered at least one native North American species of Staphylinidae occurring as adventive in Europe and described as new from there. This synonymy will be formally established in a future publication. A geographically well represented DNA barcode dataset can provide information on the biogeography and distributional status (native vs. adventive) of species, and potentially identify the geographic origin of adventive or expansive species (Valade et al. 2009; Lees et al. 2011). A detailed analysis of the spatial genetic variation in all the species covered here is beyond the scope of the present paper but will be a subject of future studies.

It is noteworthy that 57 of our 60 new species records for Canada were discovered, in whole or in part, using material recently collected by the Centre for Biodiversity Genomics. In fact, only two of the new species were discovered based solely on specimens from the CNC (see Table 1). The CNC was once the primary source of new data on Canadian insect species, but it is no longer the depository for most specimens from general survey and inventory work across the country. For example, the 1,085,146 Canadian insect specimens analyzed by Hebert et al. (2016) are stored in the CBG voucher archive. To further illustrate this change in specimen accumulation, a complete inventory of the Canadian Scarabaeoidea in the CNC (ABTS, unpublished data) revealed that only 7% of the specimens in that institution were collected during the past 30 years. In contrast, 37% of the specimens in CNC were collected during the previous 30-year period (1959–1988). Inventories of Canadian Scarabaeoidea in most of the major entomological collections across the country show a similar overall pattern. Although general survey and inventory work is badly needed in Canada to detect the full diversity of the Coleoptera fauna, collecting efforts have significantly declined over the past 30 years. This leaves invasive species undetected and range expansions undiscovered for years longer than would have been the case when there were large-scale survey and inventory efforts (e.g., Northern Insect Survey; Lonsdale and Huber 2011). With the growing threat of invasive species through increasing global trade and the northward expansion of species due to the changing climate, ongoing collaborative survey and inventory efforts are needed to detect new species as they appear in Canada.

Bousquet et al. (2013) recorded 8237 Coleoptera species in Canada, an increase of almost 10% over a similar checklist published 22 years earlier (Bousquet 1991). The recent summary by Brunke et al. (2019) increased the number of Canadian beetle species to 8302. These increases are mainly due to progress in taxonomic research on Coleoptera species already present in Canada, but species recently establishing themselves in Canada also increased the count. The 60 species we report here as new for Canada increase the number of known beetle species in the country by another 0.7% compared to Brunke et al. (2019). Of these, the 40 new adventive species add 6.3% to the number of non-native Coleoptera known from Canada. Further new Canadian records and new synonymies in European and North American Coleoptera detected through DNA barcode data are currently being validated. Our study shows that DNA barcoding, combined with morphological validation of the voucher specimens, is a powerful tool for detecting and identifying overlooked or recently arrived species, both native and adventive (see also deWaard et al. 2009, Landry et al. 2013). Even though the species coverage of the European and Canadian DNA barcode reference libraries of beetles is still far from complete, our results undeniably demonstrate their usefulness for cataloguing regional biodiversity.

## Supplementary Material

XML Treatment for Dineutus emarginatus

XML Treatment for Anisodactylus caenus

XML Treatment for Coelostoma orbiculare

XML Treatment for Leiodes polita

XML Treatment for Bibloplectus minutissimus

XML Treatment for Mycetoporus reichei

XML Treatment for Tachyporus atriceps

XML Treatment for Amischa decipiens

XML Treatment for Atheta vaga

XML Treatment for Myllaena infuscata

XML Treatment for Bledius gallicus

XML Treatment for Carpelimus elongatulus

XML Treatment for Stenichnus collaris

XML Treatment for Stenichnus scutellaris

XML Treatment for Scydmaenus rufus

XML Treatment for Scydmoraphes minutus

XML Treatment for Lathrobium geminum

XML Treatment for Lathrobium lineatocolle

XML Treatment for Medon apicalis

XML Treatment for Medon ripicola

XML Treatment for Pseudomedon obscurellus

XML Treatment for Phyllophaga implicita

XML Treatment for Calyptomerus dubius

XML Treatment for Clambus simsoni

XML Treatment for Contacyphon fuscescens

XML Treatment for Contacyphon kongsbergensis

XML Treatment for Contacyphon obscurellus

XML Treatment for Aulonothroscus distans

XML Treatment for Trixagus carinifrons

XML Treatment for Trixagus meybohmi

XML Treatment for Pseudanostirus tigrinus

XML Treatment for Dichelotarsus lapponicus

XML Treatment for Malthodes pumilus

XML Treatment for Attagenus smirnovi

XML Treatment for Petalium incisum

XML Treatment for Cryptophilus obliteratus

XML Treatment for Cryptophilus propinquus

XML Treatment for Henoticus mycetoecus

XML Treatment for Acylomus ergoti

XML Treatment for Olibrus liquidus

XML Treatment for Epuraea unicolor

XML Treatment for Chilocorus renipustulatus

XML Treatment for Nephus bisignatus

XML Treatment for Scymnus rubromaculatus

XML Treatment for Orthoperus corticalis

XML Treatment for Litargus connexus

XML Treatment for Cis boleti

XML Treatment for Cis glabratus

XML Treatment for Mordellistena militaris

XML Treatment for Lasconotus subcostulatus

XML Treatment for Isomira angusta

XML Treatment for Chaetocnema hortensis

XML Treatment for Longitarsus lewisii

XML Treatment for Lythraria salicariae

XML Treatment for Scelolyperus liriophilus

XML Treatment for Notaris scirpi

XML Treatment for Ampeloglypter sesostris

XML Treatment for Centrinopus helvinus

XML Treatment for Ceutorhynchus inaffectatus

XML Treatment for Ceutorhynchus mutabilis

XML Treatment for Peracalles pectoralis

XML Treatment for Exomias trichopterus

XML Treatment for Ambrosiodmus rubricollis

## Supplementary materials

Supplementary material 1 Table S1. Detailed collection data, GenBank accession numbers, and institutional storage information of the studied specimens Data type: species data https://binary.pensoft.net/file/362164This dataset is made available under the Open Database License (http://opendatacommons.org/licenses/odbl/1.0/). The Open Database License (ODbL) is a license agreement intended to allow users to freely share, modify, and use this Dataset while maintaining this same freedom for others, provided that the original source and author(s) are credited.Mikko Pentinsaari, Robert Anderson, Lech Borowiec, Patrice Bouchard, Adam Brunke, Hume Douglas, Andrew B.T. Smith, Paul D. N. Hebert
